# Deep proteomic profiling unveils arylsulfatase A as a non-alcoholic steatohepatitis inducible hepatokine and regulator of glycemic control

**DOI:** 10.1038/s41467-022-28889-2

**Published:** 2022-03-10

**Authors:** Magdalene K. Montgomery, Jacqueline Bayliss, Shuai Nie, William De Nardo, Stacey N. Keenan, Paula M. Miotto, Hamzeh Karimkhanloo, Cheng Huang, Ralf B. Schittenhelm, Anthony S. Don, Andrew Ryan, Nicholas A. Williamson, Geraldine J. Ooi, Wendy A. Brown, Paul R. Burton, Benjamin L. Parker, Matthew J. Watt

**Affiliations:** 1 Department of Anatomy and Physiology, School of Biomedical Sciences, Faculty of Medicine Dentistry and Health Sciences, University of Melbourne, Melbourne, VIC 3010 Australia; 2 Melbourne Mass Spectrometry and Proteomics Facility, Bio21 Molecular Science & Biotechnology Institute, The University of Melbourne, Melbourne, VIC 3010 Australia; 3 Metabolism, Diabetes and Obesity Program, Monash Biomedicine Discovery Institute, and Department of Physiology, Monash University, Melbourne, VIC 3800 Australia; 4 Proteomics & Metabolomics Facility, Monash University, Melbourne, VIC 3800 Australia; 5 School of Medical Sciences, Faculty of Medicine and Health, The University of Sydney, Camperdown, NSW 2050 Australia; 6 TissuPath, Mount Waverley, VIC 3149 Australia; 7 Centre for Obesity Research and Education, Department of Surgery, Monash University, Melbourne, VIC 3004 Australia

**Keywords:** Metabolic syndrome, Diabetes, Non-alcoholic steatohepatitis, Fat metabolism

## Abstract

Non-alcoholic steatohepatitis (NASH) and type 2 diabetes are closely linked, yet the pathophysiological mechanisms underpinning this bidirectional relationship remain unresolved. Using proteomic approaches, we interrogate hepatocyte protein secretion in two models of murine NASH to understand how liver-derived factors modulate lipid metabolism and insulin sensitivity in peripheral tissues. We reveal striking hepatokine remodelling that is associated with insulin resistance and maladaptive lipid metabolism, and identify arylsulfatase A (ARSA) as a hepatokine that is upregulated in NASH and type 2 diabetes. Mechanistically, hepatic ARSA reduces sulfatide content and increases lysophosphatidylcholine (LPC) accumulation within lipid rafts and suppresses LPC secretion from the liver, thereby lowering circulating LPC and lysophosphatidic acid (LPA) levels. Reduced LPA is linked to improvements in skeletal muscle insulin sensitivity and systemic glycemic control. Hepatic silencing of *Arsa* or inactivation of ARSA’s enzymatic activity reverses these effects. Together, this study provides a unique resource describing global changes in hepatokine secretion in NASH, and identifies ARSA as a regulator of liver to muscle communication and as a potential therapeutic target for type 2 diabetes.

## Abstract (web summary)

Non-alcoholic steatohepatitis (NASH) and type 2 diabetes are closely linked, but the connecting pathophysiological mechanisms are incompletely understood. Here the authors identify arylsulfatase A as a NASH-induced hepatokine that inhibits hepatic lysophosphatidylcholine and lysophosphatidic acid secretion, and improves muscle insulin action and systemic glucose homeostasis.

## Introduction

Nonalcoholic fatty liver disease (NAFLD) is the world’s most common chronic liver condition^1^ and ranges from simple steatosis to non-alcoholic steatohepatitis (NASH) with or without hepatic fibrosis, in the absence of excessive alcohol intake. While steatosis is not associated with a significant increase in liver-related morbidity or mortality^2^, NASH can progress to more severe diseases such as cirrhosis and hepatocellular carcinoma, leading to liver failure and liver transplantation^2,3^. NASH increases in parallel with obesity and type 2 diabetes mellitus (T2D), with prevalence estimated at 57–64% in obesity and 37% in T2D, an incidence that dramatically exceeds the 3–5% observed in the general population^2,4^. Despite these strong associations, the factors linking NASH with these metabolic diseases are incompletely understood.

The liver is an important regulator of metabolism and plays a key role in cholesterol, triglyceride, and glucose metabolism by integrating neural, endocrine, and nutrient signals to invoke well-characterized intracellular signaling pathways^5,6^. The liver also regulates local and systemic metabolism via the secretion of proteins, which are collectively termed hepatokines^7^. We previously reported secretion of 538 proteins from hepatocytes and showed substantial remodeling of hepatokine secretion with simple steatosis^8^. Notably, secreted factors from steatotic hepatocytes induced pro-inflammatory signaling and insulin resistance in recipient cells. Given the close links between NASH and other metabolic diseases, we hypothesized that hepatokine secretion is altered in NASH and that NASH-induced proteins induce autocrine/paracrine and endocrine signaling to alter local and systemic metabolism. Further understanding of the proteins secreted by the liver could facilitate a deeper understanding of inter-tissue communication and identify targets for future diagnostics and therapies for various diseases.

Accordingly, we used liquid chromatography coupled to tandem mass spectrometry (LC-MS/MS) profiling to assess the impact of NASH on hepatokine secretion and herein report striking hepatokine remodeling in two experimental models of NASH. We identified 1536 hepatocyte-secreted proteins common to both models, of which 344 were classically secreted, and 33 of these proteins were differentially secreted in NASH. From this analysis, we identified arylsulfatase A (ARSA) as a hepatocyte-secreted protein that is regulated in NASH.

Arylsulfatases are lysosomal enzymes that play important roles in sphingolipid metabolism. ARSA catalyzes the conversion of sulfatides to galactosylceramides, with congenital ARSA deficiencies in humans leading to metachromatic leukodystrophy, a disease characterized by sulfatide accumulation in the myelin sheath within the brain and progressive neurological defects^9^. The presence of a N-terminal signal peptide in ARSA suggests that ARSA is a classically secreted protein and ARSA has been detected in murine^10^ and human plasma^11,12^. Circulating ARSA can be endocytosed through the ubiquitously expressed mannose-6-phosphate receptor^13^, with the highest uptake into the liver^10^, suggesting a plausible role of circulating ARSA in regulating cellular functions, particularly in the liver. In this work, we identify ARSA as a NASH-inducible hepatokine and powerful modulator of hepatic sulfatide and lysophospholipid metabolism, skeletal muscle insulin action, and whole-body glycemic control.

## Results

### Induction of NASH in mice

**Fig. 1:**
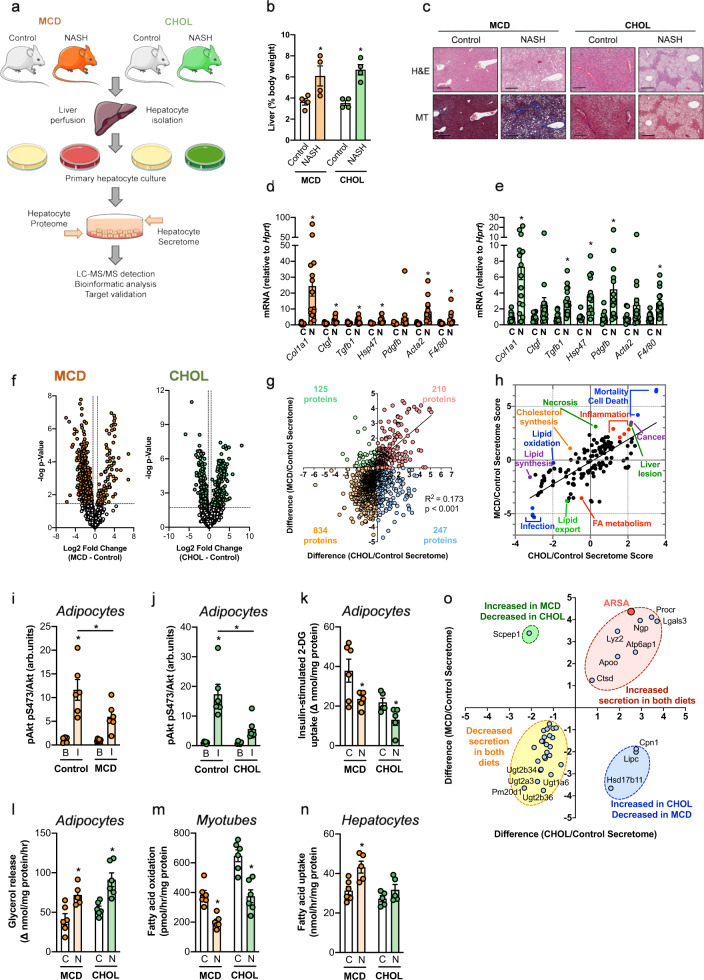
Identification and characterization of NASH-secreted proteins. C57BL/6 mice were fed either a methionine- and choline-deficient diet (MCD, orange) or a diet enriched in lipid, fructose, and cholesterol (CHOL, green). Control mice received standard diet. **a** Hepatocytes from MCD and CHOL mice (and respective Controls) were isolated, followed by assessment of the intracellular proteome and secretome. **b** Liver weight (*n* = 4/group). *P = 0.049 MCD and *P = 0.011 CHOL Control vs. NASH, by unpaired two-tailed *t* test. **c** Representative liver histology (H&E and Masson’s Trichrome staining). Scale bar = 200 μm. Liver histology was assessed in three MCD Control, three MCD, four CHOL Control, four CHOL, with similar results obtained. **d**, **e** Hepatocyte mRNA expression of fibrosis and macrophage markers ((**d**) Control, *n* = 14/gene; MCD, *n* = 13/gene (**d**) MCD Pdgfb, *n* = 10 (**e**) Control, *n* = 9/gene (**e**) Control Pdgfb, *n* = 15 (**e**) CHOL), *n* = 10/gene). **d** *P = 0.002 Col1a1, *P = 0.008 Ctgf, *P = 0.005 Tgfb1, *P = 0.0002 Hsp47, *P* = 0.178 Pdgfb, *P = 0.002 Acta2, *P* = 0.054 F4/80 Control vs. NASH, by unpaired two-tailed *t* tests. **e** *P = 0.004 Col1a1, *P* = 0.184 Ctgf, *P = 0.002 Tgfb1, *P = 0.002 Hsp47, *P = 0.037 Pdgfb, *P* = 0.176 Acta2, *P = 0.003 F4/80 Control vs. NASH, by unpaired two-tailed *t* tests. **f** Volcano plots showing significant NASH-regulated secreted proteins from hepatocytes of MCD and CHOL-fed mice vs. Control mice. **g** Correlation of NASH-regulated changes in protein secretion from MCD and CHOL mice. **h** Ingenuity Pathway Analysis showing NASH-induced changes in diseases and functions. **i**–**n** Medium containing secreted factors derived from MCD (orange) and CHOL (green) hepatocytes was obtained, then applied to 3T3-L1 adipocytes, C2C12 myotubes and primary murine hepatocytes, followed by assessment of (**i**, **j**) basal and insulin-stimulated Akt S473 phosphorylation (*n* = 6/group), **k** 1-^14^C-2-deoxyglucose uptake in adipocytes (calculated as Δ, insulin-stimulated—basal) (*n* = 6/group for MCD, *n* = 5/group for CHOL), **l** adipocyte lipolysis (shown as Δ, isoproterenol-stimulated—basal) (*n* = 6/group, except MCD *n* = 5), **m** fatty acid oxidation in myotubes (*n* = 5/group), and **n** fatty acid uptake in hepatocytes (*n* = 6/group, except CHOL Control *n* = 5). C = control conditioned media; *N* = NASH conditioned media. For panel **i**, *P < 0.0001 Control and *P* = 0.064 MCD basal vs. insulin, adjoining line Control vs. NASH by two-way ANOVA and Bonferroni post hoc analysis (*P* = 0.027). For panel **j**, *P < 0.0001 Control and *P* = 0.548 CHOL Basal vs. Insulin, adjoining line Control vs. NASH by two-way ANOVA and Bonferroni post hoc analysis (*P* = 0.001). For panels **k**–**n**, *P < 0.05 by unpaired two-tailed *t* tests (**k**: *P = 0.041 MCD, *P = 0.042 CHOL; **l**: *P = 0.009 MCD, *P = 0.003 CHOL; **m**: *P < 0.0001 MCD, *P = 0.001 CHOL; N: *P = 0.009 CHOL, *P* = 0.178 MCD). For conditioned media experiments, each data point was obtained using secretion media from an individual mouse. **o** Classically secreted proteins significantly (*P* < 0.05, >twofold change) regulated by both MCD and CHOL dietary regimes. For panels **d**, **e** and **i**–**n**, data are means ± SEM. Source data are provided as a Source Data file. 2-DG 2-deoxyglucose, Acta2 smooth muscle alpha (α)-2 actin, Apoo apolipoprotein O, Arsa arylsulfatase A, Atp6ap1 ATPase H + transporting accessory protein 1, B Basal, C Control, CHOL diet enriched in lipid, sucrose, fructose and cholesterol, Col1a1 collagen type I alpha 1 chain, Cpn1 carboxypeptidase N subunit 1, Ctgf connective tissue growth factor, Ctsd cathepsin D, F4/80 adhesion G protein-coupled receptor E1 (ADGRE1), H&E hematoxylin and eosin, Hsp47 heat shock protein47, Hsd17b11 hydroxysteroid 17-beta dehydrogenase 11, I insulin, Lgals3 galectin 3, Lipc hepatic triacylglycerol lipase, MCD methionine–choline deficient, MT Masson’s Trichrome, N NASH, Ngp neutrophilic granule protein, Pdgfb platelet-derived growth factor, Pm20d1 peptidase M20 domain containing 1, Procr protein C receptor, Scpep1 serine carboxypeptidase 1, Tgfb1 transforming growth factor-beta, Ugt1a6 UDP glucuronosyltransferase 1 family member A6, Ugt2a3 UDP glucuronosyltransferase 2 family member A3, Ugt2b34 UDP glucuronosyltransferase 2 family member B34, Ugt2b36 UDP glucuronosyltransferase 2 family member B36.

NASH was induced by feeding mice a methionine–choline-deficient (MCD) diet for 12 weeks or a high-fat, high-fructose diet enriched with 2% cholesterol (CHOL) for 10 months (Fig. 1a). As expected, the livers of mice fed the NASH diets were heavier compared with age-matched mice fed a control diet (Fig. 1b) and histopathological assessment showed increased steatosis, lobular inflammation, and hepatocyte ballooning, and thereby a significant increase in the NAFLD activity score in mice fed the NASH diets (Fig. 1c and Supplementary Table S1). This was accompanied by increased expression of molecular markers of inflammation and fibrosis including *Col1a1, Ctgf, Tgfb1*, and *Hsp47* (Fig. 1d, e) and extensive fibrosis was confirmed in mice fed the MCD, with less fibrosis in mice fed the CHOL diet (Fig. 1c). Mice underwent extensive metabolic phenotyping and body weight, fat and lean mass, tissue weights, energy expenditure, and plasma lipids, ketone bodies, and inflammatory markers are shown in Supplementary Fig. S1A–J, Supplementary Table S2, and Supplementary Table S3.

### NASH alters the hepatocyte proteome and hepatokine secretion

We currently lack a deep understanding of the effects of NASH on the composition of proteins secreted by the liver. We therefore performed LC-MS/MS analysis of intracellular and secreted proteins from hepatocytes isolated from livers of mice fed Control and NASH-inducing diets (Fig. 1a). A total of 2855 proteins were identified in all conditions: 2463 and 2286 proteins were identified in the MCD/Control and CHOL/Control intracellular proteomes, respectively, and 1518 and 1536 secreted proteins were identified in MCD/Control and CHOL/Control (see Supplementary Excel file for the full list of proteins). The number of hepatocyte-secreted proteins identified in this analysis is substantially higher than previously reported using similar models (i.e., 538 and 1149)^8,14^ or when predicted from transcriptomic analysis (i.e., 305)^15^. Of the secreted proteins identified here, 344 (22.6%, MCD) and 343 (22.3%, CHOL) were denoted to contain a N-terminal signal peptide, indicating that ~22% of hepatocyte-secreted proteins are classically secreted.

The hepatocyte intracellular proteome was substantially remodeled in both diets with principal component analysis (PCA) revealing robust clustering of the groups with 991 (40% of total, MCD) and 1348 proteins (59% of total, CHOL) significantly altered with NASH (Supplementary Fig. S1K–L). Similarly, the composition of hepatocyte-secreted proteins was markedly affected by NASH, with a clear clustering of the groups in PCA, and 274 (18% of total) and 278 (18% of total) significantly altered in the MCD and CHOL mice, respectively (Fig. 1f and Supplementary Fig. S1K). The NASH-induced changes in protein secretion were positively correlated between MCD and CHOL diets (*R*^2^ = 0.173, *P* < 0.001) (Fig. 1g).

Ingenuity Pathway Analysis of the diseases and functions associated with the hepatocyte-secreted proteins affected by NASH identified a substantial overlap in both diets. NASH-regulated proteins were associated with increased liver lesions, inflammation, cancer, mortality/cell death, and decreased lipid metabolism, including decreased lipid export and fatty acid metabolism (Fig. 1h). Further comparisons of canonical pathways, diseases, and functions, as well as potential upstream regulators for both the NASH intracellular proteomes and secretomes are presented in Supplementary Fig. S1M, N. Of note, changes in the intracellular proteome predicted increased integrin and IL8 signaling (Supplementary Fig. S1M), which are emerging as important drivers of hepatic fibrosis^16,17^.

### NASH-secreted factors induce insulin resistance and defects in lipid metabolism

NASH is closely associated with T2D^4^ and insulin resistance is a key feature of T2D pathogenesis^18^. To test whether the secreted products from the livers of mice with NASH induce insulin resistance, hepatocytes were isolated from mice fed the Control and NASH-inducing diets, the medium containing hepatocyte-secreted factors was collected (Fig. 1a) and applied to 3T3-L1 murine adipocytes, fully differentiated C2C12 murine myotubes and primary murine hepatocytes. NASH-secreted factors from both dietary regimes reduced insulin-stimulated phosphorylation of protein kinase Akt, the insulin receptor (IR) and/or insulin receptor substrate 1 (IRS1) in all cell types (Fig. 1i, j and Supplementary Fig. S1O–Q). Functional analysis in adipocytes showed that the NASH hepatocyte medium induced a ~40% decrease in insulin-stimulated 2-deoxyglucose uptake (Fig. 1k). These results demonstrate that the factors secreted from hepatocytes derived from livers of mice with NASH induce insulin resistance. In addition, NASH-secreted factors from both dietary regimes increased adipocyte lipolysis (Fig. 1l), reduced fatty acid oxidation in myotubes (Fig. 1m) and increased fatty acid uptake in hepatocytes (Fig. 1n), recapitulating key metabolic defects commonly observed in NASH^19^.

### Identification of NASH-regulated proteins

The overarching objective of this study was to identify classically secreted hepatokines that are regulated in NASH and play a key role in the regulation of insulin sensitivity and whole-body glycemic control. Of the ~275 classically secreted proteins impacted by NASH (Fig. 1f), 25 proteins were significantly decreased by >twofold, and eight proteins were significantly increased by >twofold in both NASH models (Fig. 1o: *q* < 0.05, *t* test with FDR cutoff 0.05, Benjamini Hochberg). Of the significantly decreased proteins, nine are members of the uridine 5’-diphospho-glucuronosyltransferase (UGT) family, proteins that play an essential role in hepatic detoxification; four belong to the carboxylesterase enzyme family (Ces1d, Ces1f, Ces2a, Ces3b), which are involved in the degradation of acylglycerols; and two are protein disulfide isomerases (Pdia3 and Pdia5), which catalyze the rearrangement of disulfide bonds in proteins, are ER chaperones^20^ and were previously reported in a hepatokine screen^8^.

Of the eight proteins that were increased in secretion with both NASH diets, we were particularly interested in arylsulfatase a (ARSA), a lysosomal protein that degrades the glycosphingolipid sulfatides to galactosylceramides, which can be further degraded to ceramides. Sphingolipids such as ceramides are well-known inhibitors of insulin action^21^ and there is some evidence that complex sphingolipids also interfere with insulin action and glycemic control^22^. In light of these interesting associations and the lack of knowledge regarding ARSA’s circulating functions, we next sought to understand the metabolic role of liver-derived ARSA.

### Arylsulfatase A is increased in murine and human NAFLD/NASH

**Fig. 2:**
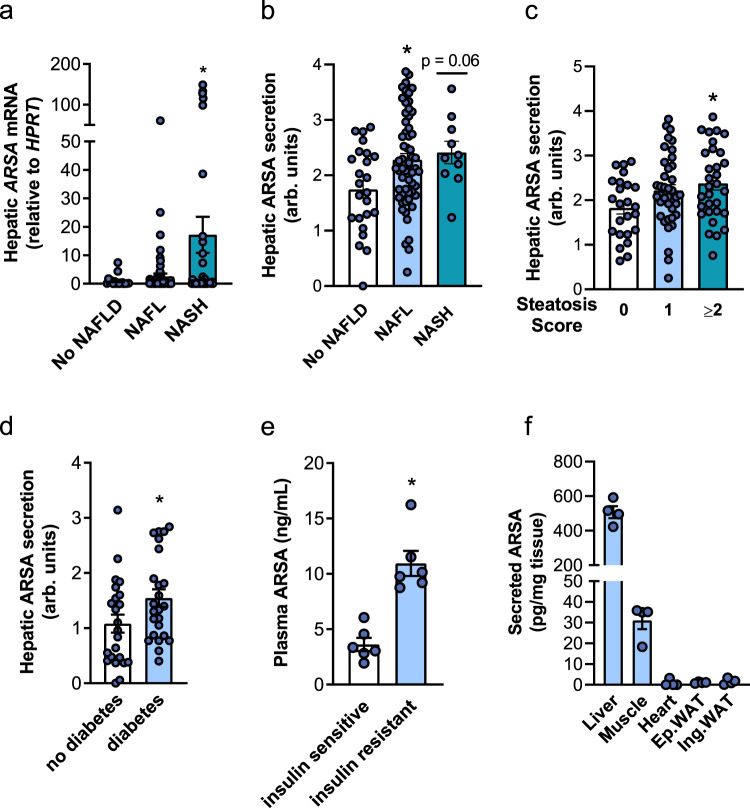
ARSA expression and secretion in humans with NAFLD/NASH and type 2 diabetes. **a** ARSA mRNA expression in patients grouped by no adverse pathology (i.e., No NAFLD, *n* = 17, white bar), NAFL (*n* = 66, light-blue bar) or NASH (*n* = 43, dark-blue bar). *P = 0.047 No NAFLD vs. NASH. **b**–**d** ARSA secretion from precision-cut liver slices in patients grouped by (**b**) No NAFLD (*n* = 24, white bar), NAFL (*n* = 58, light-blue bar) or NASH (*n* = 10, dark-blue bar). *P = 0.016 No NAFLD vs. NAFL, *P* = 0.064 No NAFLD vs NASH. **c** steatosis score (0, *n* = 24; 1, *n* = 39; ≥2, *n* = 31), *P* = 0.112 score 0 vs. score 1, *P = 0.022 score 0 vs. score >2. **d** type 2 diabetes status (no diabetes *n* = 23, white bar; diabetes *n* = 24, blue bar), *P = 0.047 no diabetes vs. diabetes. **e** Plasma ARSA in insulin-sensitive chow-fed mice (white bar) and age-matched obese insulin-resistant mice fed a high-fat diet for 8 weeks (blue bar) (*n* = 6/group), *P = 0.0002. **f** ARSA secretion from mouse precision-cut liver slices, intact tendon-to-tendon extensor digitorum longus (EDL) and soleus skeletal muscles (combined conditioned media denoted as muscle), precision-cut heart slices, and epididymal and inguinal adipose tissues (*n* = 4/group). For all panels, data are means ± SEM. *P < 0.05 vs. No NAFLD, no diabetes, insulin-sensitive as assessed by one-way analysis of variance (ANOVA) and Bonferroni post hoc analysis (**a**–**c**) or two-tailed unpaired *t* tests (**d**, **e**). Source data are provided as a Source Data file. Ep.WAT epididymal white adipose tissue, Ing.WAT inguinal white adipose tissue, NAFL nonalcoholic fatty liver, NAFLD nonalcoholic fatty liver disease, NASH nonalcoholic steatohepatitis.

We investigated whether the NASH-induced increase in ARSA was also evident in humans. Patients were selected and recruited from individuals undergoing laparoscopic bariatric surgery and were divided into three groups based on pathology assessment of liver biopsies: No NAFLD, NAFL (nonalcoholic fatty liver), and NASH. Clinical characteristics are shown in Supplementary Table S4 and liver pathology in Supplementary Table S5. *ARSA* mRNA expression tended to increase with NAFL and was strongly upregulated with NASH (Fig. 2a), effects that were related to steatosis and hepatocyte ballooning, but not inflammation or fibrosis (Supplementary Fig. S2A–D). Similarly, ARSA protein secretion from precision-cut liver slices obtained from patients was increased with NAFL and NASH when compared to patients with no adverse liver pathology (Fig. 2b). ARSA secretion was increased with steatosis (Fig. 2c), but not with inflammation, fibrosis or hepatocyte ballooning (Supplementary Fig. S2E–G), and correlated with plasma ALT, a marker of liver damage (Supplementary Fig. S2H). A sub-analysis showed increased liver ARSA secretion in patients with T2D compared with no diabetes (Fig. 2d). These responses were conserved in mice as ARSA was threefold higher in the plasma of obese insulin-resistant mice compared with lean insulin-sensitive mice (Fig. 2e). Consistent with these findings, ARSA is present in the circulation at concentrations of ~4.5 ng/mL^23^ and is increased in the plasma of individuals with insulin resistance^24^. Assessment of ARSA secretion from isolated tissues of mice, including liver, skeletal muscles, heart, and adipose tissues, showed exceedingly high secretion from the liver, indicating that the liver is a prominent source of circulating ARSA (Fig. 2f). Nevertheless, ARSA release was also observed from skeletal muscle, albeit at 20-fold lower levels than liver (Fig. 2f), pointing to a potential contribution of muscle-derived ARSA to circulating ARSA levels. In addition, ARSA is ubiquitously expressed (according to the genotype-tissue Expression Portal) and we cannot discount the contribution of other tissues to ARSA’s endocrine role. Together, these observations demonstrate that ARSA secretion is increased in NAFLD/NASH, is most closely linked to steatosis, and is associated with insulin resistance.

### Acute ARSA administration improves glycemic control

**Fig. 3:**
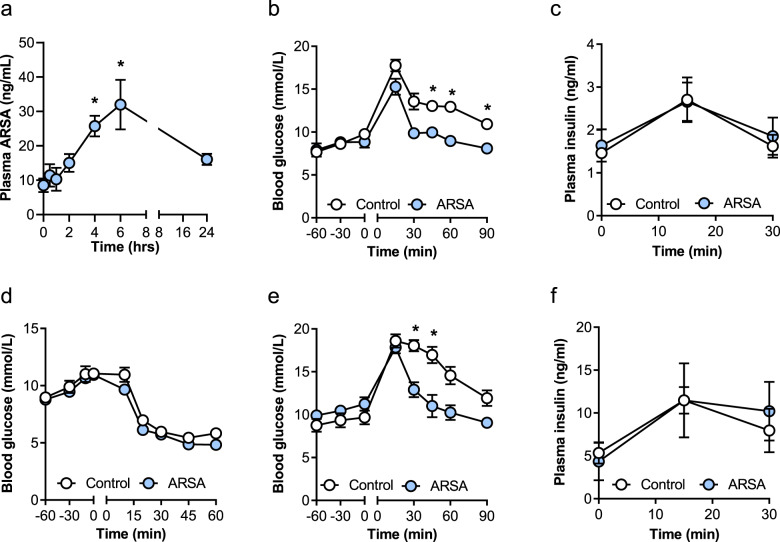
Acute recombinant ARSA administration improves glycemic control. Male mice were injected with recombinant ARSA (1 mg/kg body weight) followed by metabolic assessment an hour later. **a** Plasma ARSA concentration before (0 min) and after recombinant ARSA injection (*n* = 5), *P = 0.0166 0 vs. 4 h, *P = 0.0007 0 vs. 6 h. **b** Oral glucose tolerance test in lean C57BL/6 mice (2 g/kg body weight; *n* = 6/group), *P = 0.0179 at 45 min, *P = 0.0039 at 60 min, *P = 0.0046 at 90 min. **c** Plasma insulin levels during the glucose tolerance test in lean C57BL/6 mice (*n* = 6/group). **d** Insulin-tolerance test (I U/kg body weight, i.p.) in lean C57BL/6 mice. (*n* = 8/group). **e** Oral glucose tolerance test (2 g/kg body weight) in obese C57BL/6 mice fed a high-fat diet for 8 weeks (*n* = 6 Control, *n* = 5 ARSA), *P = 0.0116 at 30 min, *P = 0.0486 at 45 min. **f** Plasma insulin levels during the glucose tolerance test in obese C57BL/6 mice (*n* = 4 Control, *n* = 3 ARSA). For all panels (except panel **a**), white dots = Control, blue dots = ARSA. Data are means ± SEM. *P < 0.05 vs. Control, as assessed by one-way analysis of variance (ANOVA) and Bonferroni post hoc analysis (**a**) or two-way ANOVA with Bonferroni post hoc analysis (**b**–**f**). Source data are provided as a Source Data file.

To investigate the role of ARSA on glycemic control, we first generated recombinant murine protein and confirmed enzymatic activity by its capacity to reduce sulfatides (Supplementary Fig. S3A, B) without affecting ceramide or sphingomyelin levels in isolated skeletal muscle (Supplementary Fig. S3C, D). Recombinant ARSA (1 mg/kg, lowest effective concentration) was injected i.p. into lean mice and 1 h later, blood glucose was assessed following oral glucose administration. ARSA administration increased plasma ARSA levels threefold (Fig. 3a) and improved blood glucose control (Fig. 3b), without affecting basal or glucose-stimulated plasma insulin levels (Fig. 3c). Acute ARSA injection did not affect insulin sensitivity in lean mice (Fig. 3d). ARSA’s effects on glycemic control were recapitulated in obese insulin-resistant mice (Fig. 3e, f), demonstrating a likely endocrine role for ARSA.

### Increasing hepatic ARSA improves glycemic control and muscle insulin action in mice with type 2 diabetes

**Fig. 4:**
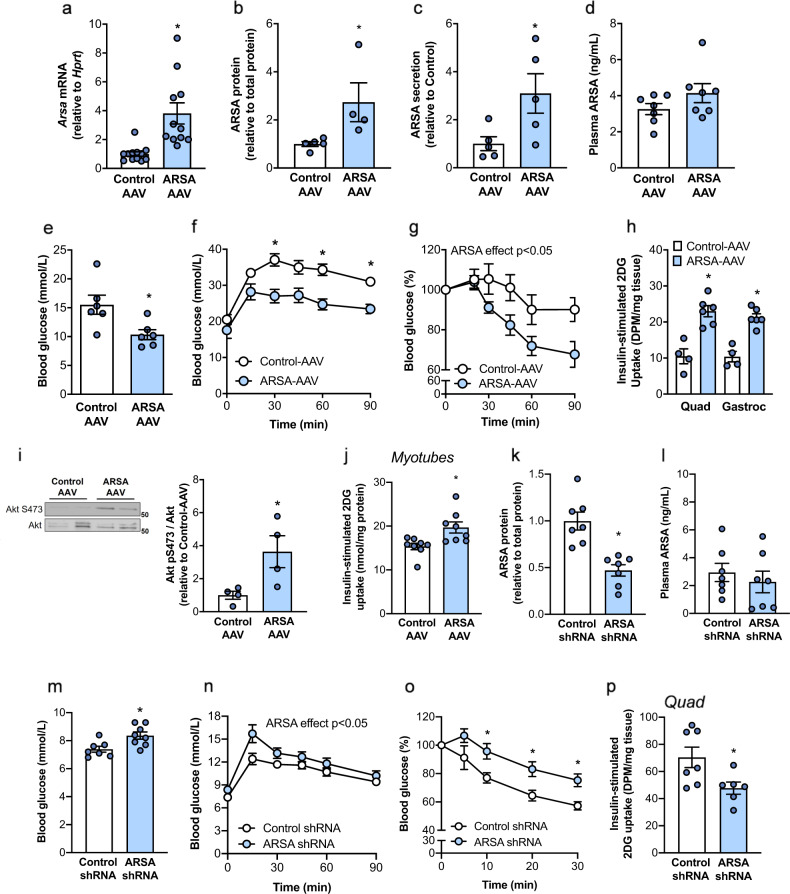
Effects of liver-specific ARSA overexpression and knockdown on glycemic control. ARSA was overexpressed in the livers of db/db mice using a liver-specific adeno-associated virus (AAV; 1 × 10^12^ genome copies/mouse; white bars/line graphs) or Control-AAV (blue bars/line graphs). Metabolic assessments were conducted 8 weeks after AAV administration. **a** Liver *Arsa* mRNA expression (*n* = 12 Control, *n* = 11 ARSA), *P = 0.0008. **b** Liver ARSA protein content (*n* = 5 Control, *n* = 4 ARSA), *P = 0.0467. **c** ARSA secretion from precision-cut liver slices (*n* = 5/group), *P = 0.0431. **d** fasting plasma ARSA (*n* = 7/group). **e** Fasting blood glucose (*n* = 6/group), *P = 0.0196. **f** Oral glucose tolerance test (1 g/kg body weight; *n* = 13 Control, *n* = 15 ARSA), *P = 0.0034 at 30 min, *P = 0.0011 at 60 min, *P = 0.0011 at 90 min. **g** Insulin-tolerance test (2 U/kg body weight, i.p.; *n* = 11/group), ARSA effect *P* = 0.0477. **h** Deoxyglucose uptake (2-DG) into skeletal muscle (*n* = 4/group Control, *n* = 6/group ARSA), assessed 45 min after co-administration of insulin (2 U/kg i.p.) and 10 µCi 1-^14^C-2-deoxyglucose/mouse. Quad mixed quadriceps muscle, Gastroc gastrocnemius muscle. *P = 0.011 Quad, *P = 0.0002 Gastroc. **i** Insulin-stimulated Akt S473 phosphorylation (*n* = 4/group, assessed in quadriceps muscle from experiment (**h**)), *P = 0.038. **j** Medium containing secreted factors from precision-cut liver slices (from experiment (**c**)) was applied to C2C12 myotubes for 24 h, followed by assessment of deoxyglucose (2-DG) uptake (medium was obtained from *n* = 8 independent mice/group), *P = 0.0105. **k**–**p** ARSA was knocked down in hepatocytes of lean C57BL/6 mice using AAV-shRNA (ARSA shRNA) compared with Control shRNA. **k** ARSA protein content in the liver (*n* = 7/group, *P = 0.0006), **l** plasma ARSA (*n* = 7/group) and (**m**) fasting blood glucose (*n* = 7 Control, *n* = 8 ARSA; *P = 0.0132) in ARSA-shRNA and Control-shRNA mice. **n** Oral glucose tolerance test (2 g/kg body weight; *n* = 7 Control, *n* = 8 ARSA), ARSA effect *P = 0.0474. **o** Insulin-tolerance test (1 U/kg body weight, i.p.; *n* = 14 Control, *n* = 16 ARSA), Interaction effect *P = 0.011. **p** Quadriceps muscle deoxyglucose (2-DG) uptake assessed 45 min after co-administration of insulin (2 U/kg i.p.) and 10 µCi 1-^14^C-2-deoxyglucose/mouse (*n* = 7 Control, *n* = 6 ARSA; *P = 0.0315). Data are means ± SEM. *P < 0.05 ARSA-AAV vs. Control-AAV or ARSA shRNA vs. Control shRNA. Data assessed by two-sided unpaired *t* tests (**a**–**e**, **h**–**m**, **p**) or two-way ANOVA and Bonferroni post hoc analysis (**f**, **g**, **n**, **o**), “ARSA effect *P* < 0.05” refers to a significant main effect of treatment. Source data are provided as a Source Data file.

As NAFLD and T2D are closely linked^4^ and as hepatic *ARSA* expression and secretion was increased in mice and humans with NAFLD, we next assessed the metabolic consequences of modulating hepatic ARSA expression in db/db mice, which have NASH and severely impaired glycemic control^25^. ARSA was overexpressed in the livers of obese db/db mice using adeno-associated virus (ARSA-AAV) to recapitulate a state of metabolic disease. Control mice were injected with a liver-targeted AAV containing scrambled cDNA (Control-AAV) and experiments were conducted 8 weeks later. Hepatic overexpression of ARSA was confirmed by RT-qPCR and immunoblot analysis (Fig. 4a, b and Supplementary Fig. S4A), and these effects were confined to the liver (Supplementary Fig. S4A–C). ARSA secretion from precision-cut liver slices was increased threefold (Fig. 4c) and plasma ARSA levels were modestly increased with ARSA-AAV (*P* = 0.14; Fig. 4d), likely reflecting the rapid clearance of the secreted protein. Hepatic ARSA overexpression did not affect body weight (Supplementary Fig. S4D), tissue weights, or plasma lipids (Supplementary Table S6).

Hepatic ARSA overexpression reduced fasting blood glucose levels in mice (Fig. 4e) and improved blood glucose management in response to an oral glucose load (Fig. 4f). These effects were not mediated by changes in plasma insulin levels (Supplementary Fig. S4E). Rather, ARSA overexpression improved whole-body insulin action in mice (Fig. 4g) and further studies using radiolabelled 2-deoxyglucose (Supplementary Fig. S4F) showed that ARSA-AAV increased insulin-mediated glucose disposal into skeletal muscle in vivo (Fig. 4h), but not in other tissues, including heart, kidney, or adipose tissues (Supplementary Fig. S4G, H). The improvement in skeletal muscle insulin sensitivity in ARSA-AAV mice was confirmed by increased phosphorylation of Akt at S473 upon insulin stimulation (Fig. 4i).

To test whether skeletal muscle insulin sensitivity was improved via an endocrine signal from the liver, we collected secreted factors from liver slices of ARSA-AAV and Control-AAV mice and applied these to differentiated C2C12 myotubes. Insulin-stimulated glucose uptake into myotubes was increased when exposed to ARSA-AAV liver-secreted factors compared with Control-AAV (Fig. 4j). Hepatic ARSA overexpression did not directly impact gluconeogenic gene expression (Supplementary Fig. S4I) or glucose output (Supplementary Fig. S4J) in liver slices, highlighting the likelihood that the improved glycemic control with ARSA overexpression occurred via liver–muscle crosstalk.

### ARSA knockdown in the liver impairs glycemic control and insulin action in mice

We next investigated the dependency for liver ARSA in regulating glycemic control in lean mice by AAV-shRNA mediated gene silencing of *Arsa* (ARSA shRNA). Liver ARSA mRNA was reduced by 34% (Supplementary Fig. S4K), without changes in ARSA expression in the skeletal muscle, heart, adipose tissue, or kidney (Supplementary Fig. S4L). This was accompanied by a 50% reduction in ARSA protein content in the liver (Fig. 4k and Supplementary Fig. S4M). Of note, the mild reduction in gene and protein expression of ARSA is likely related to the analysis of whole livers, where non-parenchymal cell populations, which contribute up to 40% of the total number of liver cells^26^, are not targeted by the ARSA shRNA, thereby diluting apparent treatment effects in hepatocytes. Although plasma ARSA was not significantly reduced (25% reduction, Fig. 4l), hepatic knockdown significantly increased fasting blood glucose (Fig. 4m) and worsened glucose tolerance (Fig. 4n), whole-body insulin sensitivity (Fig. 4o), and insulin-stimulated glucose uptake into skeletal muscle (Fig. 4p). ARSA shRNA did not impact fasting or glucose-stimulated plasma insulin levels (Supplementary Fig. S4N), glucose uptake into other tissues (Supplementary Fig. S4O), body weight (Supplementary Fig. S4P), adiposity (Supplementary Fig. S4Q), oxygen consumption (Supplementary Fig. S4R), systemic fat or carbohydrate oxidation (Supplementary Fig. S4S), food intake (Supplementary Fig. S4T) or plasma NEFA, triglycerides and cholesterol levels (Supplementary Table S7). Overall, these experiments using both loss and gain-of-function approaches indicate that liver ARSA impacts systemic glycemic control by improving skeletal muscle insulin action.

### Liver ARSA does not directly modulate skeletal muscle lipid raft composition or insulin action

**Fig. 5:**
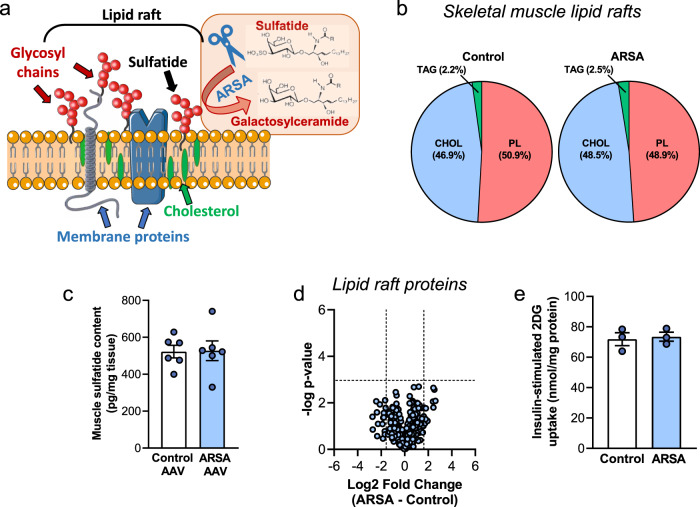
Liver-derived ARSA does not modulate skeletal muscle lipid rafts. **a** ARSA degrades sulfatides that are primarily localized to cell surface lipid rafts, which are microdomains enriched in cholesterol, sphingolipids, and membrane proteins. **b** Skeletal muscle lipid raft composition, shown as a percentage of the major lipid classes (average of *n* = 6/group). CHOL cholesterol, PL phospholipid, TAG triglyceride. **c** Skeletal muscle sulfatide content in Control-AAV (white bar) and ARSA-AAV (blue bar) mice (*n* = 6/group). **d** Volcano plot showing no difference in the proteins contained in skeletal muscle lipid rafts derived from Control-AAV and ARSA-AAV mice. **e** 1-^14^C-2-deoxyglucose (2-DG) uptake into differentiated C2C12 myotubes following 24 h incubation without (white bar) or with ARSA (10 ng/mL, blue bar) (*n* = 3/group, from three individual experiments). Data are means ± SEM. Data assessed using two-tailed unpaired *t* tests (**c**, **e**). Source data are provided as a Source Data file.

Having identified phenotypic effects associated with ARSA, we next aimed to determine how liver-derived ARSA improves skeletal muscle insulin action, focussing on ARSA’s primary known function, which is the breakdown of sulfatide lipids localized primarily to lipid rafts on the cell surface^27^ (Fig. 5a). Skeletal muscle lipid rafts were isolated using sucrose gradient centrifugation^28^ (Supplementary Fig. S5A) and purity was confirmed by enrichment of the lipid raft protein Thy1/CD90 and absence of proteins enriched in other organelles (Supplementary Fig. S5B). Similar to a previous report^29^, lipid rafts contained on average 47% cholesterol and 50% phospholipids, in addition to sphingolipids, triacylglycerols, diacylglycerols, and lysophospholipids (Fig. 5b). Unexpectedly, muscle sulfatide content was not different between ARSA-AAV and Control-AAV mice (Fig. 5c). Lipidomic analysis revealed very few differences in the lipid composition of skeletal muscle lipid rafts between groups with negligible decreases in cholesterol, phospholipids (PI, PG) and LPI in ARSA-AAV compared to Control-AAV (Supplementary Fig. S5C–J). While the content and spatial organization of proteins, including those involved in insulin signal transduction, can be altered by changes in the lipid raft lipid composition^30^, unbiased MS/MS analysis revealed no significant differences in the lipid raft proteome between treatment groups (Fig. 5d and Supplementary Fig. S5K–L). Further experiments in differentiated C2C12 myotubes showed that recombinant murine ARSA does not directly affect insulin-stimulated glucose uptake (Fig. 5e). These data demonstrate that ARSAs insulin-sensitizing effects in muscle are not mediated by changes in lipid raft composition, and importantly, are not mediated by direct actions of ARSA on muscle.

### Liver ARSA stimulates LPC and LPA accumulation in liver lipid rafts

**Fig. 6:**
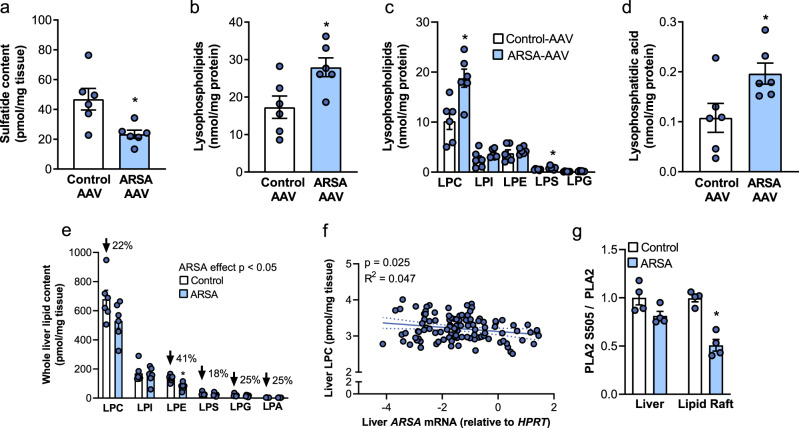
Hepatic ARSA remodels lipid rafts in the liver. ARSA was overexpressed in the livers of male db/db mice using a liver-specific adeno-associated virus (ARSA-AAV, blue bars) and was compared with Control-AAV (white bars). **a** Sulfatide content in whole-liver extracts (*n* = 6/group), *P = 0.0130. **b** Lysophospholipid content (*P = 0.0209), **c** lysophospholipid classes (*P = 0.006 for LPC, *P = 0.0241 for LPS), and **d** lysophosphatidic acid in liver lipid rafts (*n* = 6/group, *P = 0.033). **e** Lysophospholipid classes in whole-liver extracts (*n* = 6/group). ARSA effect *P = 0.0122, *P = 0.002 for LPE. **f** Correlation of *ARSA* mRNA expression and lysophosphatidylcholine (LPC) content in human livers (*n* = 106). **g** Phospholipase A2 (PLA2) S505 phosphorylation in whole-liver and liver lipid rafts (*n* = 4/group, *P = 0.0001 for LR). Data are means ± SEM. *P < 0.05 vs. Control-AAV, as assessed by two-tailed unpaired *t* tests or linear regression analysis (**f**). LPA lysophosphatidic acid, LPC lysophosphatidylcholine, LPE lysophosphatidyl ethanolamine, LPG lysophosphatidyl glycerol, LPI lysophosphatidyl inositol, LPS lysophosphatidyl serine, PLA2 phospholipase A2. Source data are provided as a Source Data file.

Given that the liver has a remarkable capacity for ARSA uptake^31^ and that ARSA acts on sulfatides primarily localized to lipid rafts^27^, we reasoned that ARSA might regulate lipid metabolism on the cell surface of the liver in an autocrine/paracrine manner, which may subsequently regulate skeletal muscle insulin action via release of lipid metabolites into the circulation. Lipidomic analysis confirmed a significant reduction in sulfatide levels in ARSA-AAV compared with Control-AAV livers (Fig. 6a). Lipid rafts isolated from ARSA-AAV livers (Supplementary Fig. S6A) were also enriched in lysophospholipids compared with Control-AAV mice (Fig. 6b). This was most prominent for lysophosphatidylcholine (LPC) (Fig. 6c), which was accompanied by a significant increase in lysophosphatidic acid (LPA) (Fig. 6d), a product of LPC breakdown. LPA accumulation within lipid rafts was not associated with increased activity of lipid raft-localized autotaxin, a phospholipase enzyme that catalyzes the conversion of lysophospholipids to LPA (Supplementary Fig. S6B). In contrast, lipid raft phospholipid composition was not impacted by hepatic ARSA (Supplementary Fig. S6C, D), while lipid raft cholesterol tended to be reduced (*P* = 0.22, Supplementary Fig. S6E). The changes in lysophospholipids were specific to lipid rafts as total lysophospholipid (Supplementary Fig. S6F) and several lysophospholipid classes, including LPC and LPA, were actually reduced in whole livers of ARSA-AAV mice (Fig. 6e), while liver phospholipid and cholesterol composition was unchanged (Supplementary Fig. S6G, H). Examination of human livers showed a modest but significant negative correlation between hepatic *ARSA* expression and LPC content (Fig. 6f), suggestive of ARSA-mediated regulation of hepatic lysophospholipid metabolism.

The changes in lipid raft lysophospholipid composition were not readily explained by known regulators of lysophospholipid metabolism including phospholipase A2 activation or the expression of proteins that directly or indirectly regulate lysophospholipid synthesis (i.e., LPC acyltransferases) or degradation (i.e., lysophospholipase 2, choline-phosphate cytidylyltransferase A, glycerol-3-phosphate acyltransferases, or ectonucleotide pyrophosphatase/phosphodiesterase 2, i.e., autotaxin) (Fig. 6g and Supplementary Fig. S6I–K).

### Hepatic ARSA reduces LPC and LPA secretion from the liver and regulates plasma LPA and skeletal muscle insulin action

**Fig. 7:**
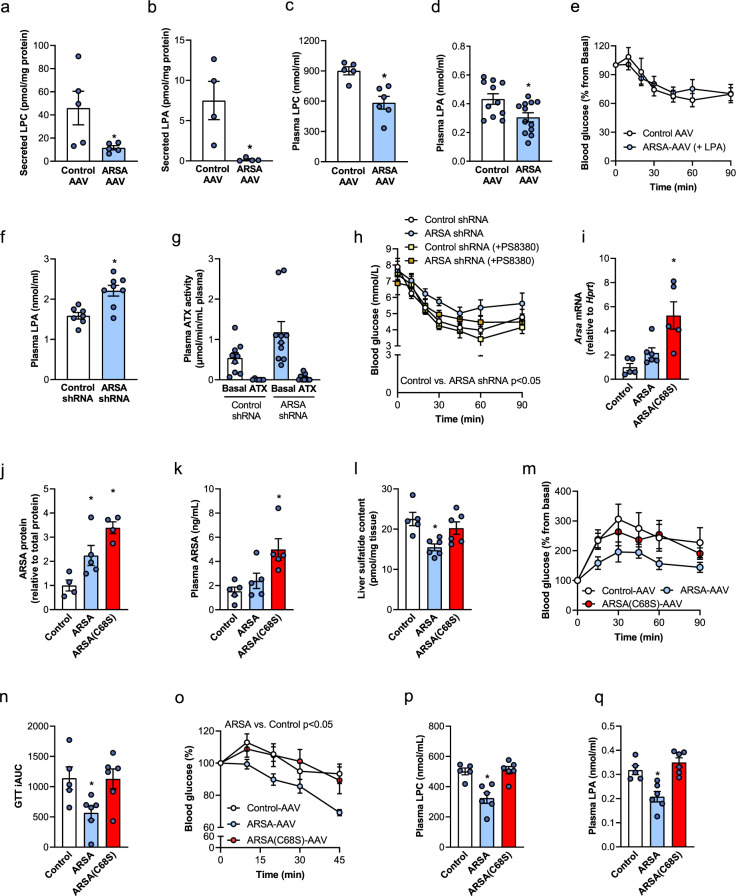
ARSA’s capacity for sulfatide degradation is required for its effects on hepatic LPC and LPA secretion, and glycemic control. **a** Lysophosphatidylcholine (LPC) (*n* = 5/group, *P = 0.0465) and **b** lysophosphatidic acid (LPA) (*n* = 4/group, *P = 0.0213) secretion from liver slices of Control-AAV (white bars) and ARSA-AAV (blue bars) mice. **c** Plasma LPC (*n* = 5 Control, *n* = 6 ARSA, *P = 0.0031) and **d** LPA in Control-AAV (white bars) and ARSA-AAV (white bars) mice (*n* = 11 Control, *n* = 12 ARSA, *P = 0.0158). **e** ARSA-AAV mice were injected with 200 ng LPA (i.p.) 30 min before ITT (2 U/kg body weight). Control-AAV mice received an i.p. saline injection (*n* = 4/group). **f** Plasma LPA in mice with hepatic silencing of ARSA, with Control shRNA (white bars) vs. ARSA shRNA-AAV (blue bars) (*n* = 7 Control, *n* = 8 ARSA, *P = 0.0019). **g** Plasma autotaxin activity (*n* = 9 Control PEG400, *n* = 6 Control PS8380, *n* = 10 ARSA PEG400, *n* = 9 ARSA PS8380) and **h** insulin sensitivity (*n* = 8 Control, *n* = 9 ARSA, *n* = 5 Control+PS8380, *n* = 9 ARSA + PS8380; *P = 0.0144 Control vs. ARSA shRNA) in ARSA shRNA (blue bars) or Control-shRNA (white bars) mice, in the absence (circles) or presence (squares) of acute plasma autotaxin inhibition with PF8380, dosed orally at 30 mg/kg 3 h before the ITT assessment. Of note, ITT data for Control shRNA and ARSA shRNA also form part of Fig. 4O. **i**–**q** ARSA (ARSA-AAV, blue bars/line graphs) or enzymatically inactive ARSA (ARSA(C68S)-AAV, red bars/line graphs) was overexpressed in the livers of db/db mice. **i** Liver *Arsa* mRNA expression (*n* = 5/group Control and ARSA(C68S), *n* = 6 ARSA; *P = 0.055 Control vs. ARSA, *P = 0.0225 Control vs. ARSA(C68S)) and **j** ARSA protein content (*n* = 4/group Control and ARSA(C68S), *n* = 5 ARSA; *P = 0.046 Control vs. ARSA, *P = 0.0004 Control vs. ARSA (C68S)). **k** Plasma ARSA levels (*n* = 5/group, *P = 0.0066 Control vs. ARSA(C68S)). **l** Sulfatide content in whole liver (n = 5 Control, *n* = 6/group ARSA and ARSA(C68S)), *P = 0.0032 Control vs. ARSA. **m**, **n** Oral glucose tolerance test (1 g/kg body weight) and respective incremental area under the curve (*n* = 5 Control, *n* = 6 ARSA, *n* = 6 ARSA(C68)), *P = 0.0254 Control vs. ARSA. **o** Insulin-tolerance test (2 U/kg body weight) (*n* = 5 Control, *n* = 5 ARSA, *n* = 6 ARSA(C68)), *P = 0.0177 Control vs. ARSA. **p** Plasma LPC (*P = 0.0031 Control vs. ARSA) and **q** plasma LPA levels (*P = 0.0048 Control vs. ARSA) (*n* = 5 Control, *n* = 6 ARSA, *n* = 6 ARSA(C68)). Data are means ± SEM, *P < 0.05 vs. Control-AAV, as assessed by two-tailed unpaired *t* tests (**a**–**d**, **f**, **g**), one-way analysis of variance (ANOVA) and Bonferroni post hoc analysis (**i**–**l**, **n**, **p**, **q**) or two-way ANOVA and Bonferroni post hoc analysis (**e**, **h**, **m**, **o**), where main effects for treatment are reported. Source data are provided as a Source Data file. ATX autotaxin, AUC area under curve, GTT glucose tolerance test, LPA lysophosphatidic acid, LPC lysophosphatidylcholine.

LPC is secreted from the liver at greater rates than any other phospholipid^32^, and LPC is the second most abundant phospholipid found in the circulation^33^. LPC can be directly secreted from the liver and primarily circulates bound to albumin^33,34^. Therefore, we next asked whether the accumulation of lipid raft LPC and LPA with ARSA overexpression was related to reduced secretion of these lipids from the liver. Studies using precision-cut liver slices demonstrated a 70% reduction in LPC secretion (Fig. 7a) and a 97% reduction in LPA secretion (Fig. 7b) with ARSA-AAV compared to Control-AAV, while secretion of other lysophospholipids and phospholipids (Supplementary Fig. S7A, B), as well as secretion of autotaxin (Supplementary Fig. S7C), was unaffected by ARSA overexpression.

Autotaxin rapidly converts circulating LPC to LPA, which signals through six GPCRs (LPA-R1-6) to contribute to obesity-induced insulin resistance in skeletal muscle^35^. Consistent with reduced liver LPC and LPA release (Fig. 7a, b), ARSA overexpression reduced plasma LPC and LPA levels in mice (Fig. 7c, d). Plasma autotaxin activity was similar between groups (Supplementary Fig. S7D), indicating that the reduced plasma LPA was most likely mediated by reduced secretion from the liver, rather than conversion of plasma LPC to LPA. The plasma concentrations of other lipids, aside from cholesterol, were not different between ARSA-AAV and Control-AAV mice, demonstrating the specificity of this response (Supplementary Fig. S7E–J).

To test whether reductions in circulating LPA contribute to insulin sensitization in the skeletal muscle with ARSA overexpression, we restored plasma LPA levels in ARSA-AAV mice to those of Control-AAV mice by intraperitoneal injection (Supplementary Fig. S7K). While ARSA-AAV improved insulin sensitivity under normal conditions (Fig. 4g), insulin tolerance was indistinguishable between Control-AAV mice and ARSA-AAV mice injected with LPA (Fig. 7e). The importance of plasma LPA in ARSA-mediated liver–muscle communication is further supported by the observation that hepatic ARSA knockdown (Fig. 4m–o) reduced insulin sensitivity and muscle insulin action in mice, with concomitant increases in plasma LPA (Fig. 7f) and no changes in plasma autotaxin activity (Supplementary Fig. S7L).

As restoration of plasma LPA negated the improvements in insulin sensitivity in ARSA-AAV mice (Fig. 7e), we next tested whether reducing plasma LPA in ARSA-shRNA mice would improve insulin sensitivity. This was achieved by performing insulin-tolerance tests in ARSA-shRNA and Control-shRNA mice in the absence or presence of the autotaxin inhibitor PF8380, which reduces plasma LPA levels by 95% (Fig. 7g)^36^. Consistent with our earlier studies (Fig. 4o), insulin sensitivity was impaired in ARSA-shRNA compared with Control-shRNA mice. Treatment with PF8380 improved insulin tolerance in ARSA-shRNA mice such that their blood glucose response was indistinguishable from Control-shRNA (Fig. 7h). Together, these studies demonstrate that lower circulating LPA levels improve muscle insulin sensitivity with hepatic ARSA overexpression.

### ARSA effects are dependent on enzymatic activity toward sulfatide

To determine whether the ARSA-mediated effects on glucose tolerance and insulin sensitivity are dependent on ARSA’s capacity to breakdown sulfatides, we generated an AAV containing a sequence for an enzymatically inactive ARSA mutant (C68S)^31^. Overexpression of ARSA and ARSA(C68S) increased liver *ARSA* mRNA (Fig. 7i) and protein (Fig. 7j and Supplementary Fig. S7M), without changes in other tissues (Supplementary Fig. S7N). This resulted in a 60% increase in plasma ARSA in ARSA-AAV mice and a 330% increase in ARSA(C68S)-AAV mice (Fig. 7k). The difference in ARSA expression and plasma levels between groups likely reflects differences in the transduction efficiency despite delivering equal titer. ARSA overexpression was accompanied by a significant reduction in liver sulfatide content, while the ARSA(C68S) mutant did not affect liver sulfatides (Fig. 7l), as expected. Glucose tolerance (Fig. 7m, n) and insulin sensitivity (Fig. 7o) were significantly improved in ARSA-AAV mice compared with Control-AAV, whereas overexpression of the ARSA(C68S) mutant did not impact glycemic control or insulin sensitivity (Fig. 7m–o). Importantly, while ARSA overexpression reduced plasma LPC and LPA, these changes were absent in ARSA(C68S) AAV mice (Fig. 7p, q). These experiments link enzymatically active hepatic ARSA to improved insulin action and glycemic control via reduced circulating LPA levels.

## Discussion

**Fig. 8:**
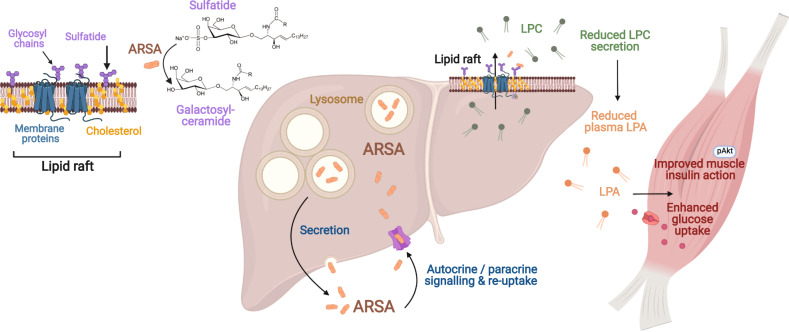
Graphic summary highlighting the impact of liver-derived ARSA on sulfatide and lysophospholipid metabolism in liver lipid rafts, the reduction in LPC and LPA levels in the blood, and how the reduced LPA relieves skeletal muscle insulin resistance. LPA lysophosphatidic acid, LPC lysophosphatidylcholine. Created with BioRender.com.

NASH is closely linked to prevalent metabolic diseases including obesity and type 2 diabetes;^1,4^ however, the extent to which liver-derived circulating factors such as proteins, lipids, and non-coding RNAs contribute to metabolic comorbidities in NASH is unresolved. We have developed a unique resource describing global changes in hepatokine secretion in murine NASH, which provides a deeper appreciation of potential endocrine circuits in NASH and identification of putative therapeutic targets for related comorbidities, in particular impaired glycemic control. Using this system, ARSA was identified as a liver-secreted protein that is increased in NAFLD/NASH and type 2 diabetes in humans. ARSA modulated hepatic sulfatide and lysophospholipid metabolism and reduced LPC and LPA secretion from lipid rafts, thereby improving skeletal muscle insulin sensitivity and whole-body glycemic control (Fig. 8).

The identification of hepatocyte-secreted molecules is challenging and requires an experimental model that captures cell-intrinsic characteristics without interference from nutrient fluxes and circulating factors such as hormones, cytokines, and extracellular vesicles. Our discovery pipeline addressed these requirements by collecting secreted factors from isolated hepatocytes derived from independent murine models of NASH and identifying secreted proteins using mass spectrometry. This contrasts with previous studies that indirectly assessed hepatokine secretion in NASH using bioinformatic approaches to delineate the hepatocyte secretome from gene expression data^15^ or from cross-sectional studies correlating changes in plasma proteins with NAFLD prevalence and/or progression^37–39^. Our data add scientific knowledge by describing endocrine networks that are likely to be dysregulated in NASH and that contribute to inflammation, apoptosis, and cancer. Bioinformatic analysis predicted alterations in secreted proteins that impact various aspects of lipid metabolism (e.g., fatty acid metabolism, lipid synthesis, lipid export), and our own experiments in cultured cells support the likelihood that NASH-induced hepatokines contribute to altered systemic lipid metabolism and insulin action (Fig. 1). These findings align with recent studies examining NASH-induced changes in the intracellular transcriptome and proteome of hepatocytes and/or liver biopsies in mouse and human NASH that predict reduced lipid metabolism, increased inflammatory response, and extracellular matrix remodeling^40–44^. Taken together, this growing body of evidence highlights an endocrine link between NASH and its comorbidities, including dyslipidemia and type 2 diabetes.

This study identified ARSA as a liver-secreted protein that improves skeletal muscle insulin sensitivity and glycemic control. Our study shows that increasing liver ARSA expression and secretion via adeno-associated virus delivery was sufficient to improve whole-body glycemic control in obesity, which was mediated by improvements in skeletal muscle insulin action. Consistent with these findings, gene silencing of *Arsa* in livers of lean mice was sufficient to impair whole-body insulin action secondary to reduced insulin-stimulated glucose disposal in skeletal muscle. Moreover, a single injection of recombinant ARSA protein improved glycemic control in both lean and obese mice, suggesting that the phenotypic changes could be attributed to ARSAs endocrine role. However, treating cultured myotubes with recombinant ARSA did not affect insulin sensitivity, raising the possibility that another factor related to ARSA overexpression induced these changes, a premise supported by (i) the finding in cultured myotubes that factors secreted from the liver of ARSA overexpressing mice improved insulin sensitivity compared with factors secreted by wild-type mice, and (ii) the finding that hepatic ARSA knockdown worsened glucose tolerance and insulin sensitivity despite not impacting plasma ARSA levels (Fig. 4).

ARSA is predominantly localized to lysosomes and is required for the degradation of sulfatide sphingolipids^9^. ARSA degrades sulfatides and remodels the lipid composition of cell surface lipid rafts in the liver, specifically resulting in reduced sulfatide content and selective accumulation of LPC and LPA. These changes were not conserved throughout the liver. Liver ARSA secretion is 15–500 times greater than secretion from other tissues, and plasma ARSA is primarily targeted to the liver^10^, indicating that liver-secreted ARSA is most likely accompanied by rapid re-uptake via the mannose-6-phosphate receptor^13^, which is well documented to mediate endocytosis of the majority of lysosomal proteins containing mannose-6-phosphate residues^45^, including ARSA. This supports the possibility that ARSA induces its effects through several pathways: (i) secreted ARSA is rapidly transported back into hepatocytes where it induces its action at lipid rafts, perhaps via a small population of lysosomes localized to the plasma membrane^46^, and/or (ii) secreted ARSA removes the sulfate group on the extracellular surface of lipid raft sulfatides.

While the precise mechanisms underpinning LPC and LPA accumulation in lipid rafts remain unclear, we show that the enzymatic activity of ARSA is required for this effect, and for the reduction in liver LPC and LPA secretion and associated decrease in plasma LPC and LPA levels. Circulating LPC is a substrate of the extracellular protein autotaxin, which hydrolyzes LPC to LPA^47^. LPA causes insulin resistance in hepatocytes^48^ and myotubes^35^, and is positively associated with obesity and whole-body insulin resistance^48–50^. Importantly, the present study found that circulating LPA was reduced with hepatic ARSA overexpression and was increased with ARSA knockdown, concomitant with muscle insulin sensitization and resistance, respectively. Moreover, the insulin-sensitizing effect of hepatic ARSA overexpression was completely abrogated after a one-off LPA injection that restored plasma LPA to levels of wild-type controls, while reducing plasma LPA by autotaxin inhibition restored insulin action in mice with hepatic ARSA knockdown. Altogether, these findings strongly point towards the existence of ARSA-mediated regulation of hepatic LPC and LPA secretion in driving a previously unappreciated liver–muscle endocrine axis in the regulation of insulin action and systemic glycemic control.

While multiple lines of evidence provide compelling support for this conclusion, there was an intriguing disconnect following acute i.p. administration of recombinant ARSA, where glucose tolerance was improved independently of changes in plasma LPA or insulin sensitivity. We surmise that i.p. administration of recombinant ARSA is insufficient to increase local concentrations of liver ARSA required to impact liver LPA metabolism (Supplementary Fig. S7O), indicating that other ARSA-mediated effects impact glycemic control. Such possibilities might include the regulation of glucose effectiveness, modulation of other unidentified hormones or metabolites, and central nervous system effects.

ARSA is increased in NASH and type 2 diabetes, in both mice and humans. This result is intriguing, and on face value paradoxical, given that NASH is associated with insulin resistance and type 2 diabetes^4^ and that the secreted factors from NASH hepatocytes are collectively insulin resistance inducing (Fig. 1). However, the concentrations of ARSA attained under pathological conditions might be insufficient to drive a therapeutic benefit, especially in obesity where multiple factors induce insulin resistance. In addition, emerging literature shows that not all factors secreted at increasing rates in NAFLD are detrimental for local or systemic metabolism, and include FGF-21^51,52^, SMOC1^25^, and activin E^53,54^. Exploration of the NASH-inducible proteins detected in this study may identify further therapeutic targets for conditions characterized by insulin resistance.

In summary, we have provided a resource describing the changes in hepatokine secretion in NASH and a deeper appreciation of potential endocrine circuits that mediate NASH comorbidities such as defective lipid metabolism and insulin action. Further, we identified ARSA as a NAFLD/NASH-induced hepatokine that regulates hepatic and systemic lysophospholipid metabolism, and as powerful modulator of skeletal muscle insulin action. ARSA protein and gene therapy has been shown to be a safe therapeutic approach for metachromatic leukodystrophy in humans^55–57^, and future studies are warranted to assess the potential for ARSA as a therapeutic strategy for the treatment of type 2 diabetes.

## Methods

### Human studies

#### 1. Assessment of hepatic ARSA gene expression

Ethics approval was obtained from relevant Human Research Ethics Committees (Alfred (195/15), Avenue (190) and Cabrini (09-31-08-15)) and the study was registered with the Australian Clinical Trials Register (ACTRN12615000875505: Non-invasive diagnosis and monitoring of nonalcoholic fatty liver disease in bariatric surgical patients). The primary outcomes of this study were the efficacy of transient elastography, magnetic resonance spectroscopy (MRS) and serum biomarkers in measuring NAFLD in a bariatric cohort by comparison with liver biopsy. The secondary outcomes were a change in NAFLD with surgically induced weight loss as measured by liver biopsy, and the correlation of mRNA profile of adipose tissue with liver biopsy in patients with NAFLD. We prospectively enrolled all eligible patients with obesity undergoing bariatric surgery, who fit criteria for likely NAFLD, including AST or ALT > 0.5 upper limit normal, GGT > upper limit normal, abnormal transient elastography, and/or abnormal ultrasound suggesting NAFLD. All enrolled patients underwent a MRS scan (at baseline and 1 year after bariatric surgery), a transient elastography/Fibroscan (at baseline, and 3 and 12 months after bariatric surgery), and bloods were taken for routine blood tests (at baseline and 1, 3, and 12 months after bariatric surgery). A liver core biopsy, as well as a small wedge biopsy, were taken during the bariatric procedure (at baseline), amounting to a total of less than 1 cm^3^ of liver tissue. In addition, a piece of visceral and subcutaneous fat was taken during the operation from the omentum around the area of surgery. For patients with >33% steatosis, any inflammation or any fibrosis were offered a follow-up percutaneous liver biopsy at 12 months. Of note, only liver samples collected at baseline were used in this study. The outcomes reported here are unrelated to the primary and secondary outcomes of ACTRN12615000875505.

Participants provided written informed consent, in the absence of any form of compensation. Data were collected at the Avenue or Cabrini Hospital (Melbourne, Australia), and stored at the Center of Obesity Research and Education (CORE) at the Alfred Centre (Melbourne, Australia). Recruitment started 06-2015 to 12-2017. The study design, patient characteristics, inclusion/exclusion criteria, and histopathological grading have been published previously^58,59^.

#### 2. Assessment of hepatic ARSA secretion

Ethics was obtained from the University of Melbourne Human Ethics Committee (ethics ID 1851533) and approved by The Avenue Hospital Human Research Ethics Committee (ethics ID WD00006, HREC reference number 249) and the Alfred Hospital Human Research Ethics Committee (ethics ID GO00005). This study has been approved as a “Biospecimen analysis research” and is not considered “Interventional/Clinical Trial research”, thereby not requiring a clinical trial registration. Data were collected at the Avenue or Cabrini Hospital (Melbourne, Australia), and stored at the Center of Obesity Research and Education (CORE) at the Alfred Centre (Melbourne, Australia). Recruitment started 06-2017 to 08-2019. Medical history was taken from the patient and the patient’s medical chart. Data was also taken from Melbourne Pathology and TissuePath reports. The primary outcome measure in these studies was the discovery of novel non-invasive biomarkers for NAFLD and NASH.

For liver secretion studies, bariatric patients with likely NAFLD were recruited, including the previous history of NAFLD/NASH and presence of metabolic comorbidities (e.g., type 2 diabetes, hypertension, obstructive sleep apnea). Exclusion criteria included: age <18 years; current or past excessive ethanol use (>210 g/week males, >140 g/week females); other causes of chronic liver disease and/or hepatic steatosis including Wilson’s disease, α-1-antitrypsin deficiency; viral hepatitis; primary biliary cirrhosis; autoimmune hepatitis; genetic iron overload; hypo- or hyperthyroidism; coeliac disease; hepatitis B/C; human immunodeficiency virus; recent (within 3 months of screening visit) or concomitant use of agents known to cause hepatic steatosis including corticosteroids, amiodarone, methotrexate, tamoxifen, valproic acid, and high-dose estrogens. Pre-operative clinical details were collected using a questionnaire, including metabolic comorbidities, screening for alternate causes for liver disease (as detailed above in exclusion criteria), medications, social and family history. Patients fasted overnight and blood tests were taken prior to surgery for assessment of plasma ALT and AST. In this study, we recruited 121 patients (28% male), with an average age of 47 years. Participants provided written informed consent, in the absence of any form of compensation.

For both ARSA gene expression and hepatic ARSA secretion studies, a 1-cm^3^ wedge biopsy was collected from the left lobe of the liver. A portion of the liver was fixed in 10% formalin, processed, and stained with hematoxylin and eosin or Masson’s Trichrome for histopathological analysis. Liver sections were graded by a pathologist in a blinded manner, according to the Clinical Research Network (CRN) NAFLD activity score (NAS)^60^ and Kleiner classification of liver fibrosis^61^. Patients were stratified according to hepatic scores for steatosis (5% to 33% of parenchyma is involved for grade 1, >33% to 66% for grade 2, and >66% for grade 3), inflammation (<2 foci are present per ×200 field for grade 1, 2, to 4 foci for grade 2, and >4 foci for grade 3), hepatocyte ballooning (few or many ballooning cells are present per high-power field for grade 1 or 2, respectively) and fibrosis (grading according to Kleiner^61^). NASH was determined as the joint presence of steatosis, ballooning, and lobular inflammation (NAFLD activity score ≥3) as defined by the Clinical Practice Guidelines of the European Association for the Study of the Liver (EASL), the European Association for the Study of Diabetes (EASD) and European Association for the Study of Obesity (EASO)^62^, NAFLD was determined as presence of 1–2 of the above variables, and No NAFLD as the absence of steatosis, inflammation, hepatocyte ballooning, and fibrosis.

For hepatic ARSA secretion studies, the remainder of the liver wedge biopsy was embedded in 3% agarose (SeaPlaque™ Agarose, Lonza Bioscience) and 300-µm-thick liver slices were generated using a Krumdieck Tissue Slicer (TSE Systems China Ltd). Liver slices were maintained in oxygenated M199 media (Thermo Fisher Scientific, Scoresby, Australia) for 60 min before collection of secreted factors in oxygenated protein-free EX-CELL^®^ 325 PF CHO Serum-Free medium (Sigma-Aldrich, Castle Hill, Australia) for 16 h. This medium was used for mass spectrometry proteomics analysis.

### Animal experimental procedures

All mouse experiments were approved by the Monash University Animal Ethics Committee (MARP/2016/073) and the University of Melbourne Anatomy & Neuroscience, Pathology, Pharmacology, and Physiology Animal Ethics Committee (ethics ID 1814403), and conformed to the National Health and Medical Research Council of Australia guidelines regarding the care and use of experimental animals. Mice used in this study were either male 8–10 week old C57BL/6 (sourced from Monash University MARP breeding facility, or Animal Resources Centre, Canning Vale, Australia) or male db/db mice (Jackson Laboratory strain: BKS.Cg-Dock7m + /+ Leprdb/J). All mice were maintained in a temperature-controlled room (22 °C ± 1 °C) with a 12-hour light/dark cycle and 40–60% humidity, and had ad libitum access to food and water.

For induction of NASH, C57BL/6 mice were fed one of two diets. A methionine and choline-deficient (MCD) diet (SF16-035; Specialty Feeds, Australia) consisting of 44% calories from fat (lard) and low methionine (1 g/kg) and choline (70 mg/kg) content. Control mice in this study group received the same dietary composition, however with higher levels of methionine (6.1 g/kg) and choline (1850 mg/kg) (SF16-034; Specialty Feeds, Australia). Mice were fed either the control or the MCD diet for 12 weeks. The second NASH diet was a high-fat (40% calories from lard), high-fructose (30% calories from fructose) diet supplemented with 2% cholesterol (20 g/kg) (SF16-033; Specialty Feeds, Australia), from herein referred to as CHOL diet. Control mice in this study group received standard rodent chow (5% of energy from fat, Specialty Feeds, WA, Australia). Mice were fed either diet for 40 weeks.

For recombinant protein studies, mice were fed either a standard low-fat laboratory diet (5% of energy from fat, Specialty Feeds, WA, Australia) or a high-fat, high-sucrose diet (HFD, high-fat rodent diet SF04-001, 43% energy from fat, Specialty Feeds, Australia) starting at 8 weeks of age for a total of 8–12 weeks.

Plasma and tissue samples were collected from animals fasted for 4 h, unless specified otherwise. For validation of the presence of NASH, a piece of liver was fixed in formalin, embedded in paraffin, sections stained with hematoxylin and eosin (H&E) or Masson’s Trichrome (Monash University Histology Platform, Melbourne, Australia) and scored (as above for human studies) by an experienced histopathologist.

### Hepatocyte isolations and collection of conditioned media

Primary murine hepatocytes were isolated by collagenase perfusion^63^. Briefly, the liver was perfused through the inferior vena cava with EGTA buffer (HBSS buffer + 0.5 mmol/l EGTA) for 15 min, followed by collagenase digestion (50 mg Collagenase H, Roche) in calcium buffer (HBSS buffer + 2 mmol/l CaCl_2_) for 9 min. Hepatocytes were plated on collagenase-coated six-well plates with 500,000 cells per well, firstly in adherence media (Gibco M199 media, 100 U penicillin/streptomycin, 0.1% BSA, 2% FBS, 100 nmol/l Dexamethasone, 100 nmol/l Insulin) for 4 h, then in EX-CELL^®^ 325 PF CHO Serum-Free medium (Sigma-Aldrich, Castle Hill, Australia) for 16 h. The purity of the hepatocyte isolation was assessed as described previously^8^. Conditioned medium containing all secreted factors was spun at 2000 × *g* for 10 min and the supernatant frozen for subsequent mass spectrometry proteomics assessment and cell culture conditioned media experiments. Hepatocytes were washed in PBS and frozen for mass spectrometric assessment of the intracellular proteome.

### Recombinant ARSA protein production and in vivo experiments

Murine ARSA recombinant protein was generated at Monash University Protein Production Unit (Melbourne, Australia) using a mammalian expression system (Expi293 cells, Thermo Fisher, A14635) and purified under native conditions in 100 mM NaPO_4_ and 150 mM NaCl. The presence of glycosylation was tested by immunoblotting. Recombinant protein was freeze-dried, resuspended at 0.5 mg/mL, and stored at −80 °C, avoiding repeated freeze-thawing. For acute experiments, recombinant protein was injected i.p. at 1.0 mg/kg body weight 60 min before oral glucose challenge (2 g/kg body weight for C57BL/6, 1 g/kg body weight for db/db mice). For assessment of plasma ARSA protein levels, C57BL/6 mice were injected with ARSA (1 mg/kg body weight, i.p.) and tail vein blood was collected at various times over 24 h for subsequent assessment of plasma ARSA by ELISA (Aviva Systems Biology). Plasma ARSA was further assessed by ELISA in lean and HFD-fed C57BL/6 mice and in mice with hepatic overexpression of ARSA or ARSA(C68S) or hepatic ARSA knockdown (see below).

### Adeno-associated virus production and in vivo experiments

Adeno-associated virus (AAV) was generated at Vector Biolabs (Malvern, PA, USA). An AAV serotype 8 (AAV8) driven by a TBG promoter, containing a cDNA sequence specific for murine ARSA (AAV8-TBG-m-ARSA) or ARSA(C68S) (AAV8-TBG-m-ARSA(C68S)), was injected via the tail vein at 1 × 10^12^ gc/mouse. Control mice were injected with the same vector (AAV8-TBG-Null). For knockdown experiments, an AAV8 was used containing a shRNA sequence for ARSA-specific knockdown (AAV8-GFP-U6-m-ARSA shRNA). Control mice were injected with the same vector containing scrambled shRNA (AAV8-GFP-U6-scrmb-shRNA). Metabolic phenotyping was conducted 8 weeks after AAV injection.

### Body composition and energy expenditure

Fat and lean mass were measured using the EchoMRI-900 Body Composition Analyser (EchoMRI Corporation Pte Ltd, Singapore) in accordance with the manufacturer’s instructions. Whole-body energy expenditure, the respiratory exchange ratio, food intake, and locomotor activity were assessed using a 16 chamber Promethion Metabolic Cage System (Sable Systems International). Studies were commenced after 8 h of acclimation to the metabolic chamber, and metabolic parameters assessed at 30 min intervals for 48 h.

### Glucose tolerance and insulin-tolerance tests

Mice were fasted for 4 hours, then gavaged orally with glucose (2 g/kg body weight for C57BL/6 mice and 1 g/kg body weight for db/db mice) or injected i.p. with insulin (1 or 2 units/kg body weight for C57BL/6 and db/db mice, respectively). Blood glucose levels were monitored (Accu-Chek II glucometer; Roche Diagnostics, Castle Hill, New South Wales, Australia), and plasma insulin determined by ELISA (Ultra-Sensitive Mouse Insulin ELISA, Crystal Chem, IL, USA). For assessment of insulin-stimulated glucose disposal, mice fasted for 4 h, then injected i.p. with 1 U/kg body weight (C57BL/6) or 2 U/kg body weight (db/db) insulin and 10 µCi 2-[1-^14^C]-deoxyglucose/mouse (PerkinElmer, Melbourne, Australia). Blood (10 µL per time point) was deproteinized by addition of 20 µL 5% (w/v) ZnSO_4_ and 20 µL saturated BaOH, and centrifugation at 3500 × *g* for 10 min, with radioactivity assessed in the supernatant. Tissue-specific 2-DG uptake was determined as described previously^64^. Briefly, tissues (~30 mg) were homogenized in 1.4 mL 2.75% (w/v) ZnSO_4_, centrifuged at 28,000 × *g* for 10 min, and radioactivity in 400 µL of the supernatant determined (total counts). A further 400 µL of the supernatant was added to 400 µL of saturated BaOH, centrifuged at 28,000 × *g* for 10 min, and again radioactivity in 400 µL of the supernatant determined (free counts). Phosphorylated counts (i.e., 2-DG uptake) were calculated as total counts- free counts. For assessment of LPA impact on insulin sensitivity, ARSA-AAV mice were injected with 200 ng LPA (i.p.) 30 min before an insulin-tolerance test, while ARSA-shRNA mice were dosed orally at 30 mg/kg with the autotaxin inhibitor PF8330 3 h before an insulin-tolerance test^36^. In the latter experiment, plasma autotaxin activity was assessed as recently described^35^. Briefly, 5 µL of plasma was added to 25 µL of 100 mmol/L Tris-HCl (pH 9.0), 500 mmol/L NaCl, 5 mmol/L MgCl_2_, and 0.05% v/v Triton X-100, samples incubated at 37 °C for 30 min, followed by the addition of 25 µL of 6 mmol/L 1-myristoyl-2-hydroxy-sn-glycero-3-phosphocholine (14:0 LPC; Avanti) and incubation at 37 °C for 12 h to allow for ATX-mediated choline release. The next day, 25 µL of each reaction mixture was added to 90 µL of buffer {9.65 ml of 100 mmol/L Tris-HCl (pH 8.5) and 5 mmol/L CaCl_2_, containing 110 µL of 30 mmol/L N-ethyl-N-(2-hydroxy-3-sulfopropyl)-3-methylaniline (TOOS; Sigma-Aldrich), 110 µL of 50 mmol/L 4-aminotipyrine, 6.6 µL of 1000 U/mL: horseradish peroxidase, and 110 µL of 300 U/ml choline oxidase} in a 96 well plate and choline oxidation was measured at 37 °C and 550 nm for 10 min.

### Plasma analysis

Plasma non-esterified fatty acids (NEFAs) and total plasma cholesterol were measured by colorimetric assays (Wako Diagnostics, Osaka, Japan). Plasma triglyceride content was determined by colorimetric assay (Triglycerides GPOPAP; Roche Diagnostics, Indianapolis, IN). Plasma β-hydroxybutyrate levels were measured by colorimetric assay (Sapphire Biosciences, Redfern, NSW, Australia). Tumor necrosis factor-α (TNFα) and interleukin 6 (IL6) were measured by ELISA (product codes EK-0005 & EK-0029, ELISAkit.com; Melbourne, Victoria, Australia). Plasma LPA was assessed by ELISA (Aviva Systems Biology, CA, USA). Autotaxin activity in plasma, lipid raft fractions, and liver conditioned media was assessed, as recently described^35^ and as detailed above.

### Precision-cut liver slices

As for the human liver secretion studies, a piece of liver from the same liver lobe of Control-AAV and ARSA-AAV mice was embedded in 3% agarose (SeaPlaque™ Agarose, Lonza Bioscience) and 300-μm-thick liver slices were generated using a Krumdieck Tissue Slicer (TSE Systems China Ltd). Liver slices were maintained in oxygenated M199 media (Thermo Fisher Scientific, Scoresby, Australia) for 60 min before collection of secreted factors in oxygenated protein-free EX-CELL^®^ 325 PF CHO Serum-Free medium (Sigma-Aldrich, Castle Hill, Australia) for 16 h. This conditioned medium (CM) was utilized for lipidomics assessment, as well as applied to C2C12 myotubes for subsequent analysis of myotube glucose uptake.

### Cell culture and conditioned media experiments

C2C12 myoblasts (ATCC, CRL-1772) were grown in 1:1 Dulbecco’s modified Eagle’s medium (DMEM) and Ham’s F-12 nutrient mix (Life Technologies, Mulgrave, Australia) containing 10% (v/v) FBS (Life Technologies) and 1% penicillin/streptomycin. Differentiation was induced by serum starvation with 2% horse serum for 5–7 days. 3T3-L1 pre-adipocytes (ATCC, CL-173) were cultured in high glucose DMEM (Life Technologies) containing 10% (v/v) FBS (Life Technologies) and 1% penicillin and streptomycin. Once 100% confluence was reached, cells were differentiated using 3-isobutyl-1-methylxanthine (500 μM; Sigma-Aldrich), dexamethasone (5 μM; Sigma-Aldrich), biotin (0.1 μg/mL) and insulin (0.05 U/mL) for 3 days, followed by culturing with insulin (0.05 U/mL) in DMEM containing 10% FBS and 1% antibiotics for a further 3 days. Primary murine hepatocytes were isolated from lean C57BL/6 mice as described above, and following 4 h in adherence media, were cultured in M199 media supplemented with 100 U penicillin/streptomycin and 100 nmol/L dexamethasone for 24 h.

Conditioned media (CM) collected from Control and NASH hepatocytes (from MCD and CHOL-fed mice) was applied to myotubes, hepatocytes, and adipocytes at a ratio of 1:1 (50% CM, 50% cell-specific culture media, excluding insulin or serum) for 24 h before metabolic assessment. Following CM treatment, cells were stimulated with 50 nM insulin for 10 min, washed in ice-cold PBS, and collected for immunoblot analysis. Fatty acid metabolism was assessed using 1-[^14^C]-oleic acid, as recently described^65^. For insulin-stimulated glucose uptake, cells were cultured in glucose-free DMEM containing 1 mM 2-deoxyglucose and 0.1% bovine serum albumin (BSA) for 30 min. The medium was refreshed with 50 nM insulin for 10 min, followed by glucose-free DMEM containing 1 mM 2-deoxyglucose, 0.1% BSA, and 1 µCi/ml 1-[^14^C]-2-deoxyglucose (PerkinElmer) for a further 10 min. For all experiments, radioactivity was counted on a Packard Tri-Carb Liquid Scintillation Counter (PerkinElmer). For assessment of lipolysis, adipocytes were incubated in phenol red-free low-glucose DMEM containing 2% BSA, in the absence or presence of 1 mM isoproterenol for 2 h. The medium was assessed for glycerol content (Free Glycerol Reagent, Sigma-Aldrich).

### Gene expression analysis

RNA was extracted using TRI-Reagent (Sigma-Aldrich) and treated with a DNA removal kit (Ambion DNA free kit, Thermo Fisher, VIC, Australia), following the manufacturer’s instructions. Extracted RNA was reverse transcribed using iSCRIPT Reverse Transcriptase (Invitrogen, USA). Real-time PCR was performed using a SYBR Green PCR Mastermix (Quantinova^®^ SYBR Green PCR kit, QIAGEN, Germany) and gene expression was determined using a CFX Connect™ Real-Time PCR Detection System (Biorad). All samples were normalized using the housekeeping gene *Hprt*. Primer sequences are listed in Supplementary Table S8.

### Immunoblot analysis

Tissues, cell lysates, plasma, and conditioned media were prepared in radioimmunoprecipitation assay (RIPA) buffer, lanes were loaded with equal total protein content, resolved by SDS-PAGE, and immunoblot analysis was conducted as described previously^66^. Primary antibodies are listed in Supplementary Table S9. For the analysis of ARSA and ApoB secretion from liver slices, media was dried down using a SpeedVac Concentrator, resuspended in RIPA buffer, and proteins resolved by SDS-PAGE. The volume of media was adjusted for tissue mass. Equal protein loading is shown as total protein content across the entire membrane, as assessed using Biorad Mini-PROTEAN TGX Stain-Free Gels system (trihalo compounds in gel react with tryptophan residues in proteins in a UV-induced reaction to produce fluorescence, with fluorophores remaining bound throughout blotting).

### Lipid raft isolations

Lipid rafts were isolated by sucrose gradient ultracentrifugation from the liver and quadriceps muscle. Tissue was homogenized in 500 mM Na_2_CO_3_ using a Precellys Evolution Tissue Homogenizer (Bertin Instruments, USA). Samples were further homogenized using an ultrasonic cell disruptor and the buoyant lipid raft fraction isolated by floatation on a discontinuous sucrose density gradient. In all, 1 mL of homogenized sample was mixed with 1 mL of 90% sucrose in MES buffered saline (25 mM 2-(N-Morpholino) ethanesulfonic acid 4-Morpholineethane sulfonic acid (MES), 150 mM NaCl, pH 6.0) in a 5 mL Beckman ultraclear ultracentrifuge tube. Layered above was 2 mL of 35% sucrose in MBS/Na_2_CO_3_ (e.g., 5.83 mL 90% sucrose/MBS and 9.17 mL MBS/Na_2_CO_3_) and 1 mL of 5% sucrose in MBS/Na_2_CO_3._ The samples were centrifuged for 16 h at 310,000 × *g* and 4 °C using a 70.1Ti fixed angle rotor in an Optima L-90K Ultracentrifuge (Beckman), equivalent to a maximum force (at the bottom of the tube) of 260,000 × *g* and an average force (middle of the tube) of 200,000 × g. Following the centrifugation step, the buoyant lipid raft fraction is visible at the 35% sucrose – 5% sucrose interface. The samples from the gradient were collected in 0.5-mL aliquots from the top of the tube (and are thereby numbered as fractions 1–10). Each fraction was analyzed by immunoblotting to identify the lipid raft-containing fraction (Thy1 positive) and to assess raft purity (probing for Na,K-ATPase [cell surface, raft and non-raft], ADRB3 [cell surface, non-raft], IRE1 [endoplasmic reticulum], ACC1/2 [cytosol/mitochondria] and beta-actin [cytosol]). Thy1-enriched fractions were used for subsequent proteomics and lipidomics assessment. For proteomics, fractions were directly used for processing. For lipidomics, fractions were diluted to 5 mL in ddH_2_O and ultracentrifuged at 100,000 × *g* for 60 min, to pellet lipid rafts and to remove sucrose.

### Sample processing for lipidomics analysis

The full lipidome was assessed in the whole liver, isolated liver and quadriceps muscle lipid rafts, in liver conditioned media (secreted lipids), and in plasma. Lipids from the whole liver and plasma were extracted using a monophasic extraction protocol, as recently described^67^. Briefly, 10 mg of tissue or 10 µL of plasma were homogenized using a Precellys tissue homogenizer in 100 µL 1:1 butanol-methanol, containing the following lipid standards (all from Avanti Polar Lipids Inc.): 10 µL of SPLASH^®^ II LIPIDOMIX^®^ Mass Spec Standard (part no. 330709W), 10 μL of Ceramide LIPIDOMIX^®^ Mass Spec Standard (part no. 330712X), 1 µg C18:0 GM3 Ganglioside-d5 (part no. 860073W), and 0.5 µg C12 Mono-Sulfo Galactosyl(ß) Ceramide (sulfatide) (d18:1/12:0) (part no. 860573P), samples were vortexed thoroughly for 1 h at room temperature, centrifuged (14,000 × *g*, 10 min, 20 °C) and transferred into sample vials with glass inserts for analysis.

Lipids from conditioned media and isolated lipid rafts were extracted using a biphasic extraction method. Briefly, each sample was homogenized in 1 mL 2:1 (v:v) chloroform-methanol containing the same amount of internal standards, as above, followed by phase separation after adding 0.5 mL 0.9% NaCl and centrifugation at 900 × *g* for 10 min. The complete bottom lipid layer was transferred into glass vials and fully dried under a stream of nitrogen gas. Lipids were resuspended in 100 µL methanol, centrifuged again at 10,000 × *g* for 10 min, and the supernatant was transferred into sample vials with glass inserts for analysis.

### Mass spectrometric lipid analysis

Samples were analyzed by ultrahigh performance liquid chromatography (UHPLC) coupled to tandem mass spectrometry (MS/MS) employing a Vanquish UHPLC linked to an Orbitrap Fusion Lumos mass spectrometer (Thermo Fisher Scientific), with separate runs in positive and negative ion polarities. Solvent A was 6/4 (v/v) acetonitrile/water with 10 mM ammonium acetate and 5 µM medronic acid and solvent B was 9/1 (v/v) isopropanol/acetonitrile with 10 mM ammonium acetate. Each sample was injected into an RRHD Eclipse Plus C18 column (2.1 × 100 mm, 1.8 µm, Agilent Technologies) at 60 °C at a flow rate of 350 μl/min for 3 min using 30% solvent B. During separation, the percentage of solvent B was increased from 30% to 70% in 5 min, from 70 to 93% in 9 min and, from 93 to 99% in 7 min, and from 91 to 97% in 31 min. Subsequently, the percentage of solvent B was increased to 99.5% in 0.1 min and then maintained at 99.5% for 3 min. Finally, the percentage of solvent B was decreased to 30% in 0.1 min and maintained for 3.9 min.

All MS experiments were performed using a Heated Electrospray Ionization (HESI) source. The spray voltages were 3.5 kV in positive ionization mode and 3.0 kV in negative ionization mode. In both polarities, the flow rates of the sheath, auxiliary and sweep gases were 20 and 6 and 1 arbitrary unit(s), respectively. The ion transfer tube and vaporizer temperatures were maintained at 350 and 400 °C, respectively, and the S-Lens RF level was set at 50%. In the positive ionization mode from 3 to 24 min, top speed data-dependent scan with a cycle time of 1 s was used. Within each cycle, full-scan MS spectra were acquired firstly in the Orbitrap at a mass resolving power of 120,000 (at *m/z* 200) across an m/z range of 300–2000 using quadrupole isolation, an automatic gain control (AGC) target of 4e5 and a maximum injection time of 50 milliseconds, followed by higher-energy collisional dissociation (HCD)-MS/MS at a mass resolving power of 15,000 (at *m/z* 200), a normalized collision energy (NCE) of 27% at positive mode and 30% at negative mode, an *m/z* isolation window of 1, a maximum injection time of 22 milliseconds and an AGC target of 5e4. For the improved structural characterization of glycerophosphocholine (PC) lipid cations, a data-dependent product ion (*m/z* 184.0733)-triggered collision-induced dissociation (CID)-MS/MS scan was performed in the cycle using a *q* value of 0.25 and a NCE of 30%, with other settings being the same as that for HCD-MS/MS. For the improved structural characterization of triacylglycerol (TG) lipid cations, the fatty acid + NH_3_ neutral loss product ions observed by HCD-MS/MS were used to trigger the acquisition of the top-3 data-dependent CID-MS^3^ scans in the cycle using a *q* value of 0.25 and a NCE of 30%, with other settings being the same as that for HCD-MS/MS.

To improve the identification of gangliosides and sulfatides, additional targeted mass spectrometry experiments were performed. Briefly, a pooled collection from each sample set was run in negative mode using the parameters described above in advance. A parent ion search for the pooled sample was done through LipidSearch 4.2.23 with the following search parameters: target database, general; precursor tolerance, 5 ppm; adduct ions, −H and −2H for negative ionization mode; lipid class: GM1, GM2, GM3, GD1a, GD1b, GD2, GD3, GT1a, GT1b, GT2, GT3, GQ1c, GQ1b, ST. The resultant *m/z* values of ganglioside and sulfatide candidates were used for a targeted LC-MS/MS experiment in negative mode which was run together with each sample set. All parameters were identical to above with the only exception being that the top speed data-dependent scan was replaced with a cycle consisting of one full-scan MS spectra and up to 35 targeted MS/MS scans of ganglioside or sulfatide candidates. A mass resolving power of 7500 (at *m/z* 200), a first mass at *m/z* 50, normalized collision energy (NCE) of 50% for sulfatide and 30% for ganglioside were used for targeted MS/MS scans.

### Identification and quantification of lipids and statistical analysis

MS data were processed using LipidSearch software v.4.2.23 (Thermo Fisher Scientific). Key processing parameters were: target database, general; precursor tolerance, 5 ppm; product tolerance, 10 ppm; product ion threshold, 1%; m-score threshold, 2; quantification m/z tolerance, ±5 ppm; quantification retention time range, ±1 min; use of main isomer filter and ID quality filters A, B and C; adduct ions, +H and +NH_4_, + Na and +H–H_2_O for positive ionization mode, and −H, + CH_3_COO, −2H and −CH3 for negative ionization mode. CL, LPA, PA, LPC, PC, LPE, PE, LPG, PG, LPI, PI, LPS, PS, Cer, CerP, CerPE, Hex1SPH, Hex1Cer, Hex2Cer, Hex3Cer, SM, SPH, SPHP, ST, ChE, MG, DG, TG, AcCa, Co and FA (see Supplementary Table S10 for the full name and ion form used for quantification for each lipid class selected in LipidSearch 4.2.23) were selected for the search. The same lipid annotations (within 0.1 min of retention time and 5 ppm of mass error) were merged into the aligned results. Unassigned peak areas were calculated for relative quantification and alignment. The shorthand notation used for lipid classification and structural representation follows the nomenclature proposed previously^68^. For hexosyl ceramide (Hex1Cer), monoacylglycerol (MG) and sphingomyelin (SM) lipids, a grade A-, B-, or C-identification was required in at least one sample, whereas for all other lipid classes, only a grade A- or B-identification was required in at least one sample. For all lipids, a ratio of MS/MS spectrum peaks assigned to the lipid among all fragmentation product ions (i.e., MainOccupy value) >40 was required in at least one sample. Only sphingolipids consisting of a sphingoid base with two hydroxyl groups (i.e., having “d” in lipid name reported by LipidSearch) were accepted since this is the dominant form of sphingolipids in animals. All lipid LC-MS features were manually inspected and re-integrated when needed. Sulfatides were identified if the characteristic product ion at *m/z* 96.9595 (i.e., HSO_4_^−^) was detected within 10 ppm mass error and the intact lipid was detected within 5 ppm mass error using Xcalibur Qual Browser (Thermo Fisher Scientific). Peak areas of extracted ion chromatogram of identified sulfatide lipid ions were extracted using Skyline (19.1.0.193).

Relative quantification of lipid species was achieved by comparison of the LC peak areas of identified lipids against those of the corresponding internal lipid standards in the same lipid class, and the resultant ratio of peak area was then normalized to the weight of tissue, volume of plasma or protein content (for lipid raft fractions). For the lipid classes without correspondent isotope-labeled internal lipid standards, the LC peak areas of individual molecular species within these classes were normalized as follows: the MG species against the DG (18:1D7_15:0) internal standard; the CL, LPG, PG, LPA and PA against the PI (18:1D7_15:0) internal standard; the LPS against the PS (18:1D7_15:0) internal standard; the Hex1Cer against the SM (d36:2D9) internal standard. Given that only a single lipid standard per class was used, some of the identified lipids were normalized against a standard from a different class or sub-class, and no attempts were made to quantitatively correct for different ESI responses of individual lipids due to concentration, acyl chain length, degree of unsaturation, or matrix effects caused by differences in chromatographic retention times compared with the relevant standards. The results reported here are for relative quantification and should not be considered to reflect the absolute concentrations of each lipid or lipid sub-class. All lipidomics data are provided with the paper as Supplementary Data 1.

### Sample processing for proteomics analysis

For assessment of hepatocyte-secreted factors, conditioned media was concentrated using Amicon Ultra-4 Centrifugal Filters at 4000 × *g* for 45 min, the concentrated sample was washed in 2 mL 50 mM Tris, pH 8.0 + 150 mM NaCl at 4000 × *g* for 45 min, transferred to Eppendorf tubes and the protein content was determined. Disulfide bonds were reduced through the addition of 10 mM TCEP (Tris(2-carboxyethyl)phosphine) at 65 °C for 20 min. The sample was mixed with 6.6× volume of 5 M urea, added to Microcon-30 kDa Centrifugal Filter (Sigma-Aldrich), and spun at 14,000 × *g* and 10 °C for 15 min. This was followed by the addition of 200 µl 5 M Urea, centrifugation at 14,000 × *g* and 10 °C for 15 min, discarding the flow-through. Chloroacetamide was added to a final concentration of 10 mM for the spin filters to alkylate proteins, samples were incubated for 20 min in the dark, and spun again. Proteins were washed 3× with 100 µL 5 M urea and 3× with 100 µL 50 mM ammonia bicarbonate (pH 8.5), each wash step was followed by centrifugation at 14,000 × *g* and 10 °C for 15 min, discarding the flow-through. After the last wash step, a digestion solution was added to the spin filter (1 µg trypsin/100 µg protein in 75 µL of 50 mM ammonia bicarbonate) and samples incubated at 37 °C overnight. The following day, peptides were eluted by addition of 2 × 40 µL of 50 mM ammonia bicarbonate, and 1 × 40 µL 0.5 M NaCl, each step was followed by centrifugation at 14,000 × *g* and 10 °C for 15 min. Elute peptide was acidified with formic acid to final pH 3.0, and peptides were purified with Millipore^®^ C18 Ziptips (Sigma-Aldrich). This purification step involved activation of Ziptips with methanol, washing with 0.1% formic acid, binding of peptides to Ziptips, a second wash step in 0.1% formic acid, and final elution into 200 µL 50% acetonitrile/0.1% formic acid. Samples were dried in a SpeedVac concentrator (Eppendorf Concentrator Plus), resuspended in 20 µL 0.1% formic acid, sonicated for 10 min at room temperature, centrifuged at 16,000 × *g* for 10 min, and transferred into HPLC vials for analysis. For assessment of the liver and lipid raft proteome, samples were homogenized in 50 mM Tris, pH 8.0 + 150 mM NaCl, followed by assessment of protein content and reduction of disulfide bonds as above. All subsequent steps were as above for conditioned media processing.

### Mass spectrometry proteomics analysis

#### Assessment of hepatocyte-secreted proteins

All samples/peptides were analyzed by LC-MS/MS using a Q Exactive Plus mass spectrometer (Thermo Fisher Scientific) coupled online to a RSLC nano HPLC (Ultimate 3000, UHPLC Thermo Fisher Scientific). Samples were loaded onto a 100 µm, 2-cm nanoviper Pepmap100 trap column, eluted and separated on a RSLC nano column 75 µm × 50 cm, Pepmap100 C18 analytical column (Thermo Fisher Scientific). The detailed gradients used for protein identification are listed in Supplementary Table S11. The eluent was nebulized and ionized using a nanoelectrospray source (Thermo Fisher Scientific) with a distal coated fused silica emitter (New Objective). The capillary voltage was set at 1.7 kV. The Q Exactive mass spectrometer was operated in the data-dependent acquisition mode to automatically switch between full MS scans and subsequent MS/MS acquisitions. Survey full-scan MS spectra (*m/z* 375–1575) were acquired in the Orbitrap with 70,000 resolution (at *m/z* 200) after accumulation of ions to a 3 × 10^6^ target value with a maximum injection time of 54 ms. Dynamic exclusion was set to 15 s. The 12 most intense multiply charged ions (z ≥ 2) were sequentially isolated and fragmented in the collision cell by higher-energy collisional dissociation (HCD) with a fixed injection time of 54 ms, 17,500 resolution, and automatic gain control (AGC) target of 2 × 10^5^.

#### Assessment of tissue and lipid raft proteome

Peptide samples were analyzed by LC-MS/MS using an Orbitrap mass spectrometer (Thermo Fisher Scientific) coupled online to a RSLC nano HPLC (Ultimate 3000 UHPLC, Thermo Fisher Scientific). Samples were loaded onto a Pepmap100 C18 trap column (75 µm × 2 cm, Thermo Fisher Scientific) at 50 °C using an isocratic flow of 3% acetonitrile (ACN)/0.05% trifluoroacetic acid (TFA) at 5 μL/min for 6 min, eluted and separated on a Pepmap100 C18 analytical column (75 µm × 50 cm, Thermo Fisher Scientific) at 50 °C using a flow rate of 300 nL/min. The eluents used for the LC were water with 0.1% formic acid (FA) and 5% dimethyl sulfoxide (DMSO) for solvent A and ACN with 0.1% FA and 5% DMSO for solvent B.

For the NASH hepatocyte proteome and quad lipid raft proteome, samples were analyzed on Q Exactive Plus Orbitrap mass spectrometer (Thermo Fisher Scientific). The spray voltage, the temperature of ion transfer tube and S-lens were set at 1.9 kV, 250 °C, and 70%, respectively. The full MS scans were acquired at *m/z* 375–1400, a resolving power of 70,000, an AGC target value of 3.0 × 10^6^ and a maximum injection time of 50 milliseconds. The top 15 most abundant ions in each full-scan MS spectrum were subjected to HCD at a resolving power of 17,500, AGC target value of 5 × 10^4^, maximum injection time of 50 milliseconds, isolation window of *m/z* 1.2, and NCE of 30%. Dynamic exclusion of 30 s was enabled. The detailed gradients used for each protein identification are listed in Supplementary Table S12. Details on mass spectrometry platforms utilized in each proteomics and lipidomics experiment are detailed in Supplementary Table S13.

All generated files were analyzed with MaxQuant (version 1.5.3.30)^69^ and its implemented Andromeda search engine to obtain protein identifications as well as their label-free quantitation (LFQ) intensities. Database searching was performed with the following parameters: cysteine carbamidomethylation as a fixed modification; up to 2 missed cleavages permitted; mass tolerance of 20 ppm; 1% protein false discovery rate (FDR) for protein and peptide identification; and minimum two peptides for pair-wise comparison in each protein for label-free quantitation. The MaxQuant result output was further processed with Perseus (Version 1.5.0.40)^70^, a module from the MaxQuant suite. After removing reversed and known contaminant proteins, the LFQ values were log_II_ transformed and the reproducibility across the biological replicates was evaluated by a Pearson’s correlation analysis. The replicates were grouped accordingly, and all proteins were removed that had less than two “valid value” in each group. The missing values were replaced by imputation and a two-sample *t* test (FDR < 5%) was performed to obtain a list of significantly regulated proteins. All proteomics data are provided with the paper as Supplementary Data 2.

### Statistical analysis

All measurements were taken from distinct samples, with no sample being measured repeatedly. Data are presented as means ±  standard error of the mean (SEM). Data were assessed for normal distribution using the D’Agostino & Pearson test with parametric tests used in cases of normal distribution, and nonparametric tests used in cases of non-normal distribution. Parametric tests included a two-tailed unpaired Students *t* test, one or two-way analysis of variance (ANOVA) followed by Bonferroni multiple comparisons tests where appropriate. For nonparametric testing, a Spearman’s rank correlation coefficient was used to test the relationship between two variables and a Mann–Whitney test used to assess differences between a single variable. Statistical significance was determined a priori as *P* < 0.05.

### Reporting summary

Further information on research design is available in the Nature Research Reporting Summary linked to this article.

## Supplementary information

Supplementary Information

Description of Additional Supplementary Files

Supplementary Data 1

Supplementary Data 2

Reporting Summary

## Data Availability

All uncropped immunoblotting images can be found within the supplementary files. Similarly, proteomics and lipidomics data are supplied as supplementary excel files. In addition, the proteomics data generated in this study have been deposited in the ProteomeXchange database under the following accession codes: Set 1: Project Name: Analysis of mouse liver secretome and proteome during nonalcoholic steatohepatitis [Project accession: PXD024673]; Set 2: Proteomic analysis of ARSA overexpression [Project accession: PXD026280].  Source data are provided with this paper.

## Source data

Source Data
