# Supplementary material for: Deep proteomic profiling unveils arylsulfatase A as a non-alcoholic steatohepatitis inducible hepatokine and regulator of glycemic control
Source: Nat Commun. 2022 Mar 10;13:1259. doi: 10.1038/s41467-022-28889-2 (PMC8913628; doi:10.1038/s41467-022-28889-2)

Deep proteomic profiling unveils arylsulfatase A as a non-alcoholic steatohepatitis inducible hepatokine and regulator of glycemic control

<u>SUPPLEMENTARY DATA</u>

1. Supplementary Figures

Figure S1

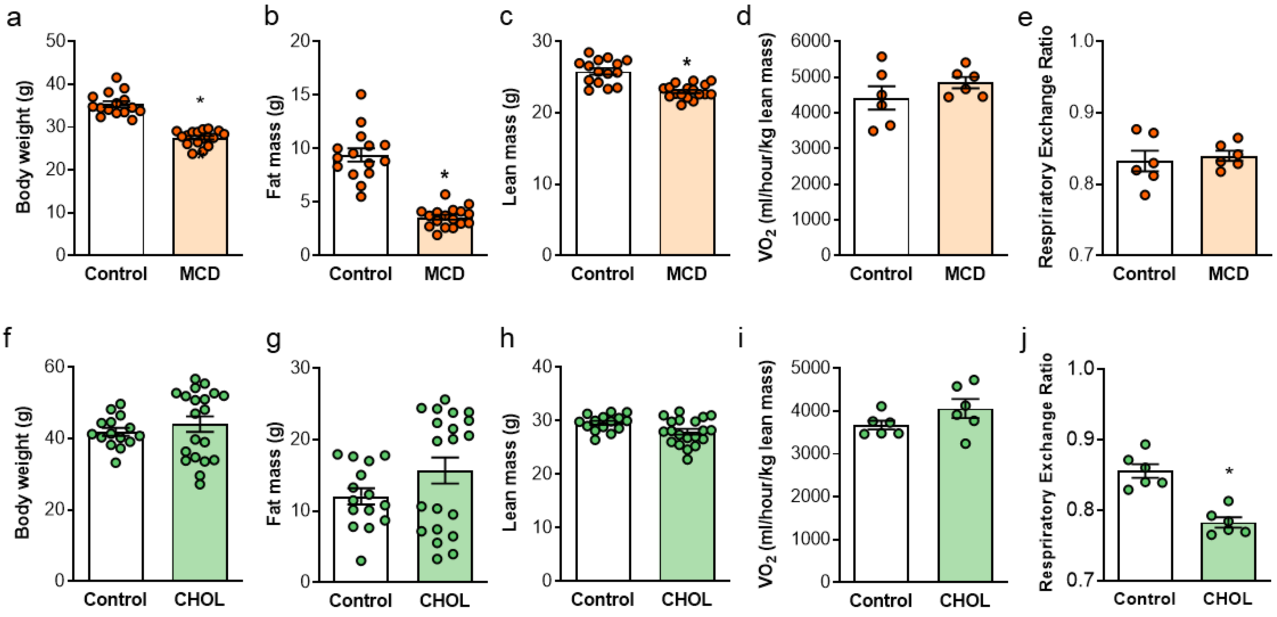

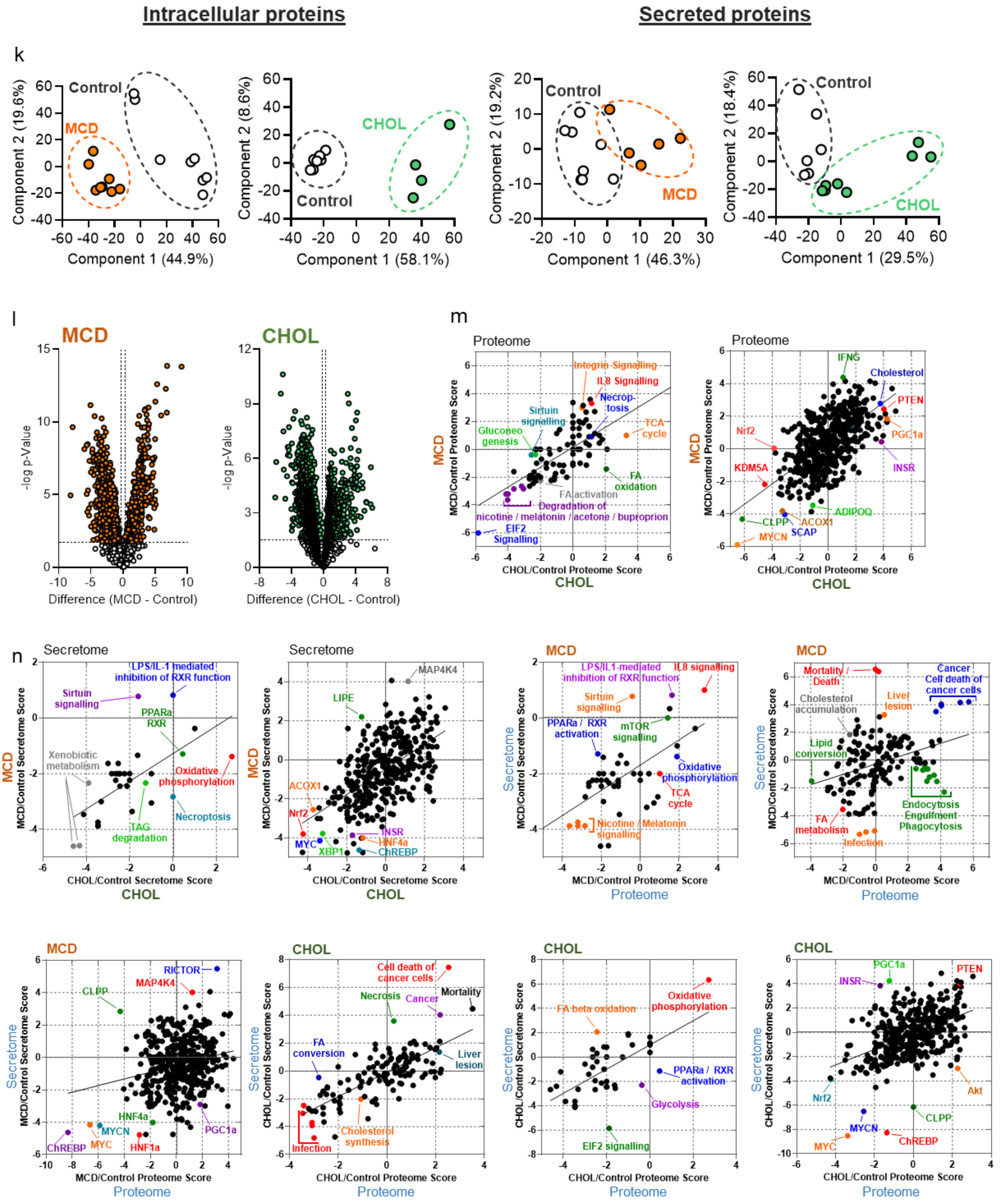
Intracellular proteins
Secreted proteins
k
Control
MCD
CHOL
Component 1 (44.9%)
Component 2 (19.6%)
Component 1 (58.1%)
Component 2 (8.6%)
Component 1 (46.3%)
Component 2 (19.2%)
Component 1 (29.5%)
Component 2 (18.4%)
l
MCD
CHOL
-log p-Value
Difference (MCD - Control)
Difference (CHOL - Control)
m
Proteome
MCD/Control Proteome Score
CHOL/Control Proteome Score
n
Secretome
MCD/Control Secretome Score
CHOL/Control Secretome Score
Integrin Signalling
IL8 Signalling
Necroptosis
Sirtuin signalling
Gluconeogenesis
TCA cycle
FA oxidation
FA activation
Degradation of nicotine / melatonin / acetone / buproprion
EIF2 Signalling
IFNG
Cholesterol
PTEN
Nrf2
PGC1a
KDM5A
INSR
ADIPOQ
CLPP
ACOX1
SCAP
MYCN
LPS/IL-1 mediated inhibition of RXR function
PPARa RXR
Xenobiotic metabolism
Oxidative phosphorylation
Necroptosis
TAG degradation
MAP4K4
LIPE
MYC
XBP1
HNF4a
ChREBP
PPARa / RXR activation
mTOR signalling
Nicotine / Melatonin signalling
Mortality / Death
Cancer Cell death of cancer cells
Cholesterol accumulation
Liver lesion
Lipid conversion
Endocytosis Engulfment Phagocytosis
FA metabolism
Infection
RICTOR
HNF1a
Cell death of cancer cells
Necrosis
Cancer
Mortality
FA conversion
Liver lesion
Cholesterol synthesis
FA beta oxidation
Glycolysis
EIF2 signalling
Akt
Proteome

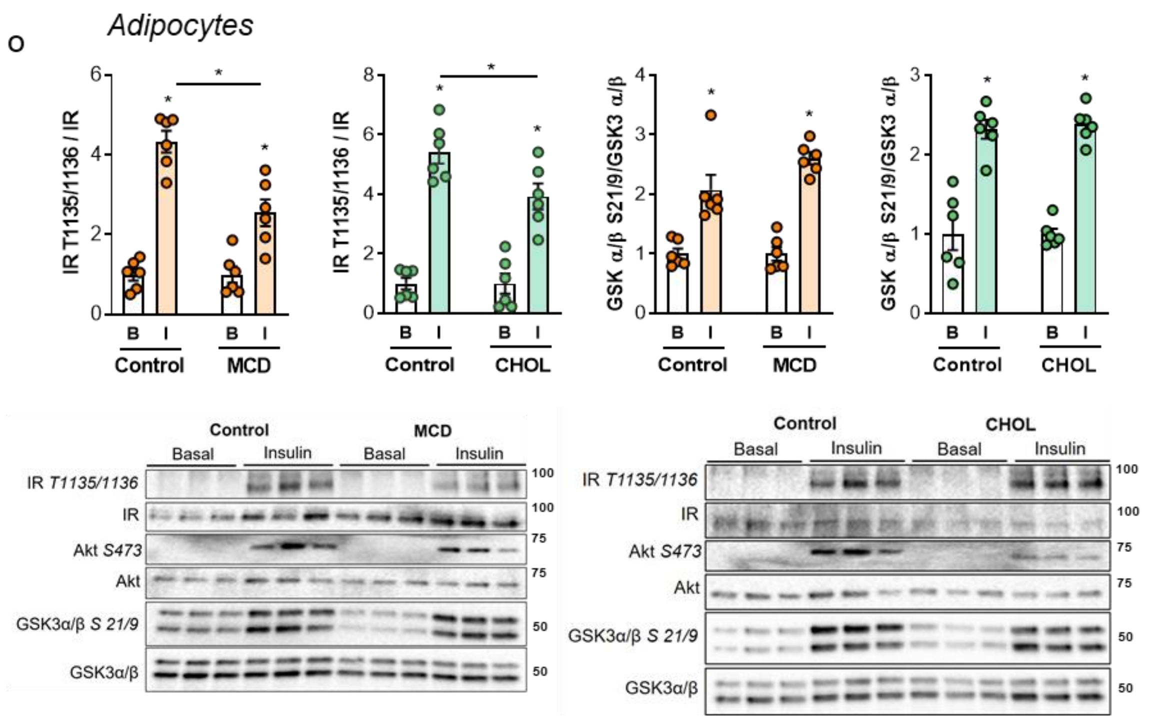
o
Adipocytes
IR T1135/1136 / IR
GSK α/β S21/9/GSK3 α/β
B
I
Control
MCD
CHOL
Basal
Insulin
IR T1135/1136
IR
Akt S473
Akt
GSK3α/β S 21/9
GSK3α/β
100
75
50

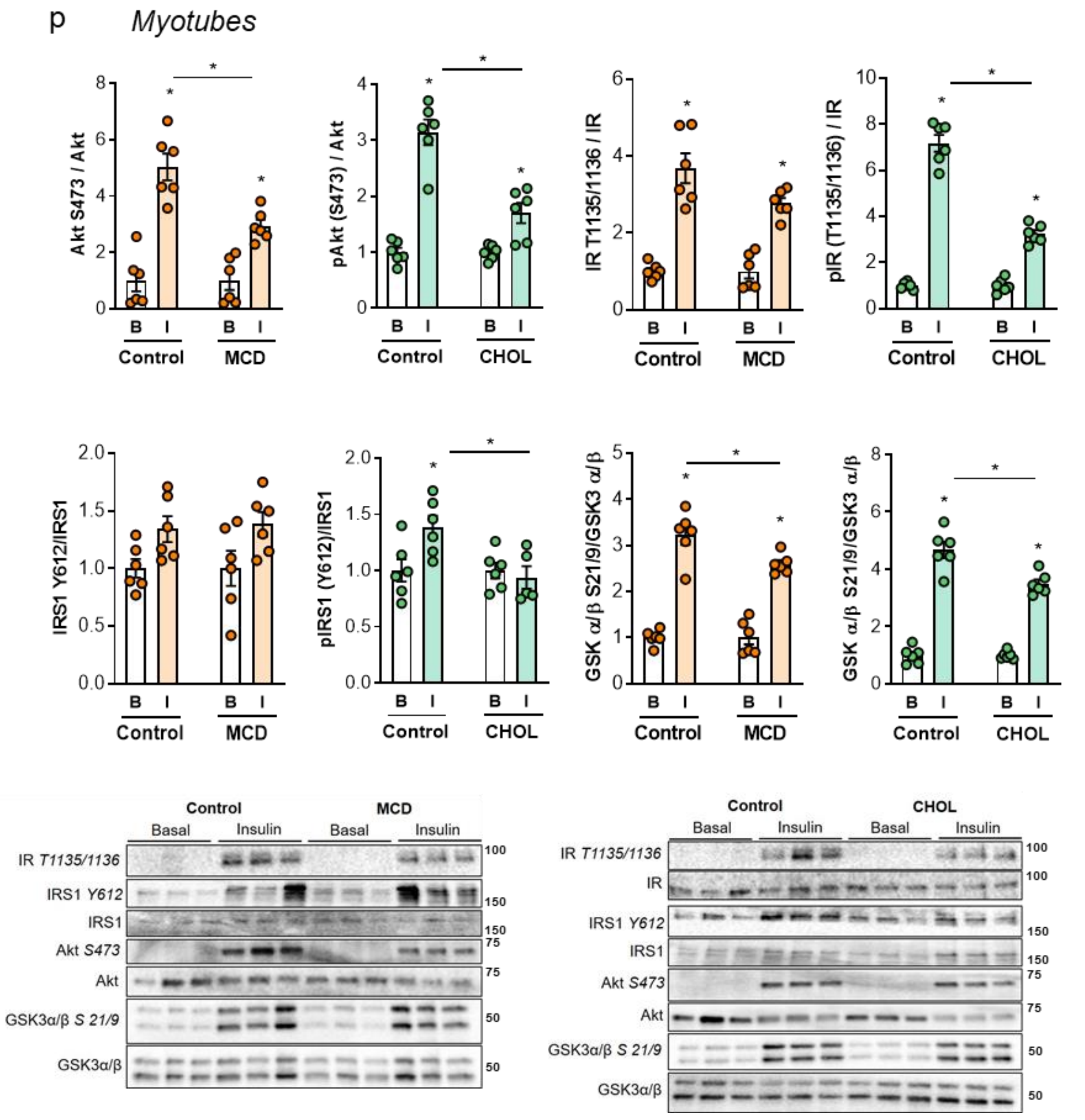
p
Myotubes
Akt S473 / Akt
pAkt (S473) / Akt
IR T1135/1136 / IR
pIR (T1135/1136) / IR
IRS1 Y612/IRS1
pIRS1 (Y612)/IRS1
GSK α/β S21/9/GSK3 α/β
B
I
Control
MCD
CHOL
Basal
Insulin
IR T1135/1136
IRS1 Y612
IRS1
Akt S473
Akt
GSK3α/β S 21/9
GSK3α/β
IR
100
150
75
50

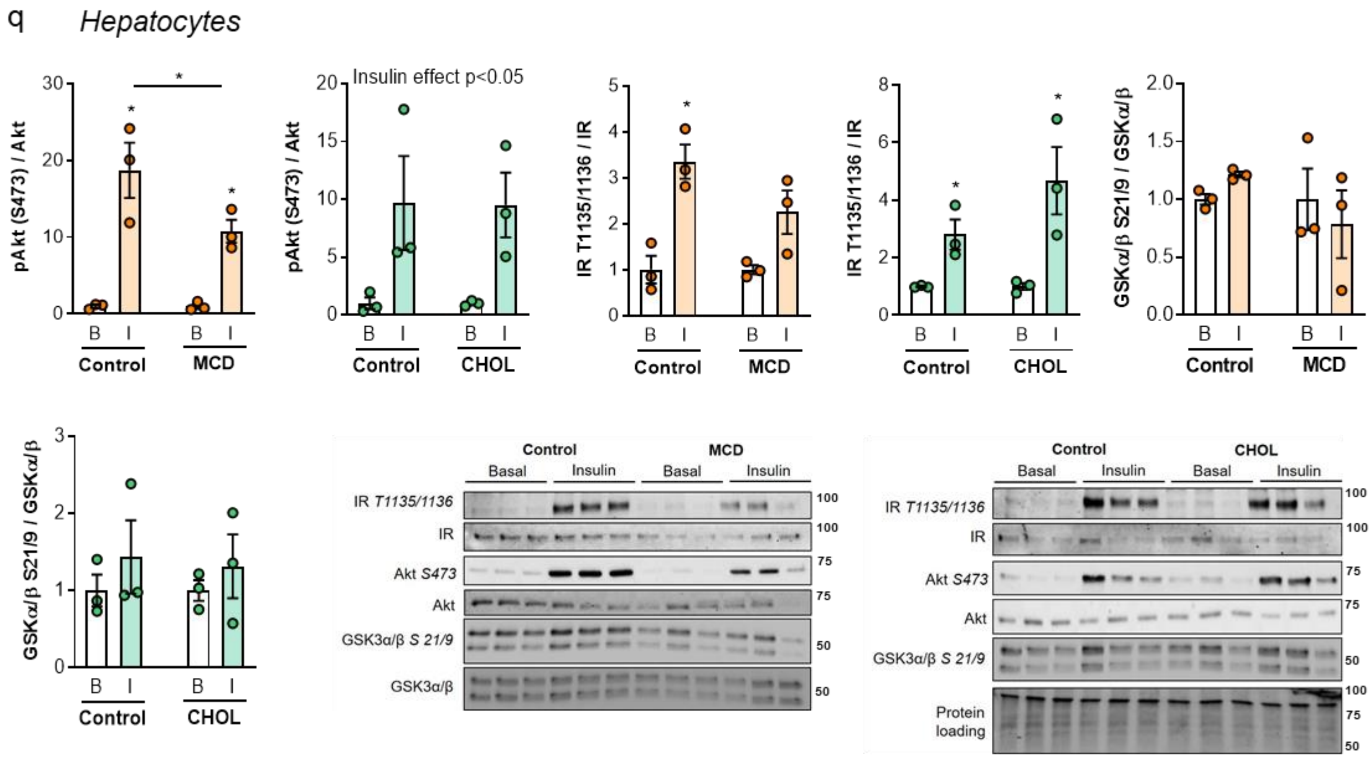

Figure S1. Identification and characterization of NASH-secreted proteins. C57BL/6 mice were fed either a methionine- and choline-deficient diet (MCD, orange) for 10 weeks or a diet enriched in lipid, fructose and cholesterol (CHOL, green) for 40 weeks. Control mice received a standard diet (as detailed in methods) and were age matched. **(A)** Body weight (n=15 Control, n=17 MCD; *P<0.0001), **(B)** fat mass (n=15 Control, n=17 MCD; *P<0.0001), **(C)** lean mass (n=15 Control, n=17 MCD; *P<0.0001), **(D)** energy expenditure (assessed as systemic oxygen consumption, n=6/group) and **(E)** respiratory exchange ratio as marker of systemic fat and carbohydrate oxidation (n=6/group) in MCD and respective Control mice. **(F)** Body weight (n=15 Control, n=20 CHOL), **(G)** fat mass (n=15 Control, n=20 CHOL), **(H)** lean mass (n=15 Control, n=20 CHOL), **(I)** energy expenditure (n=6/group) and **(J)** respiratory exchange ratio (n=6/group, *P=0.0001) in CHOL and respective Control mice. **(K-N)** Hepatocytes from MCD (orange) and CHOL mice (green) (and respective Control mice) were isolated, followed by assessment of the intracellular proteome and secretome. **(K)** Principal component analysis (PCA) for intracellular proteome and hepatocyte secretome, as well as **(L)** volcano plots showing significant NASH-regulated proteins (proteins highlighted in orange (MCD) or green (CHOL) are significantly increased or decreased in NASH) within the intracellular proteome. **(M-N)** Qiagen Ingenuity IPA Pathway analysis showing communal NASH-induced changes in canonical pathways, diseases

and function, as well as upstream regulators. For each correlation, selected pathways, diseases and upstream regulators have been highlighted.

(O-Q) Conditioned media from Control and NASH hepatocytes obtained from mice fed the MCD (orange) and CHOL (green) diets was applied to 3T3-L1 adipocytes, C2C12 myotubes and primary murine hepatocytes, followed by assessment of insulin sensitivity. Representative immunoblotting and respective quantification of phosphorylation of insulin receptor (IR, T1135/1136), insulin receptor substrate 1 (IRS1, Y612), Akt (S473) and glycogen synthase kinase 3 α/β (GSK3 α/β, S21/9) in

(O) adipocytes (IR MCD: *P<0.0001 Control B vs. I, *P=0.0018 MCD B vs. I, *P=0.0004 Control I vs. MCD I; IR CHOL: *P<0.0001 Control B vs. I, *P<0.0001 CHOL B vs. I, *P=0.049 Control I vs. CHOL I; GSK MCD: *P=0.0006 Control B vs. I, *P=0.0006 MCD B vs. I; GSK CHOL: *P<0.0001 Control B vs. I, *P<0.0001 CHOL B vs. I),

(P) myotubes (Akt MCD: *P<0.0001 Control B vs. I, *P=0.0067 MCD B vs. I, *P=0.0034 Control I vs. MCD I; Akt CHOL: *P<0.0001 Control B vs. I, *P=0.0024 CHOL B vs. I, *P<0.0001 Control I vs. CHOL I; IR MCD: *P<0.0001 Control B vs. I, *P=0.0002 MCD B vs. I; IR CHOL: *P<0.0001 Control B vs. I, *P<0.0001 CHOL B vs. I, *P<0.0001 Control I vs. CHOL I; IRS1 CHOL: *P=0.0456 Control B vs. I, *P=0.0217 Control I vs. CHOL I; GSK MCD: *P<0.0001 Control B vs. I, *P<0.0001 MCD B vs. I, *P=0.0303 Control I vs. MCD I; GSK CHOL: *P<0.0001 Control B vs. I, *P<0.0001 CHOL B vs. I, *P=0.0006 Control I vs. CHOL I) and

(Q) hepatocytes (Akt MCD: *P=0.0013 Control B vs. I, *P=0.0469 MCD B vs. I; Akt CHOL: Insulin effect *P=0.0085; IR MCD: *P=0.0073 Control B vs. I; IR CHOL: *P=0.025 Control B vs. I, *P=0.025 CHOL B vs. I) incubated with MCD conditioned media (orange) or CHOL conditioned media (green) (n=6/group for adipocytes and myotubes, n=3/group for hepatocytes). Data are means ± SEM, * p<0.05 vs. basal or control, as assessed by two-way unpaired t-test (panel A-J) or two-way ANOVA and Bonferroni post-hoc analysis (panel O-Q). For all conditioned media experiments, each data point was obtained using media from a different mouse, e.g. n=6 means that cells were incubated with Control or NASH conditioned media from 6 individual mice. Source data are provided as a Source Data file. Uncropped immunoblotting images are provided at the end of the supplementary section.

Abbreviations: ACOX1, Acyl-CoA oxidase 1; ADIPOQ, adiponectin; B, Basal; ChREBP, Carbohydrate response element binding protein; CLPP, Caseinolytic mitochondrial matrix peptidase proteolytic subunit; EIF2, Eukaryotic initiation factor 2; FA, fatty acid; GSK3, glycogen synthase kinase 3; HNF1a, hepatocyte nuclear factor-1 alpha; HNF4a, hepatocyte nuclear factor-4 alpha; I, Insulin; IFNG, Interferon gamma; IL1, Interleukin 1; IL8, interleukin 8; INSR/IR, Insulin receptor; IRS1, Insulin receptor substrate 1; KDM5A, Lysine demethylase 5A; LIPE, Hormone-sensitive lipase; LPS, Lipopolysaccharide; MAP4K4, Mitogen-activated protein kinase kinase kinase kinase 4; mTOR, Mammalian target of rapamycin; MYCN, MYCN proto-oncogene, bHLH transcription factor; Nrf2, Nuclear factor-erythroid factor 2-related factor 2; PGC1a, Peroxisome proliferator-activated receptor gamma coactivator 1-alpha; PPAR, Peroxisome proliferator-activated receptor; PTEN, Phosphatase and Tensin Homolog deleted on Chromosome 10; RICTOR, Rapamycin-insensitive companion of mammalian target of rapamycin; RXR, Retinoid X receptor; SCAP, Sterol regulatory element binding protein (SREBP) cleavage-activating protein; TAG, triglyceride; TCA, Tricarboxylic acid cycle; XBP1, X-box-binding protein 1

Figure S2

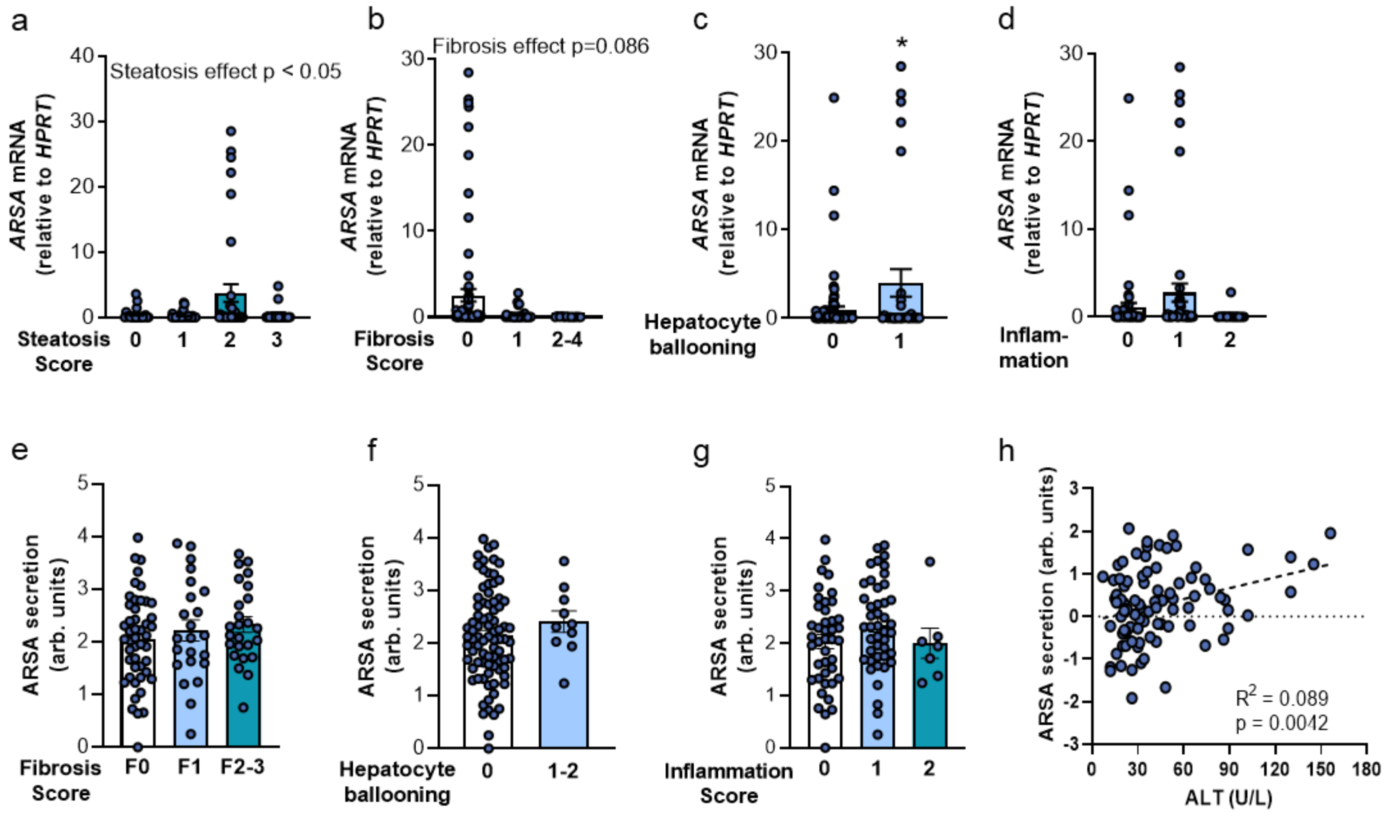

Figure S2. Regulation of ARSA expression and secretion in patients with NASH and type 2 diabetes. Hepatic ARSA mRNA expression and ARSA secretion from precision-cut liver slices was assessed in patients with detailed hepatic histopathological assessment. ARSA mRNA expression in patients grouped by **(A)** steatosis score: score 0 (n=21, white bar), score 1 (n=44, light-blue bar), score 2 (n=38, dark-blue bar), score 3 (n=18, dark-blue bar), steatosis effect *P=0.0045), **(B)** fibrosis score: score 0 (n=82, white bar), score 1 (n=29, light-blue bar), score 2-4 (n=12, dark-blue bar), **(C)** hepatocyte ballooning: score 0 (n=85 white bar), score 1 (n=32; blue bar), *P=0.008, and **(D)** inflammation score: score 0 (n=61, white bar), score 1 (n=50, light-blue bar), score 2 (n=13, dark-blue bar).

Hepatic ARSA secretion in patients grouped by **(E)** fibrosis score: score 0 (n=47, white bar), score 1 (n=23, light-blue bar), score 2-3 (n=25, dark-blue bar), **(F)** hepatocyte ballooning score: score 0 (n=83, white bar), score 1-2 (n=10, blue bar), **(G)** inflammation score: score 0 (n=42, white bar), score 1 (n=44, light-blue bar), score 2 (n=7, dark-blue bar), or **(H)** assessed as correlation with plasma alanine aminotransferase (ALT), n=95. Data are means ± SEM, * p<0.05 vs. no pathology (Score = 0), as assessed by two-way unpaired t-test

(panel C, F) one-way analysis of variance (ANOVA) and Bonferroni post-hoc analysis (panel A, B, D, E, G) or two-tailed non-parametric Spearman correlation (H). Source data are provided as a Source Data file.

Figure S3

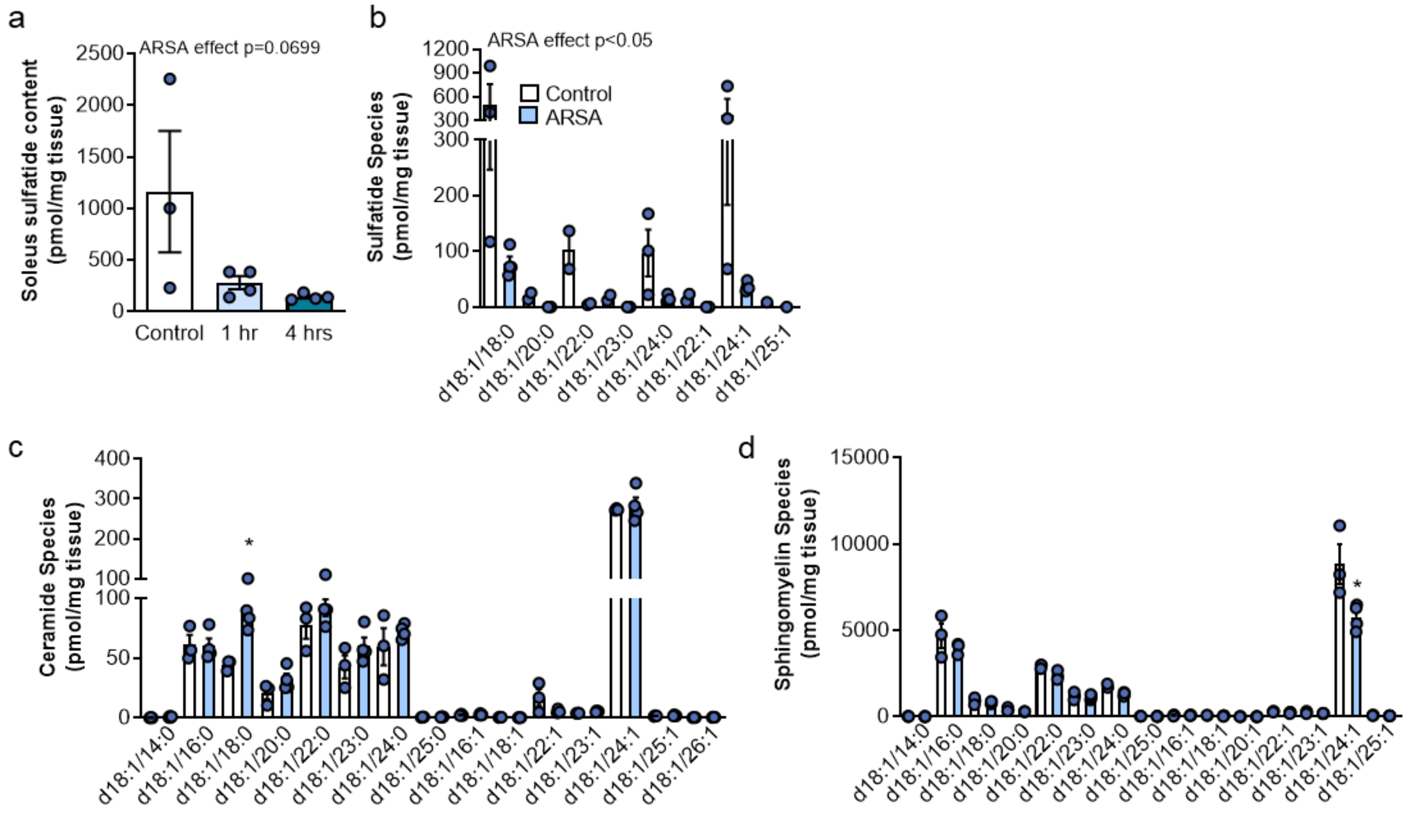

Figure S3. ARSA recombinant protein improves glycaemic control. **(A-D)** Recombinant ARSA was applied to *ex vivo* murine soleus muscle for **(A)** 1 (light-blue bar) and 4 hours (dark-blue bar) or **(B-D)** 4 hours, followed by assessment of **(A)** total sulfatide content (n=3 Control, n=4 1hr, n=4 4hrs; ARSA effect P=0.0699), **(B)** sulfatide species (n=3/group Control 18:0, 24:0, 24:1; n=2/group 20:0, 22:0, 23:0; n=1/group 25:1, n=4/group ARSA 24:0 and 24:1; ARSA effect p=0.0404), **(C)** ceramide species (n=3/group Control, n=4/group ARSA; *P=0.0019 d18:1/18:0) and **(D)** sphingomyelin species (n=3/group Control, n=4/group ARSA; *P=0.034 d18:1/24:1). Data are means ± SEM, * p<0.05 vs. Control, as assessed by two-way unpaired t-test (panel B-D) or one-way analysis of variance (ANOVA) and Bonferroni post-hoc analysis (panel A). Source data are provided as a Source Data file.

Figure S4

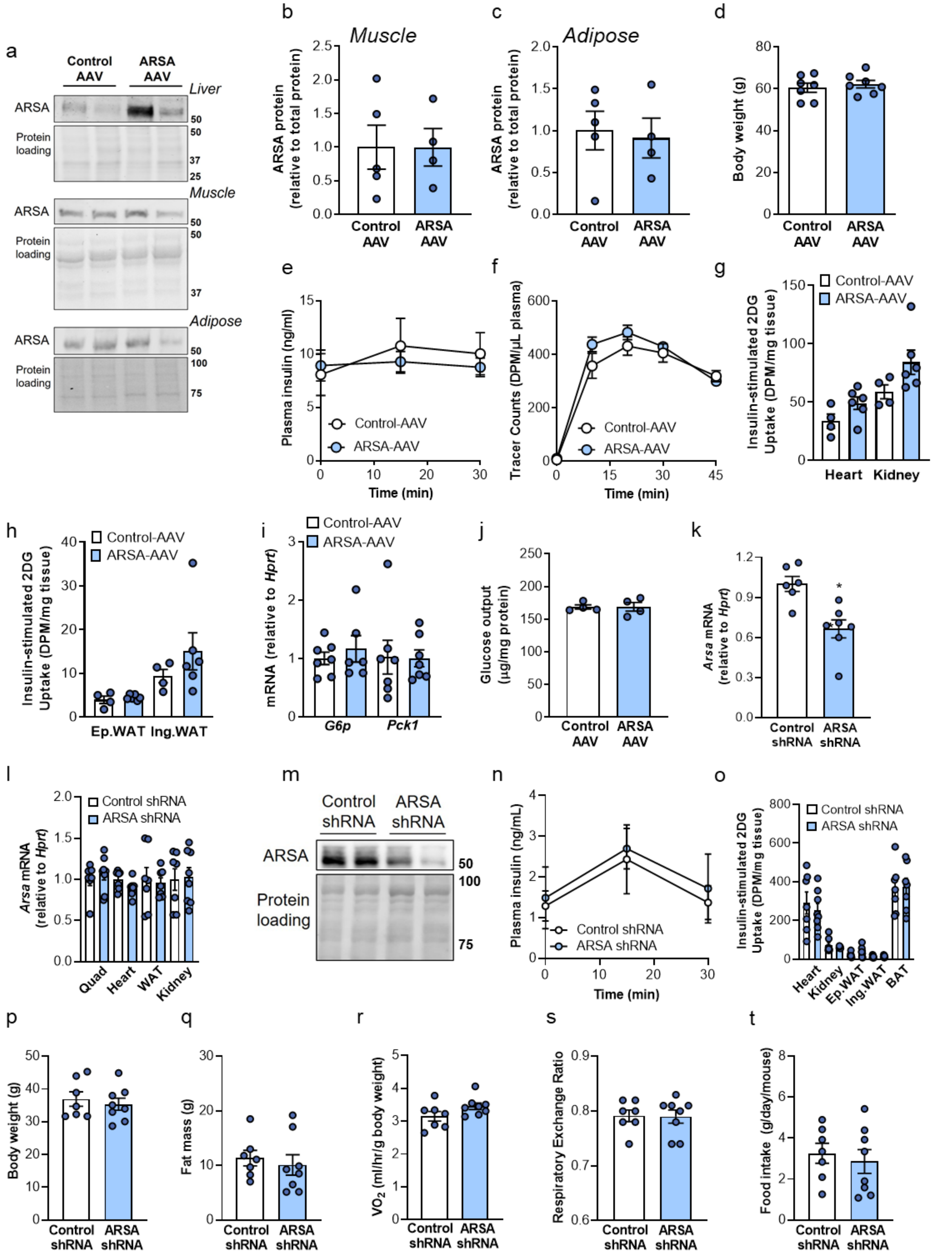
a
Control AAV
ARSA AAV
Liver
ARSA
Protein loading
50
37
25
Muscle
Adipose
100
75
b
Muscle
ARSA protein (relative to total protein)
Control AAV
ARSA AAV
c
Adipose
d
Body weight (g)
e
Plasma insulin (ng/ml)
Time (min)
Control-AAV
ARSA-AAV
f
Tracer Counts (DPM/μL plasma)
g
Insulin-stimulated 2DG Uptake (DPM/mg tissue)
Heart
Kidney
h
Ep.WAT
Ing.WAT
i
mRNA (relative to Hprt)
G6p
Pck1
j
Glucose output (μg/mg protein)
k
Arsa mRNA (relative to Hprt)
Control shRNA
ARSA shRNA
*
l
Quad
Heart
WAT
Kidney
m
n
Plasma insulin (ng/mL)
o
Ep.WAT
Ing.WAT
BAT
p
q
Fat mass (g)
r
VO2 (ml/hr/g body weight)
s
Respiratory Exchange Ratio
t
Food intake (g/day/mouse)

Figure S4. Hepatic ARSA overexpression improves glycaemic control while hepatic ARSA knockdown has opposite effects. ARSA was overexpressed in livers of type 2 diabetic db/db mice using adeno-associated virus (AAV; 1x 10^{12}GC/mouse, blue bars/line graphs) and metabolic assessment carried out 8 weeks following AAV administration. **(A)** Representative immunoblotting analysis of ARSA in liver, quadriceps muscle and adipose tissue, as well as the respective quantification for **(B)** muscle (n=5 Control, n=4 ARSA) and **(C)** adipose tissue (n=5 Control, n=4 ARSA). **(D)** body weight (n=7/group), **(E)** plasma insulin during the glucose tolerance test (n=7/group), **(F)** plasma 1-4C-2-deoxyglucose appearance following ip. injection (n=10/group), and deoxyglucose uptake into **(G)** heart, kidney (n=4/group Control, n=6/group ARSA), and **(H)** epidydimal and inguinal adipose tissue (n=4/group Control, n=6/group ARSA) assessed 45min following ip. injection with 2U/kg insulin and 10µCi 1-^{14}C-2-deoxyglucose/mouse. **(I)** Hepatic mRNA expression of G6p (n=7 Control, n=6 ARSA) and Pck1 (n=7/group), and **(J)** liver slice glucose output (n=4/group). **(K-T)** Hepatic ARSA was knocked down in lean C57BL/6 mice using an AAV-mediated shRNA approach; **(K)** hepatic Arsa mRNA (n=6 Control, n=7 ARSA; *P=0.0032), **(L)** Arsa mRNA in quadriceps muscle, heart, epidydimal adipose tissue (WAT) and kidney (n=7/group Control, n=8/group ARSA), **(M)** representative immunoblotting of hepatic ARSA protein, **(N)** plasma insulin during glucose tolerance test (n=3 Control, n=4 ARSA), **(O)** deoxyglucose uptake into heart (n=7 Control, n=8 ARSA), kidney (n=7/group), epididymal adipose tissue (n=6 Control, n=8 ARSA), inguinal adipose tissue (n=7/group) and brown adipose tissue (n=7/group), **(P)** body weight, **(Q)** fat mass, **(R)** energy expenditure, **(S)** respiratory exchange ratio, and **(T)** food intake (n=7/group Control, n=8/group ARSA). Data are means ± SEM, * $p<0.05$ vs. Control AAV, as assessed by two-way unpaired t-test (panel B-D, G-L, O-T), or two-way ANOVA and Bonferroni post-hoc analysis (panel E, F, N). Source data are provided as a Source Data file. Uncropped immunoblotting images are provided at the end of the supplementary section. Abbreviations: 2DG, 2-deoxy-glucose; BAT, brown adipose tissue; Ep.WAT, epididymal white adipose tissue; G6P, glucose-6-phosphatase; Hprt, Hypoxanthine phosphoribosyltransferase 1; Ing.WAT, Inguinal white adipose tissue; Pck1, phosphoenolpyruvate carboxykinase 1; WAT, white adipose tissue.

Figure S5

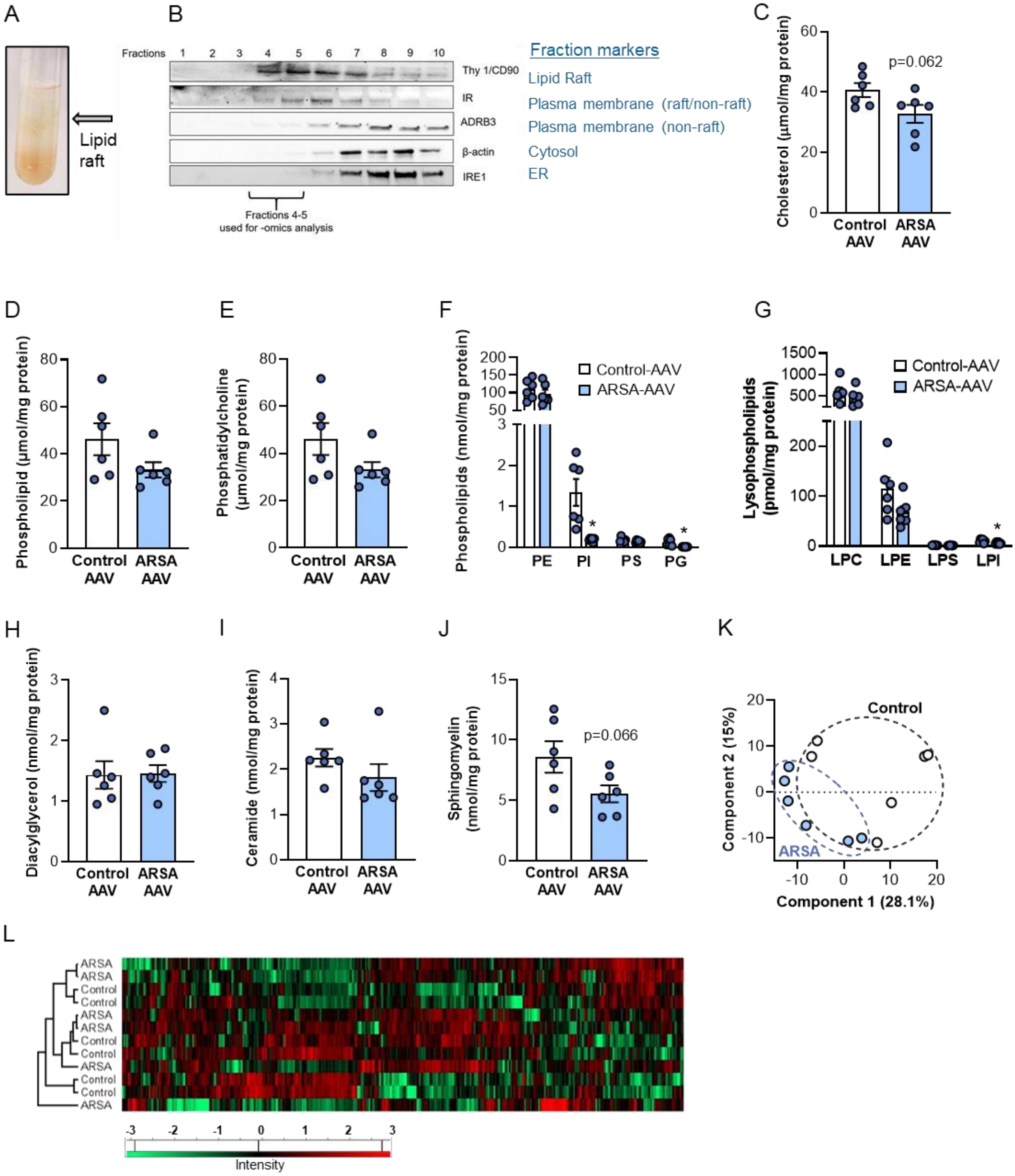

Figure S5. Hepatic ARSA does not modulate skeletal muscle lipid rafts. **(A)** Representative quadriceps muscle lipid raft fractionation and **(B)** representative immunoblotting analysis of all 10 quadriceps muscle

lipid raft fractions, showing purity of the lipid raft-enriched fraction. Similar results were obtained in three samples/group. Quadriceps muscle lipid raft composition, shown as lipid raft-localized **(C)** cholesterol, **(D)** total phospholipid content, **(E)** total phosphatidylcholine content, **(F)** phospholipid classes (*P=0.0055 PI, *P=0.0078 PG), **(G)** lysophospholipid classes (*P=0.038 LPI), **(H)** total diacylglycerol, **(I)** total ceramide and **(J)** total sphingomyelin (n=6/group) in muscle of Control AAV (white bars) and ARSA AAV (blue bars) mice. **(K)** Principal component analysis (PCA) and **(L)** heat map of the muscle lipid raft proteome. Data are means ± SEM, * p<0.05 vs. Control AAV, as assessed by two-way unpaired t-test (panel C-J). Source data are provided as a Source Data file. Uncropped immunoblotting images are provided at the end of the supplementary section. Abbreviations: ACC, acetyl-CoA carboxylase; ADRB3, Adrenoceptor beta 3; ER, endoplasmic reticulum; IR, insulin receptor; IRE1, inositol-requiring enzyme 1; Thy1, Thy-1 Cell Surface Antigen.

Figure S6

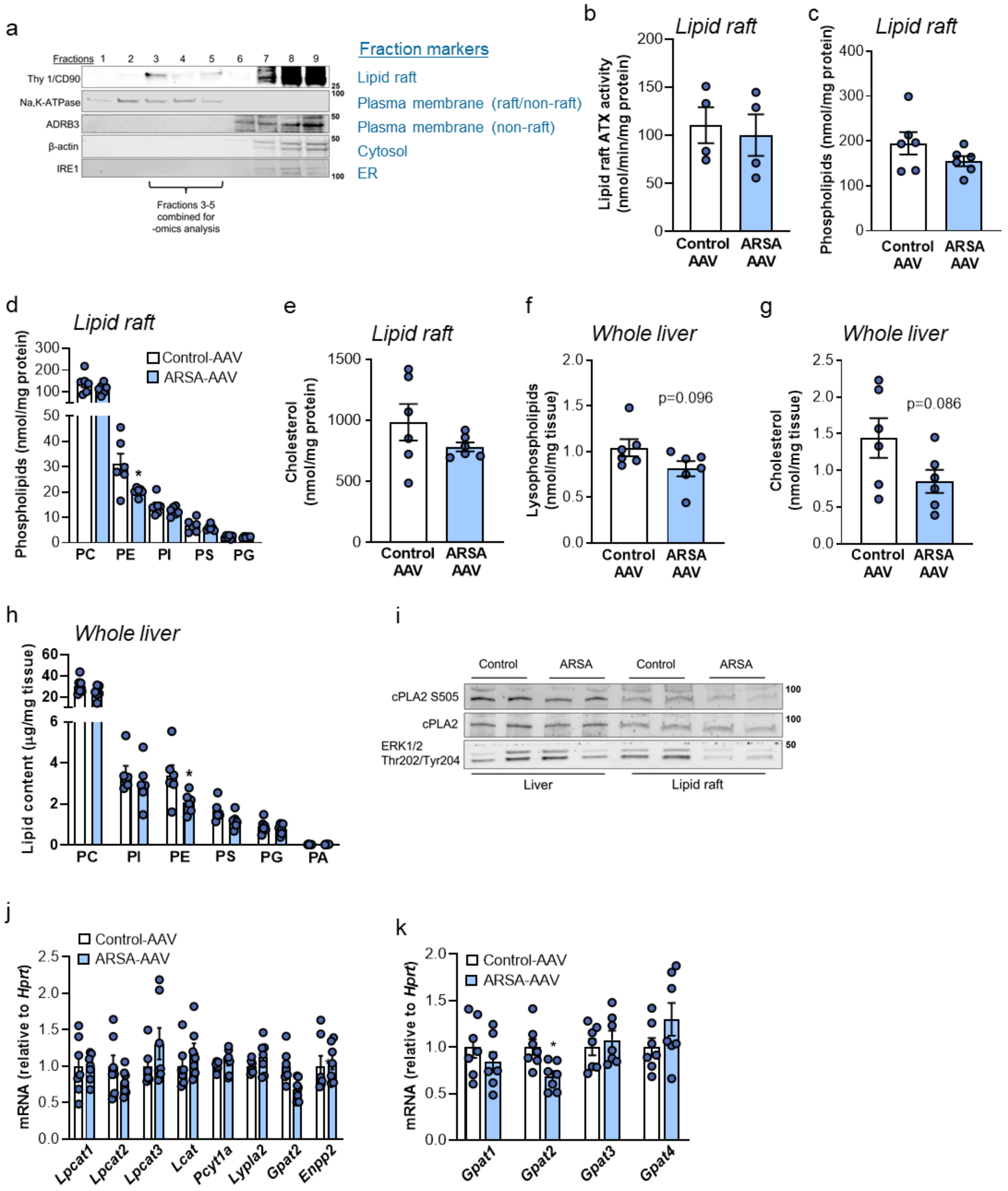

Figure S6. **Hepatic ARSA remodels lipid rafts in the liver**. AAV-mediated overexpression of ARSA in livers of type 2 diabetic db/db mice (Control AAV – white bars; ARSA AAV- blue bars). **(A)** Representative

lipid raft fractionation in the liver. Similar results were obtained in three samples/group. Hepatic lipid raft **(B)** autotaxin activity (n=4/group), **(C)** total phospholipid content (n=6/group), **(D)** phospholipid classes (n=6/group; *P=0.028 PE), and **(E)** cholesterol content (n=6/group). Whole liver **(F)** total lysophospholipid (n=6/group), **(G)** total cholesterol (n=6/group) and **(H)** phospholipid classes (n=6/group; *P=0.041 PE). **(I)** Representative immunoblot of phospholipase A2 (PLA2) S505 and ERK 1/2 Thr202/Tyr204 in whole liver and hepatic lipid raft fractions. Similar results were obtained in four samples/group (as quantified in Figure G). **(J)** hepatic mRNA expression of enzymes involved in lysophospholipid metabolism (n=7/group), **(K)** hepatic mRNA expression of glycerol-3-phosphate acyltransferase (GPAT) isoforms 1-4 (n=7/group; *P=0.009 GPAT2). Data are means ± SEM, * p<0.05 vs. Control AAV, as assessed by two-way unpaired t-test. Source data are provided as a Source Data file. Uncropped immunoblotting images are provided at the end of the supplementary section.

Abbreviations: Adrenoceptor beta 3; ENPP2, Ectonucleotide Pyrophosphatase/Phosphodiesterase 2; ER, endoplasmic reticulum; IRE1, inositol-requiring enzyme 1; LCAT, Lecithin–cholesterol acyltransferase; LPCAT, Lysophosphatidyl choline acyltransferase; LYPLA2, Acyl-protein thioesterase 2; PA, phosphatidic acid; PC, Phosphatidylcholine; PCYT1A, Choline-phosphate cytidylyltransferase A; PE, Phosphatidylethanolamine; PG, Phosphatidylglycerol; PI, Phosphatidylinositol; PLA2, phospholipase A2; PS, Phosphatidylserine, Thy1, Thy-1 Cell Surface Antigen.

Figure S7

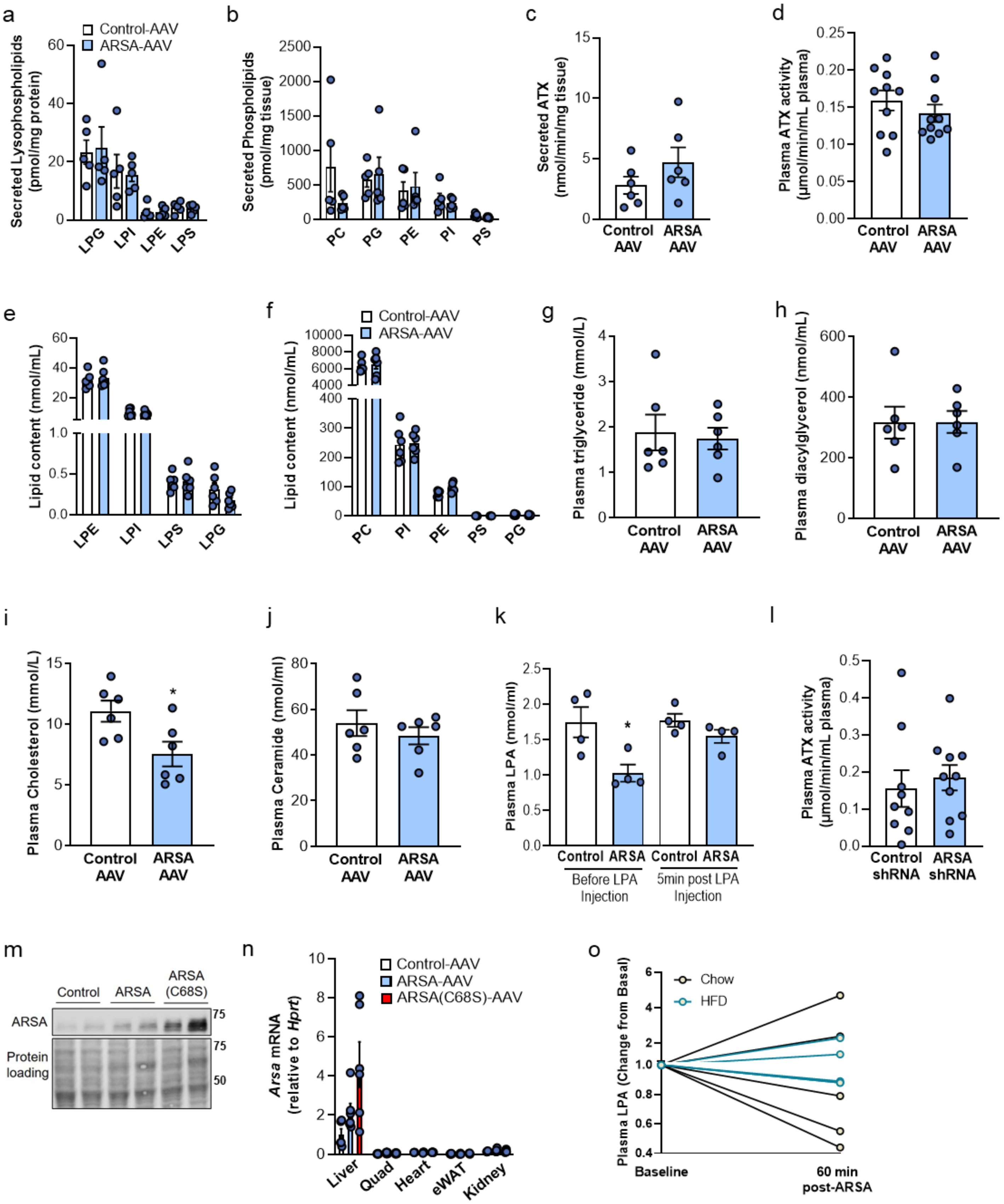
a
Control-AAV
ARSA-AAV
Secreted Lysophospholipids (pmol/mg protein)
LPG
LPI
LPE
LPS
b
Secreted Phospholipids (pmol/mg tissue)
PC
PG
PE
PI
PS
c
Secreted ATX (nmol/min/mg tissue)
Control AAV
ARSA AAV
d
Plasma ATX activity (μmol/min/mL plasma)
e
Lipid content (nmol/mL)
f
g
Plasma triglyceride (mmol/L)
h
Plasma diacylglycerol (nmol/mL)
i
Plasma Cholesterol (mmol/L)
j
Plasma Ceramide (nmol/ml)
k
Plasma LPA (nmol/ml)
Control
ARSA
Before LPA Injection
5min post LPA Injection
l
Control shRNA
ARSA shRNA
m
Control
ARSA
ARSA (C68S)
Protein loading
n
ARSA(C68S)-AAV
Arsa mRNA (relative to Hprt)
Liver
Quad
Heart
eWAT
Kidney
o
Chow
HFD
Plasma LPA (Change from Basal)
Baseline
60 min post-ARSA

Figure S7. ARSA's capacity for sulfatide degradation is required for its actions on hepatic lipid secretion. Assessment of hepatic secretion from precision-cut liver slices obtained from livers of Control AAV (white bars) and ARSA-AAV (blue bars) mice, showing secretion of **(A)** lysophospholipid classes (n=5/group), **(B)** phospholipid classes (n=5/group), and **(C)** secretion of autotaxin (n=6/group). Plasma content of **(D)** autotaxin (n=10/group), **(E)** lysophospholipid classes (n=6/group), **(F)** phospholipid classes (n=6/group), **(G)** total triglyceride (n=6/group), **(H)** total diacylglycerol (n=6/group), **(I)** and cholesterol (n=6/group; *P=0.025) **(J)** total plasma ceramide (n=6/group). **(K)** Plasma lysophosphatidic acid (LPA) in Control-AAV and ARSA-AAV db/db mice before (*P=0.026) and 5min following a 200ng LPA injection (ARSA-AAV mice received 200ng LPA; Control-AAV mice received saline) (n=4/group). **(L)** Plasma autotaxin in Control shRNA and ARSA shRNA mice (n=9 Control, n=10 ARSA). **(M)** Representative immunoblotting of hepatic ARSA protein and **(N)** ARSA mRNA expression in liver, quadriceps muscle, heart, adipose tissue and kidney, of Control (white bars), ARSA-AAV (blue bars) and ARSA(C68S)-AAV (red bars) mice (n=5/group Control, n=6/group ARSA and ARSA(C68S)). **(O)** Changes in plasma LPA in chow and high-fat diet mice at baseline and 60 minutes following ARSA recombinant protein injections (60 min post-ARSA) (n=5/group). Data are means ± SEM, * $p<0.05$ vs. Control AAV, as assessed by two-way unpaired t-test. Source data are provided as a Source Data file. Uncropped immunoblotting images are provided at the end of the supplementary section.

Abbreviations: ATX, autotaxin; eWAT, epididymal adipose tissue; LPA, lysophosphatidic acid; LPC, lysophosphatidylcholine; LPE. lysophosphatidyl ethanolamine; LPG, lysophosphatidyl glycerol; LPI, lysophosphatidyl inositol; LPS, lysophosphatidyl serine; PC, Phosphatidylcholine; PE, Phosphatidyl ethanolamine; PG, Phosphatidylglycerol; PI, Phosphatidyl inositol; PS, Phosphatidyl serine.

2. Supplementary Tables

Table S1. Liver pathology of mice fed the methionine choline deficient (MCD) diet and the diet enriched in fat, fructose and cholesterol (CHOL), as well as their respective Control groups.

	MCD		CHOL	
	**Control**	**NASH**	**Control**	**NASH**
**NAS**		* (P=0.0014)		* (P<0.0001)
≤ 4	3	0	4	0
≥ 5	0	3	0	4
**Steatosis grade (%)**				* (P=0.0001)
0 – $\leq$ 5%	1	0	2	0
1 – 5-33%	0	0	2	0
2 – 34-66%	2	1	0	0
3 – > 66%	0	2	0	4
**Lobular Inflammation**		* (P=0.0474)		* (P=0.0401)
0 – none	2	0	0	0
1 – < 2	1	1	4	1
2 – 2-4	0	2	0	1
3 – > 4	0	0	0	2
**Ballooning**				* (P=0.0025)
0 – none	2	0	1	0
1 – few	1	2	3	0
2 – many	0	1	0	4
**Fibrosis**				
0 – none	2	0	4	2
1a	1	1	0	2
1b	0	0	0	0
1c	0	0	0	0
2	0	1	0	0
3 – bridging	0	1	0	0

Statistical significance was as assessed by unpaired two-tailed t-test between respective Control and NASH diets, with * $p<0.05$.

Table S2. Tissue weights (g) in mice fed a methionine-choline-deficient diet (MCD) or a high-fat high-cholesterol high-fructose diet, as well as in their respective control groups.

	**Control**	**NASH**	**p-value**
1. MCD Diet			
Epididymal adipose tissue	1.49 ± 0.18	0.66 ± 0.03 *	0.0034
Inguinal adipose tissue	0.73 ± 0.13	0.33 ± 0.03 *	0.0216
Brown adipose tissue	0.12 ± 0.01	0.07 ± 0.11 *	0.0196
Quadriceps muscle	0.43 ± 0.01	0.38 ± 0.01 *	0.0478
Heart	0.13 ± 0.08	0.12 ± 0.02	n.s.
2. Cholesterol Diet			
Epididymal adipose tissue	1.96 ± 0.25	2.23 ± 0.11	n.s.
Inguinal adipose tissue	1.02 ± 0.14	2.63 ± 0.16 *	0.0003
Brown adipose tissue	0.18 ± 0.02	0.23 ± 0.02	n.s.
Quadriceps muscle	0.40 ± 0.02	0.38 ± 0.01	n.s.
Heart	0.16 ± 0.01	0.14 ± 0.01	n.s.

Shown are means ± SEM, n = 4 per group. * $p < 0.05$ as assessed by unpaired two-tailed students t-test.

Table S3. Plasma metabolites and cytokines in mice fed a methionine-choline-deficient diet (MCD) or a high-fat high-cholesterol high-fructose diet, as well as in their respective control groups.

	**Control**	**NASH**	**p-value**
1. MCD Diet			
NEFA (mmol/L)	1.25 ± 0.20	0.92 ± 0.18	n.s.
TAG (mmol/L)	1.31 ± 0.16	0.81 ± 0.11 *	0.0466
Cholesterol (mg/mL)	11.16 ± 4.84	8.42 ± 2.63	n.s.
β-OH-butyrate (mmol/L)	0.76 ± 0.09	0.63 ± 0.10	n.s.
TNFα (pg/mL)	2.91 ± 1.28	3.59 ± 1.34	n.s.
IL6 (pg/mL)	552.5 ± 13.1	505.0 ± 11.5 *	0.0342
2. Cholesterol Diet			
NEFA (mmol/L)	1.55 ± 0.13	0.96 ± 0.15 *	0.0254
TAG (mmol/L)	1.09 ± 0.12	1.01 ± 0.21	n.s.
Cholesterol (mg/mL)	14.24 ± 1.32	31.58 ± 2.51 *	0.0009
β-OH-butyrate (mmol/L)	0.75 ± 0.10	0.75 ± 0.11	n.s.
TNFα (pg/mL)	3.59 ± 1.33	3.28 ± 2.26	n.s.
IL6 (pg/mL)	504.0 ± 18.1	527.0 ± 13.6	n.s.

Shown are means ± SEM, n = 4 per group. * $p < 0.05$ as assessed by unpaired two-tailed students t-test. ALT alanine amino transferase, AST aspartate aminotransferase, IL6 Interleukin 6, NEFA non-esterified fatty acid, TAG triglyceride, TNFα tumor necrosis factor α.

Table S4. Clinical and biochemical characteristics of subjects.

	No NAFLD (n=17)	NAFL (n=66)	NASH (n=38)	*p-value*
Males (n, %)	4 (20.0%)	17 (25.7%)	13 (34.2%)	0.416*
BMI (kg/m^2)	46.3 (12.5)	46.1 (7.9)	47.5 (10.1)	0.872^
Age (years)	41.2 (11.7)	45.1 (12.0)	45.7 (11.4)	0.374^
Patients with T2DM (n, %)	3 (15.0%)	16 (22.9%)	13 (34.2%)	0.231*
**AST (IU/L)**	**22.5 (7.2)**	**31.0 (16.9)** [a]	**44.7 (45.2)** [b]	**<0.001**^
**ALT (IU/L)**	**26.9 (15.9)**	**41.6 (26.2)** [a]	**57.1 (60.3)** [b]	**<0.001**^
GGT (IU/L)	42.0 (42.9)	39.2 (40.9)	43.0 (31.9)	0.192^
ALP (IU/L)	77.3 (23.6)	72.2 (20.9)	71.1 (24.2)	0.375^
Triglyceride (mmol/L)	1.3 (0.6)	1.4 (0.7)	1.6 (0.8)	0.100^
Total cholesterol (mmol/L)	4.3 (1.2)	3.9 (1.0)	4.1 (0.9)	0.130^
HDL (mmol/L)	1.1 (0.3)	1.0 (0.2)	0.9 (0.2	0.054^
LDL (mmol/L)	2.7 (1.0)	2.3 (0.8)	2.5 (0.8)	0.106^
**Fasting blood glucose (mmol/L)**	**5.3 (1.1)**	**5.7 (1.7)**	**6.6 (2.6)** [b,c]	**0.002**^
Hba1c (%)	5.7 (0.5)	6.1 (1.3)	6.4 (1.4)	0.238^
Insulin (mU/L)	10.3 (8.9)	10.5 (12.3)	9.9 (7.4)	0.634^
C-peptide (pmol/L)	890 (753)	852 (436)	990 (490)	0.131^
HOMA-IR	1.4 (1.3)	1.3 (1.5)	1.4 (1.0)	0.494^
Urea (mmol/L)	4.4 (1.7)	4.7 (1.8)	5.1 (2.1)	0.332^
Creatine (µmol/L)	68.0 (15.2)	73.0 (27.3)	73.1 (15.5)	0.270^
eGFR (mL/min/1.73 m²)	78.5 (14.2)	76.6 15.0)	73.2 (13.0)	0.453^
Albumin (g/L)	35.0 (2.9)	36.4 (4.7)	37.0 (2.8)	0.090^
Bilirubin (mmol/L)	10.2 (8.2)	10.8 (6.1)	9.2 (3.6)	0.555^
White Cell count (x10^9)	6.9 (2.6)	7.7 (2.4)	8.1 (2.8)	0.189^
Platelets (x10^9)	241 (81)	243 (51)	246 (65)	0.978#

*Chi-square test, ^Kruskal-Wallis Test with pairwise comparisons (Bonferroni correction), #One-way ANOVA, a p<0.05 no NAFLD vs. NAFL, b p<0.05 no NAFLD vs NASH, c p<0.05 NAFL vs NASH. Numbers in bold are significant. Data are shown as mean ± SD for continuous variables and % for categorial variables. Abbreviations: NAFLD: Non-alcoholic fatty liver disease, NASH: Non-alcoholic steatohepatitis, BMI: Body Mass Index, AST: Aspartate aminotransferase, ALT: Alanine aminotransferase, GGT: Gamma glutamyl transferase, HDL: High Density Lipoprotein, LDL: Low Density Lipoprotein, ALP: Alkaline phosphatase, Hba1c: Haemoglobin A1c, HOMA-IR: Homeostatic Model Assessment of Insulin Resistance and eGFR: Estimated Glomerular Filtration Rate.

Table S5. Liver pathology in human subjects.

	**No NAFLD (n=17)**	**NAFL (n=66)**	**NASH (n=38)**
**Steatosis Grade % (n, %)**			
0 – <5	17 (100%)	1 (1.5%)	0 (0%)
1 – 5–33	0 (0%)	41 (62.1%)	6 (15.8%)
2 – 34–66	0 (0%)	16 (24.2%)	22 (57.9%)
3 – > 66	0 (0%)	8 (12.1%)	10 (26.3%)
**Lobular inflammation (n, %)**			
0 – none	17 (100%)	39 (59.1%)	0 (0%)
1 – <2	0 (0%)	23 (34.8%)	29 (76.3%)
2 – 2–4	0 (0%)	4 (6.1%)	8 (21.1%)
3 – >4	0 (0%)	0 (0%)	1 (2.6%)
**Ballooning (n, %)**			
0 – none	17 (100%)	63 (95.5%)	0 (0%)
1 – few	0 (0%)	2 (3.0%)	32 (84.2%)
2 – many	0 (0%)	1 (1.5%)	6 (15.8%)
**Fibrosis (n, %)**			
0 – none	17 (100%)	48 (72.7%)	12 (31.6%)
1 – Perisinusoidal or periportal	0 (0%)	13 (19.7%)	19 (50.0%)
2 – Perisinusoidal and portal / periportal fibrosis	0 (0%)	5 (7.6%)	5 (13.2%)
3 – Bridging	0 (0%)	1 (1.5%)	1 (2.6%)
4 – Cirrhosis	0 (0%)	0 (0%)	1 (2.6%)

Table S6. Tissue weights and plasma NEFA in ARSA-AAV and Control-AAV mice.

	**Control AAV**	**ARSA AAV**
Tissue weights		
Liver (g)	3.87 ± 0.207	3.53 ± 0.32
Epididymal adipose (g)	3.48 ± 0.29	3.31 ± 0.28
Inguinal adipose (g)	5.27 ± 0.37	5.11 ± 0.40
Kidney (mg)	350.6 ± 23.8	356.0 ± 17.4
Quadriceps (mg)	227.9 ± 13.5	218.2 ± 16.7
Gastrocnemius (mg)	193.5 ± 12.0	188.3 ± 12.5
Heart (mg)	117.5 ± 10.5	126.3 ± 9.4
Plasma analysis		
NEFA (mmol/L)	0.50 ± 0.04	0.71 ± 0.10

Shown are means ± SEM, n = 12/group Control, n=11/group ARSA, n=7/group for kidney, n=6 Control NEFA, n=7 ARSA NEFA. NEFA non-esterified fatty acid

Table S7. Tissue weights and plasma lipids in Control shRNA and ARSA shRNA mice.

	Control shRNA	ARSA shRNA
Tissue weights		
Liver (g)	1.33 ± 0.05	1.32 ± 0.05
Epididymal adipose (g)	1.72 ± 0.22	1.39 ± 0.20
Inguinal adipose (g)	1.04 ± 0.17	0.87 ± 0.15
Kidney (mg)	379.4 ± 25.9	398.8 ± 33.2
Quadriceps (mg)	347.1 ± 19.3	319.7 ± 17.4
Gastrocnemius (mg)	329.9 ± 10.4	337.6 ± 18.9
Heart (mg)	134.8 ± 3.7	140.0 ± 5.0
Plasma analysis		
NEFA (mmol/L)	0.33 ± 0.09	0.27 ± 0.06
TAG (mmol/L)	0.80 ± 0.08	0.84 ± 0.08
Cholesterol (mg/mL)	9.46 ± 0.70	9.65 ± 1.11

Shown are means ± SEM, n = 7/group Control, n=8/group ARSA. NEFA non-esterified fatty acid, TAG triglyceride

Table S8. Primer sequences

Primer	FOR	REV
Acta2	CATCTTTCATTGGGATGGAG	TTAGCATAGAGATCCTTCCTG
ApoB	CTCCTACAAGAATAAGTATGGG	GAAGCGACTGTTGATCTTAG
ARSA (human)	CGGACTGGAAAGTACAAGGCTC	TTGGACAGGTCATAGAGCAGCG
Arsa (mouse)	AAGTCTGTCTTCTTCTACCC	GAGCCTTGTATTTCCCATTC
Col1a1	CGTATCACCAAACTCAGAAG	GAAGCAAAGTTTCCTCCAAG
Ctgf	GAGGAAAACATTAAGAAGGGC	AGAAAGCTCAAACTTGACAG
Enpp2	GGACATTCTTTTGGTCTGTG	TTGTGTCCAGAAAAATCAGG
F4/80	CCTGGACGAATCCTGTGAAG	GGTGGGACCACAGAGAGTTG
G6p	TTCAAGTGGATTCTGTTTGG	AGATAGCAAGAGTAGAAGTGAC
Gpat1	CAACACCATCCCCGACATC	GTGACCTTCGATTATGCGATCA
Gpat2	AGAAGGGATCTTTGAGTGTG	CTAACTGCAGATGAACTGTC
Gpat3	GGAGGATGAAGTGACCCAGA	CCAGTTTTTGAGGCTGCTGT
Gpat4	TGTCTGGTTTGAGCGTTCTG	TTCTGGGAAGATGAGGATGG
HPRT (human)	ATAAGCCAGACTTTGTTGG	ATAGGACTCCAGATGTTTCC
Hprt (mouse)	AGGGATTTGAATCACGTTTG	TTTACTGGCAACATCAACAG
Hsp47	ATGTTCTTTAAGCCACACTG	TCGTCATAGTAGTTGTACAGG
Lcat	TGATGGTTTTATCTCTCTCGG	GCTTTATGTTGGACAGGATG
Lpcat1	GAAAGTGGCCTCAGATAATG	AATTTGTTTGGGTAGCGTAG
Lpcat2	AGATTGAGTTTATGCCTGTG	GGGTATTTCCAATGCTTCAG
Lpcat3	CAGAAGACTATGATAACCGC	TTCATCGAAGCCATTAAAGC
Lypla2	GACACTCAACATGAAGATGG	CAAAGCCTTGATGTTCTCTG
Mttp	TCCTGGACTTTTTGGATTTC	TTGAACTTACTAAGGAGGGC
Pck1	AATATGACAACTGTTGGCTG	AATGCTTTCTCAAAGTCCTC
Pcyt1a	GTCAGCTTTATCAACGAGAAG	TTTTCCTCCACATCTTTCAC
Pdgfb	GTGGGCAGGGTTATTTAATATG	GAGGGGAACAACATTATCAC
Tgfb1	GGATACCAACTATTGCTTCAG	TGTCCAGGCTCCAAATATAG

Table S9. Antibody list.

Protein	Company	Product Code	Dilution
Akt	Cell Signalling	9272S	1:1,000
Akt (S473)	Cell Signalling	4058S	1:1,000
ADRB3	Abcam	ab94506	1:1,000
ARSA	Abcam	ab174844	1:1,000
beta actin	Abcam	ab3280	1:1,000
Erk (T202/Y204)	Cell Signalling	9101S	1:1,000
GSK3α/β	Cell Signalling	5676S	1:1,000
GSK3α/β (S21/9)	Cell Signalling	8566S	1:1,000
IR	Cell Signalling	3020S	1:1,000
IR (T1158/1162/1163)	Upstate	07-841	1:1,000
IRE1	Cell Signalling	3294S	1:1,000
IRS1	Cell Signalling	3407S	1:1,000
IRS1 (Y612)	Sigma	12658	1:1,000
Na,K-ATPase	Cell Signalling	3010S	1:1,000
cPLA2	Santa Cruz	sc-454	1:1,000
cPLA2 (S505)	Abcam	ab53105	1:1,000
Thy1	Cell Signalling	9798	1:1,000

Table S10. Lipid name and ion form used for lipidomics quantification

Abbreviation	Lipid name	Ion form
AcCa	Acyl Carnitine	+H
Cer	Ceramide	+H
CerP	Ceramide phosphate	+H
CerPE	Ceramide phosphoethanolamine	+H
ChE	Cholesterol ester	+NH4
Co	Coenzyme Q	+H
CL	Cardiolipin	-H
DG	Diglyceride	+NH4
Hex1Cer	Hexosyl ceramide	+H
Hex2Cer	Dihexosyl ceramide	+H
Hex3Cer	Trihexosyl ceramide	+H
Hex1SPH	Hexosylsphingosine	+H
LPA	Lyso phosphatidic acid	+H
LPC	Lyso phosphatidylcholine	+H
LPE	Lyso phosphatidylethanolamine	+H
LPG	Lyso phosphatidylglycerol	-H
LPI	Lyso phosphatidylinositol	-H
LPS	Lyso phosphatidylserine	+H
MG	Monoglyceride	+NH4
PA	Phosphatidic acid	-H
PC	Phosphatidylcholine	+H
PE	Phosphatidylethanolamine	+H
PG	Phosphatidylglycerol	-H
PI	Phosphatidylinositol	-H
PS	Phosphatidylserine	+H
SM	Sphingomyelin	+H
SPH	Sphingosine bases	+H
SPHP	Sphingosine phosphate	+H
ST	Sulfatide (galactosyl ceramide sulfate)	+H
TG	Triglyceride	+NH4

Table S11. LC gradient used for secretome identification and quantification.

Retention time (min)	Flow (µL/min)	%B
0	0.25	2.5
5	0.25	5
6	0.25	12.5
114	0.25	32.5
120	0.25	42.5
125	0.25	99
132	0.25	99
133	0.25	2.5
155	0.25	2.5

Table S12. LC gradient used for proteome identification and quantification.

**LC on Orbitrap Elite**	
Time	Solvent B percentage
0	3
6	3
60	25
70	40
75	90
80	90
81	3
90	3
**LC on Orbitrap Eclipse**	
Time	Solvent B percentage
0	3
6	3
65	23
75	40
80	80
85	80
86	3
96	3
**LC on Q Exactive Plus**	
Time	Solvent B percentage
0	2
6	2
95	22
105	40
110	80
115	80
117	2
130	2

Table S13. Details on mass spectrometry platforms utilized in proteomics and lipidomics experiments

**Proteomics**	
NASH hepatocyte secretome (Figure 1)	Q Exactive Plus (ThermoFisher Scientific) coupled to RSLC nano HPLC (Ultimate 3000, UHPLC ThermoFisher Scientific)
NASH hepatocyte intracellular proteome (Figure 1)	Q Exactive Plus Orbitrap (Thermo Fisher Scientific) coupled to RSLC nano HPLC (Ultimate 3000 UHPLC, Thermo Fisher Scientific).
Quadriceps muscle lipid raft composition (Figure 5, Figure S5)	Q Exactive Plus Orbitrap (Thermo Fisher Scientific) coupled to RSLC nano HPLC (Ultimate 3000 UHPLC, Thermo Fisher Scientific).
**Lipidomics**	
Soleus muscle sphingolipid analysis (Figure S3)	TSQ Altis coupled to Vanquish HPLC
Quadriceps muscle sulfatide content (Figure 5)	Orbitrap Fusion Lumos (Thermo Fisher Scientific) coupled to Vanquish UHPLC
Quadriceps muscle lipid raft composition (Figure 5, Figure S5)	Orbitrap Fusion Lumos (Thermo Fisher Scientific) coupled to Vanquish UHPLC
Liver lipidome (Figure 6, Figure S6)	Orbitrap Fusion Lumos (Thermo Fisher Scientific) coupled to Vanquish UHPLC
Liver lipid raft lipidome (Figure 6, Figure S6)	Orbitrap Fusion Lumos (Thermo Fisher Scientific) coupled to Vanquish UHPLC
Liver Slice Secreted lipids (Figure 7, Figure S7)	Orbitrap Fusion Lumos (Thermo Fisher Scientific) coupled to Vanquish UHPLC
Plasma Lipidome (Figure 7, Figure S7)	Orbitrap Fusion Lumos (Thermo Fisher Scientific) coupled to Vanquish UHPLC
Liver sulfatide content (Figure 7)	Orbitrap Fusion Lumos (Thermo Fisher Scientific) coupled to Vanquish UHPLC

3. Uncropped immunoblots (related to supplementary figures only)

Figure S1O: Adipocytes

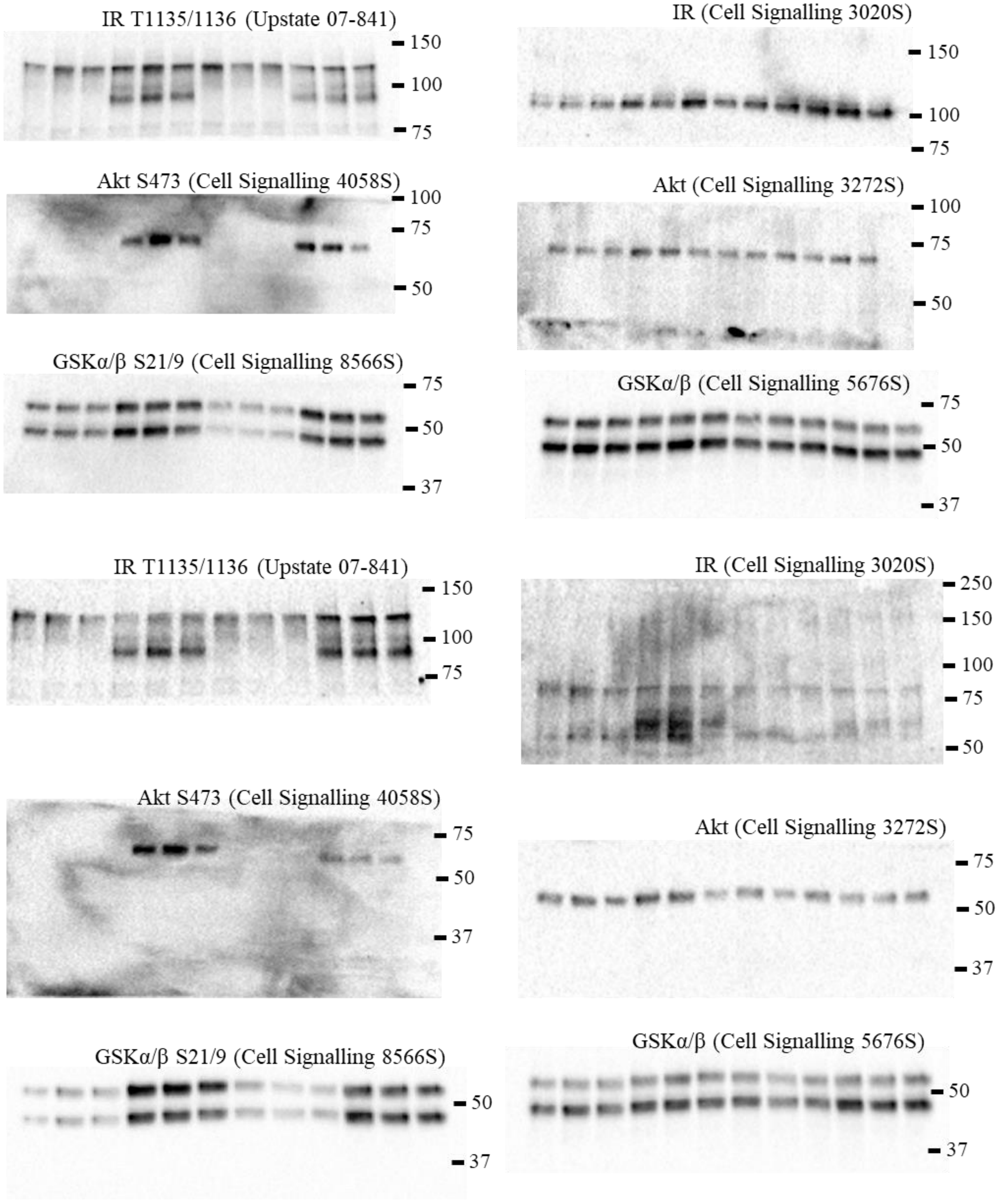

Figure S1P: Myotubes

IR T1135/1136 (Upstate 07-841)

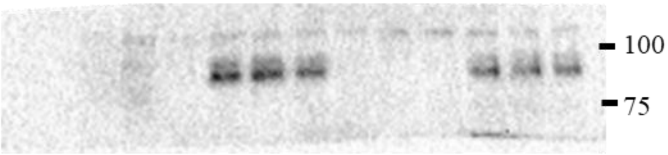

IRS1 (Cell Signalling 3407S)

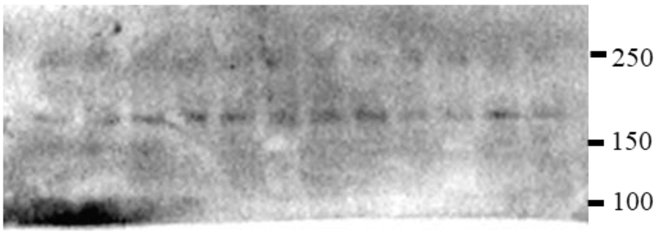

Akt (Cell Signalling 3272S)

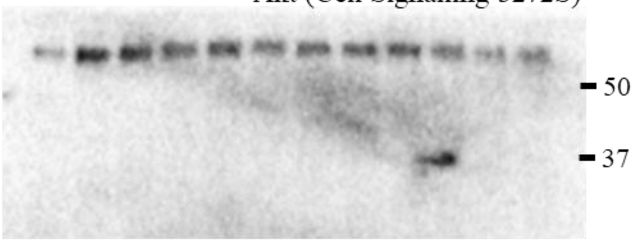

GSKα/β (Cell Signalling 5676S)

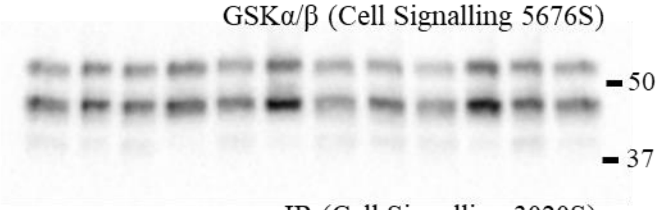

IR (Cell Signalling 3020S)

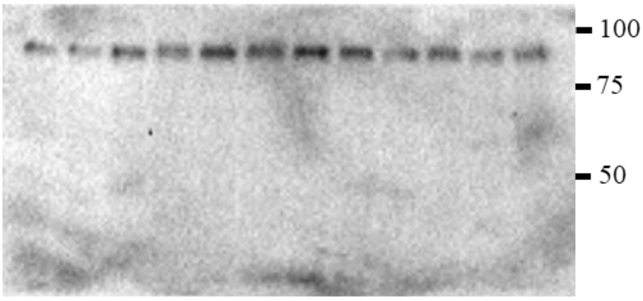

IRS1 (Cell Signalling 3407S)

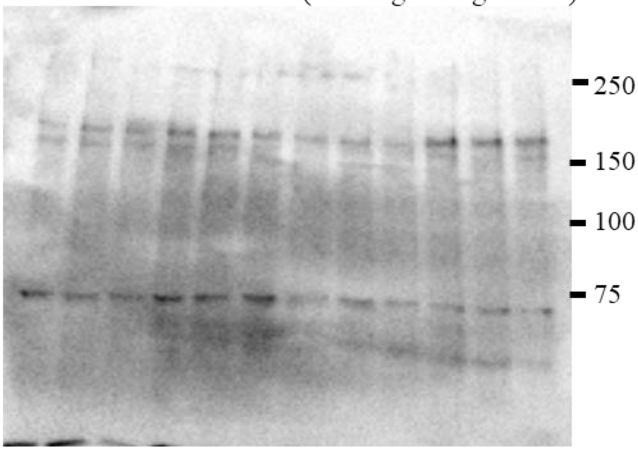

IRS1 Y612 (Sigma 12658)

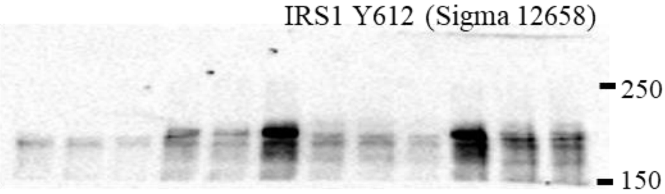

Akt S473 (Cell Signalling 4058S)

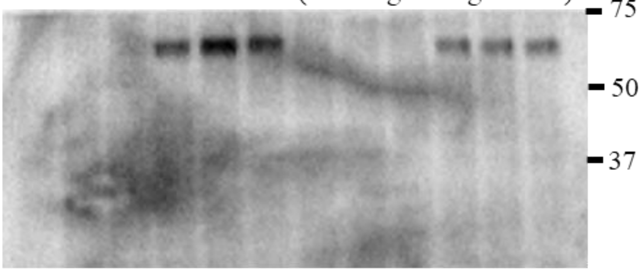

GSKα/β S21/9 (Cell Signalling 8566S)

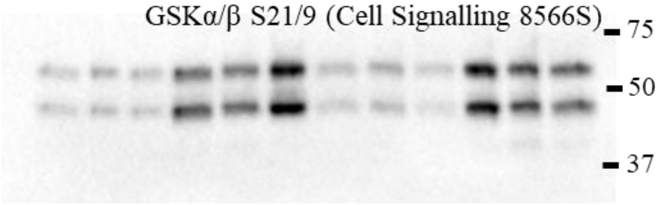

IR T1135/1136 (Upstate 07-841)

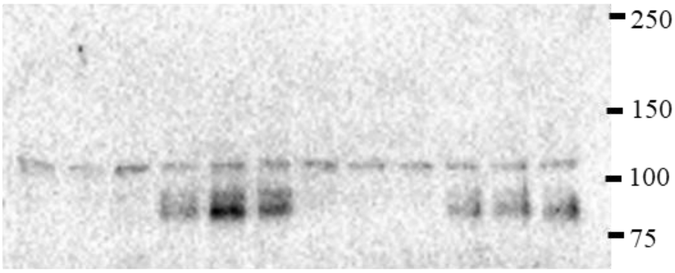

IRS1 Y612 (Sigma 12658)

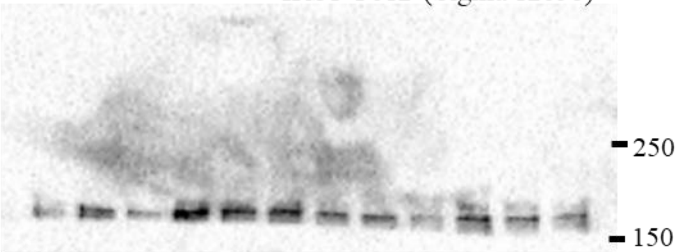

Akt S473 (Cell Signalling 4058S)

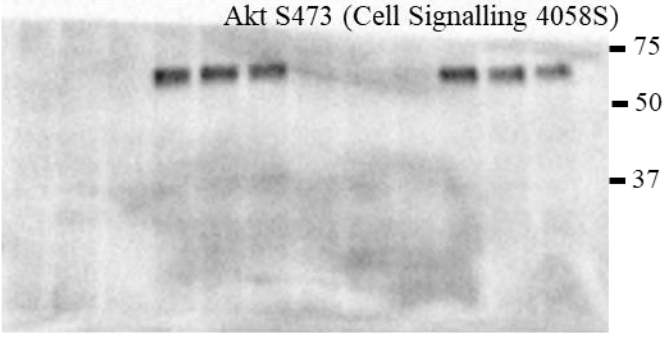

Figure S1P: Myotubes

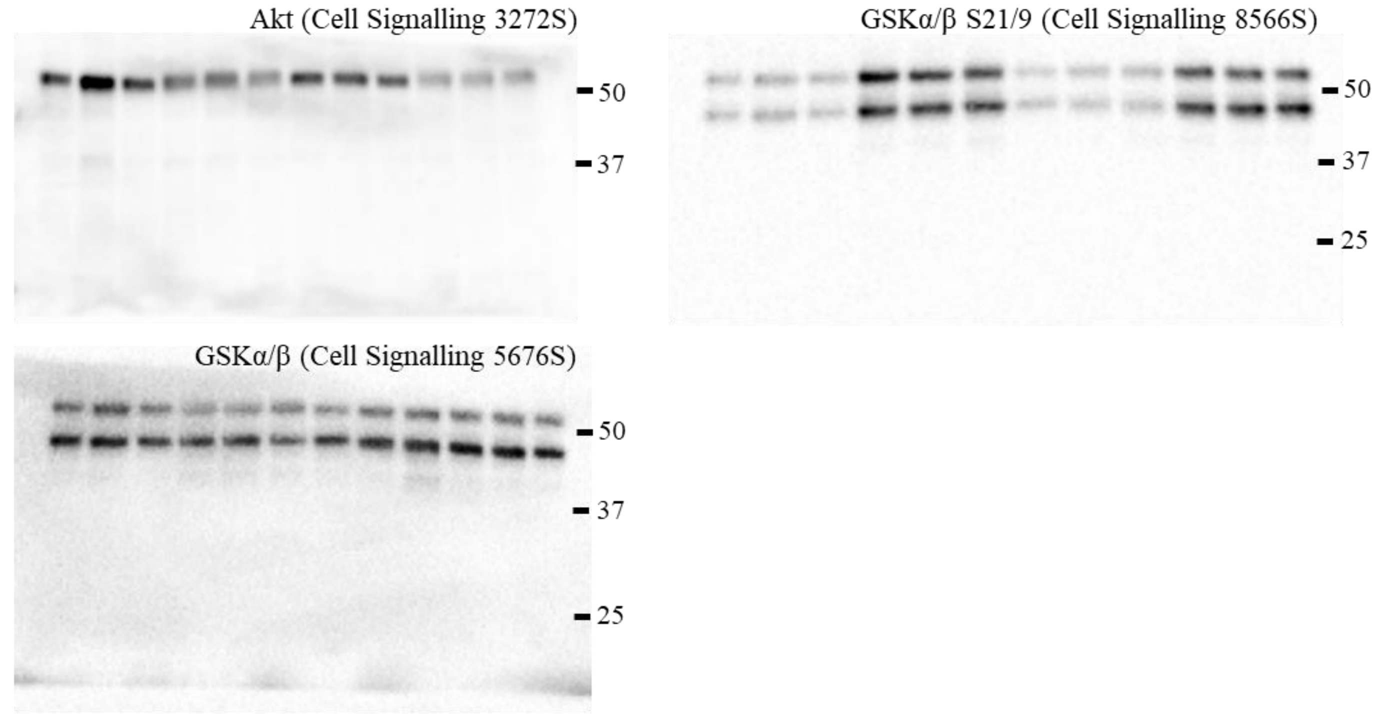

Figure S1Q: Hepatocytes

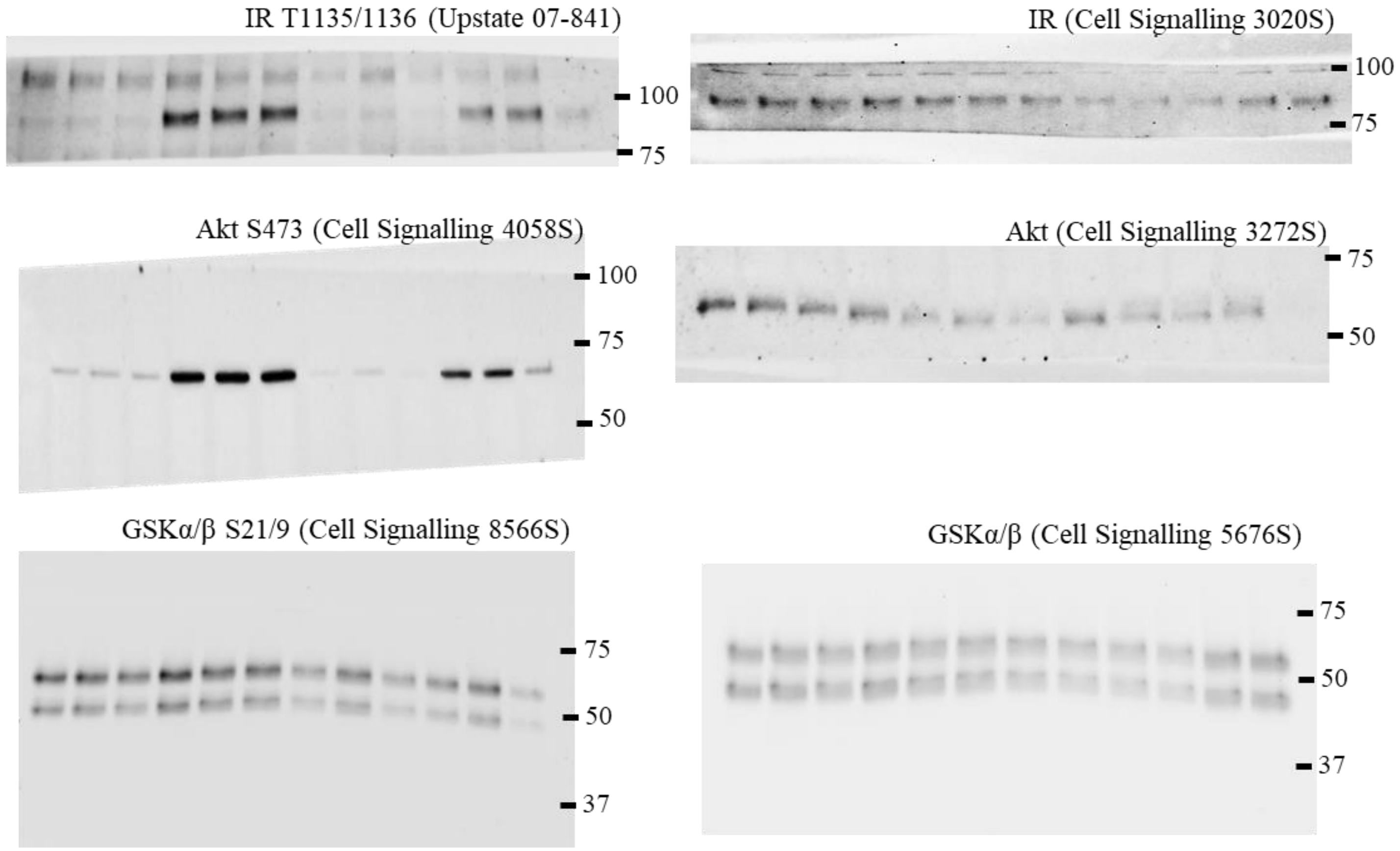

Figure S1Q: Hepatocytes

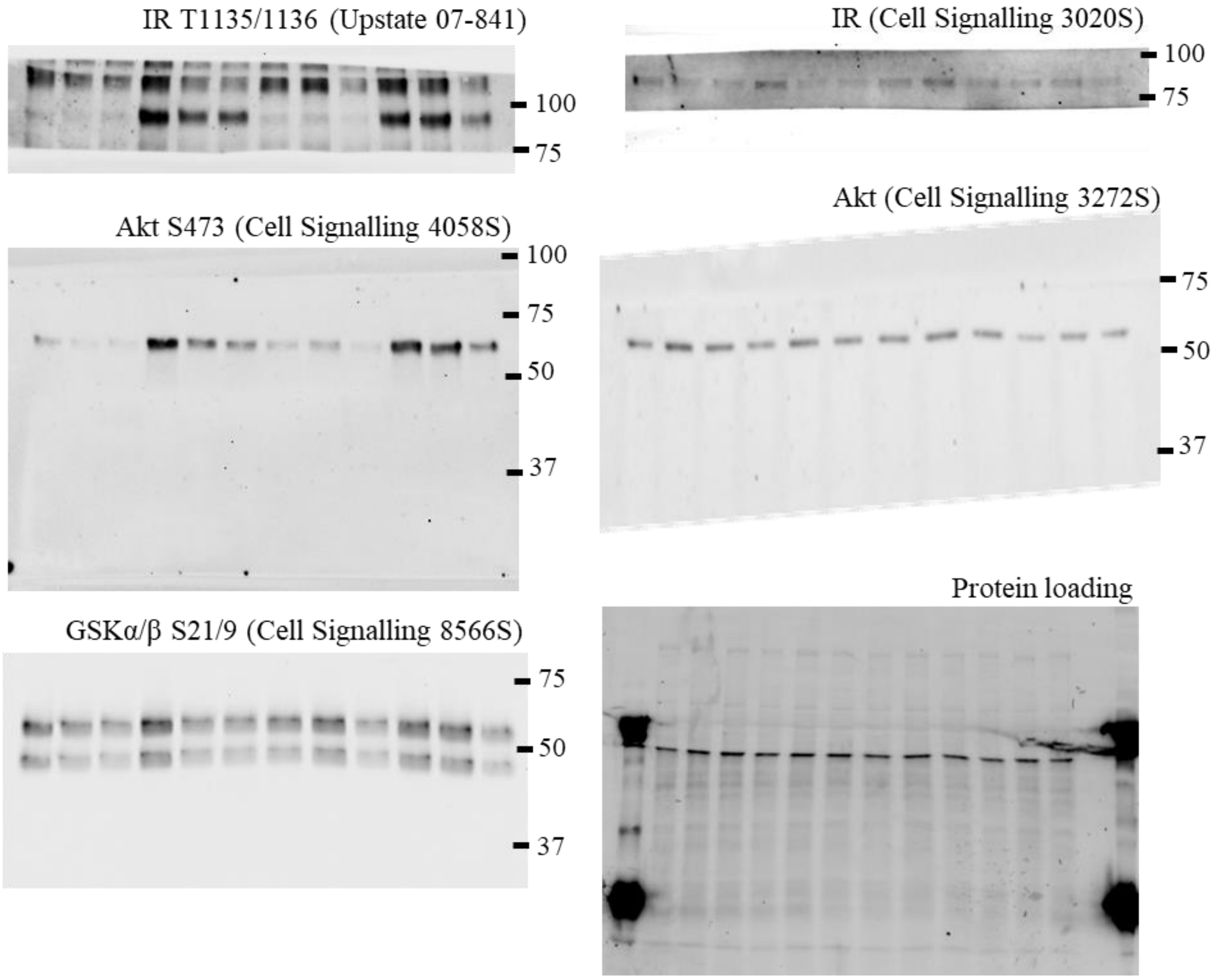

Figure S4A

ARSA (Abcam ab17844)

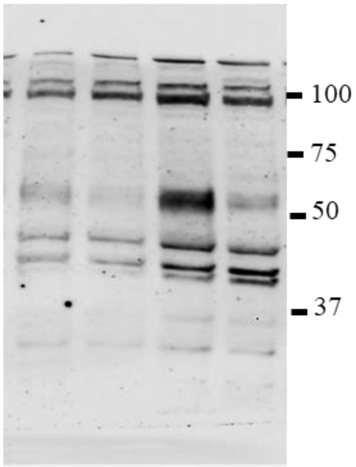

Protein loading liver

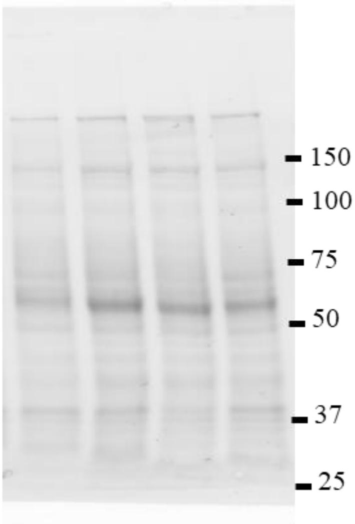

ARSA (Abcam ab17844)

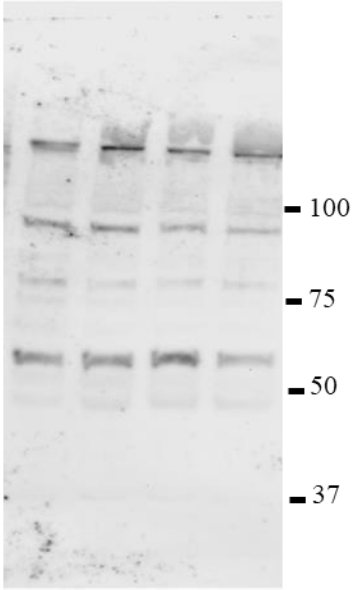

Protein loading muscle

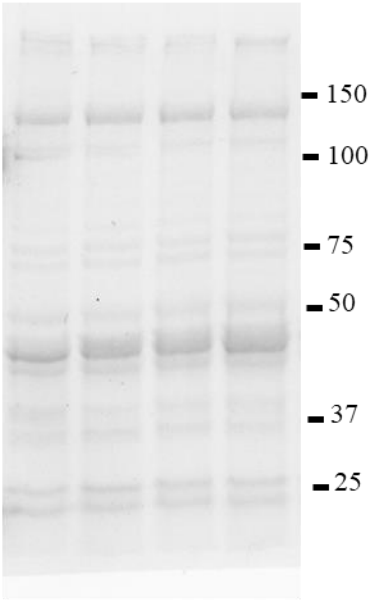

ARSA (Abcam ab17844)

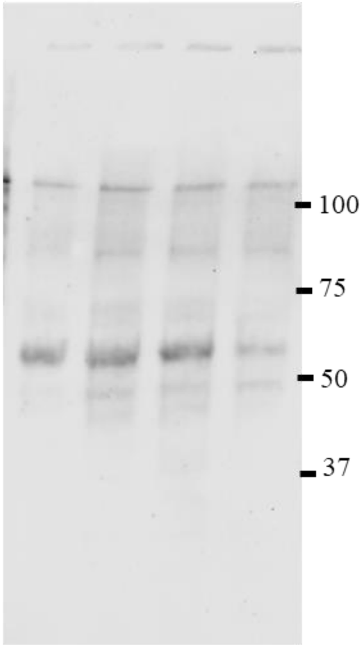

Protein loading adipose

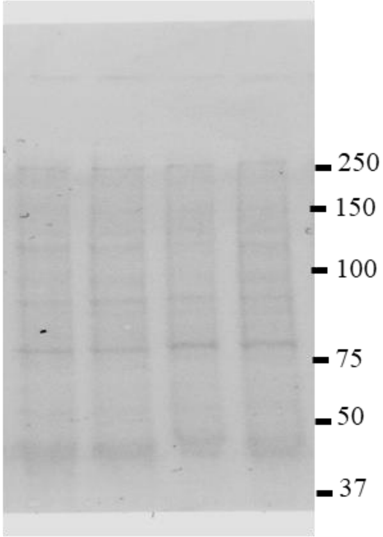

Figure S4O

ARSA (Abcam ab17844)

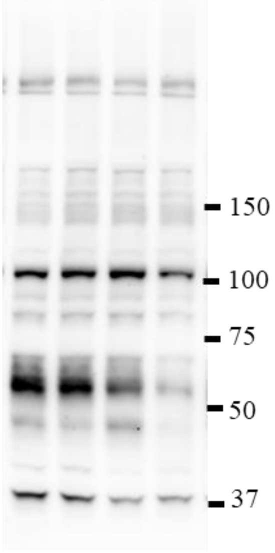

Protein loading

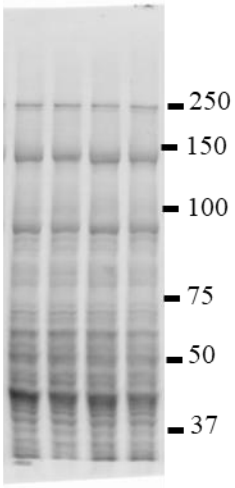

Figure S5B

Thy1 (Cell Signalling 9798)

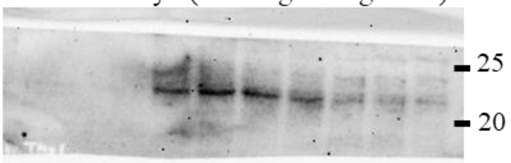

IR (Cell Signalling 3020S)

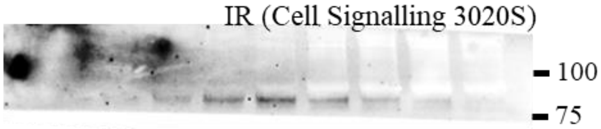

ADRB3 (Abcam ab94506)

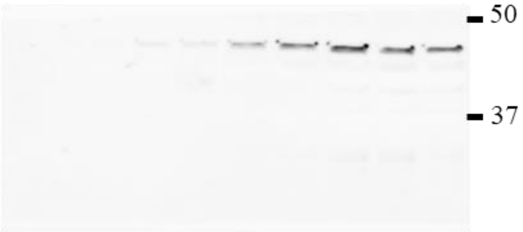

Beta actin (Abcam ab3280)

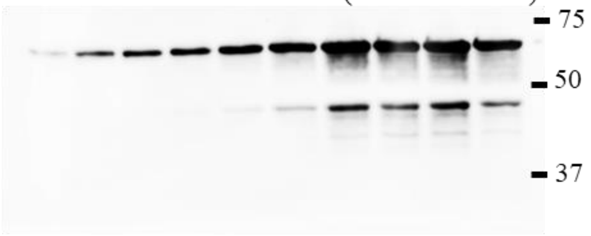

IRE1 (Cell Signalling 3294S)

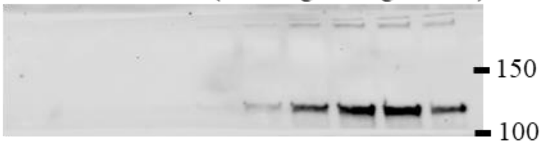

Figure S6A

Thy1 (Cell Signalling 9798)

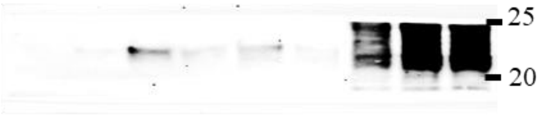

Na,K-ATPase (Cell Signalling 3010S)

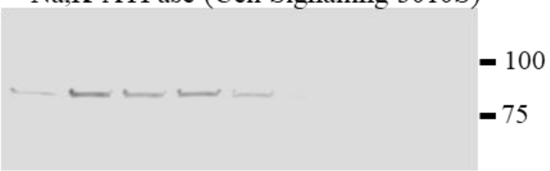

ADRB3 (Abcam ab94506)

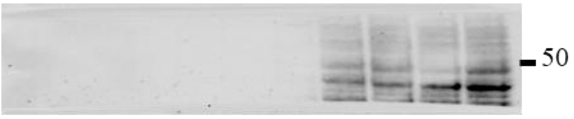

Beta actin (Abcam ab3280)

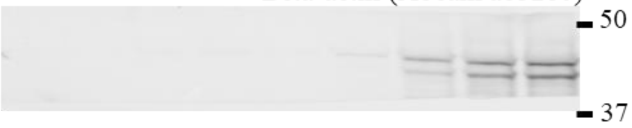

IRE1 (Cell Signalling 3294S)

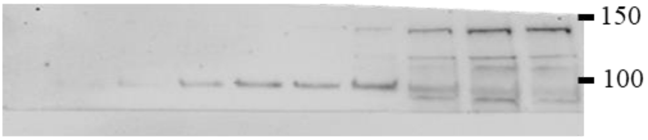

Figure S6I

cPLA2 S505 (Abcam ab53105)

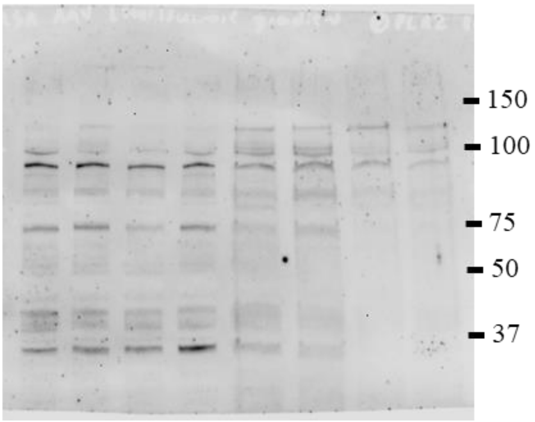

cPLA2 (Santa Cruz sc-454)

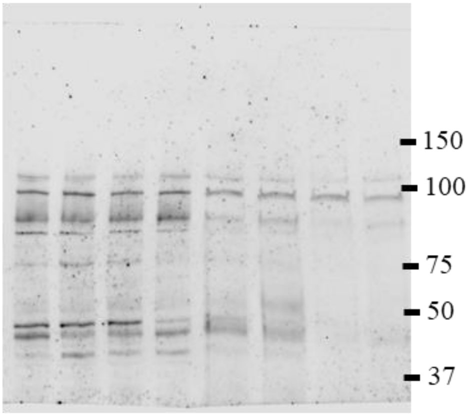

Figure S6I

Erk1/2 Thr202/Tyr204 (Cell Signalling 9101S)

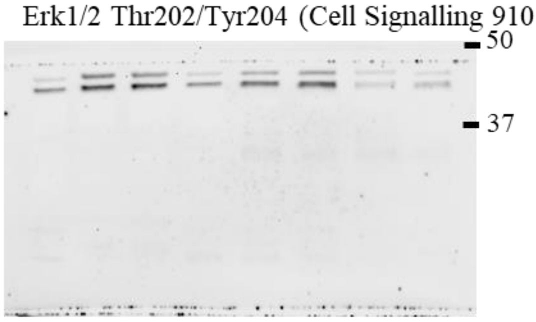

Figure S7O

ARSA (Abcam ab17844)

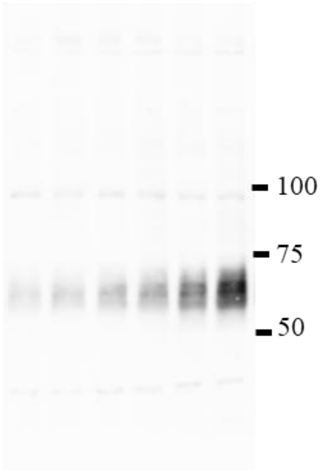

Protein loading

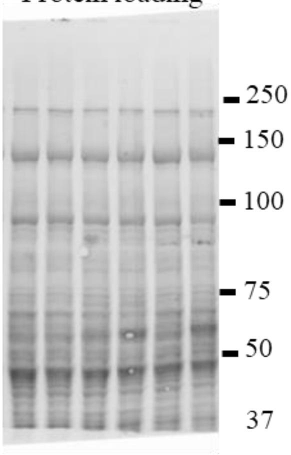

Supplement: Supplementary file 1 — Supplementary Information [file 41467_2022_28889_MOESM1_ESM.pdf]
