# Supplementary material for: Deep proteomic profiling unveils arylsulfatase A as a non-alcoholic steatohepatitis inducible hepatokine and regulator of glycemic control
Source: Nat Commun. 2022 Mar 10;13:1259. doi: 10.1038/s41467-022-28889-2 (PMC8913628; doi:10.1038/s41467-022-28889-2)
Supplement: Supplementary file 2 — Description of Additional Supplementary Files [file 41467_2022_28889_MOESM2_ESM.pdf]

Description of Additional Supplementary Files

Title: Supplementary Data 1

Description: All lipidomics data of ARSA-AAV liver (sheet 1), ARSA-AAV liver lipid rafts (sheet 2), ARSA-AAV lipids secreted from liver (sheet 3), ARSA-AAV Plasma (sheet 4) and ARSA-AAV quadriceps muscle lipid rafts (sheet 5).

Title: Supplementary Data 2

Description: All proteomics data of the NASH intracellular hepatocyte proteome and hepatocyte secretome in MCD and CHOL mice, and their respective Control groups (sheets 1 and 2), as well a proteomics data of ARSA-AAV Quadriceps lipid rafts (sheet 3).
