# Supplementary material for: Deep proteomic profiling unveils arylsulfatase A as a non-alcoholic steatohepatitis inducible hepatokine and regulator of glycemic control
Source: Nat Commun. 2022 Mar 10;13:1259. doi: 10.1038/s41467-022-28889-2 (PMC8913628; doi:10.1038/s41467-022-28889-2)
Supplement: Supplementary file 5 — Reporting Summary [file 41467_2022_28889_MOESM5_ESM.pdf]

nature portfolio

Corresponding author(s): Magdalene K Montgomery
Matthew J Watt

Last updated by author(s): Jan 21, 2022

Reporting Summary

Nature Portfolio wishes to improve the reproducibility of the work that we publish. This form provides structure for consistency and transparency in reporting. For further information on Nature Portfolio policies, see our Editorial Policies and the Editorial Policy Checklist.

Statistics

For all statistical analyses, confirm that the following items are present in the figure legend, table legend, main text, or Methods section.

n/a	Confirmed	
☐	☒	The exact sample size (*n*) for each experimental group/condition, given as a discrete number and unit of measurement
☐	☒	A statement on whether measurements were taken from distinct samples or whether the same sample was measured repeatedly
☐	☒	The statistical test(s) used AND whether they are one- or two-sided<br>*Only common tests should be described solely by name; describe more complex techniques in the Methods section.*
☒	☐	A description of all covariates tested
☐	☒	A description of any assumptions or corrections, such as tests of normality and adjustment for multiple comparisons
☐	☒	A full description of the statistical parameters including central tendency (e.g. means) or other basic estimates (e.g. regression coefficient) AND variation (e.g. standard deviation) or associated estimates of uncertainty (e.g. confidence intervals)
☐	☒	For null hypothesis testing, the test statistic (e.g. *F*, *t*, *r*) with confidence intervals, effect sizes, degrees of freedom and *P* value noted<br>*Give P values as exact values whenever suitable.*
☒	☐	For Bayesian analysis, information on the choice of priors and Markov chain Monte Carlo settings
☐	☒	For hierarchical and complex designs, identification of the appropriate level for tests and full reporting of outcomes
☐	☒	Estimates of effect sizes (e.g. Cohen's *d*, Pearson's *r*), indicating how they were calculated

Our web collection on statistics for biologists contains articles on many of the points above.

Software and code

Policy information about availability of computer code

Data collection	Biorad Image Lab (version 6.1), Bio-Rad CFX Manager 3.1 (version 3.1.1517.0823), Exactive MS series Instrument Control Software (version 2.12 QF1), Xcalibur (version 4.3)
Data analysis	Perseus (version 1.5.0.40), Bio-Rad Image Lab (version 6.1), Graphpad Prism (version 7.04), ImageJ (version 1.52a), QIAGEN Ingenuity Pathway Analysis (QIAGEN IPA), Bio-Rad CFX Manager 3.1 (version 3.1.1517.0823), Skyline (version 19.1.0.193, version 21.1.0), MaxQuant (version 1.5.3.30), LipidSearch (version 4.2.23), Xcalibur (version 4.3)

For manuscripts utilizing custom algorithms or software that are central to the research but not yet described in published literature, software must be made available to editors and reviewers. We strongly encourage code deposition in a community repository (e.g. GitHub). See the Nature Portfolio guidelines for submitting code & software for further information.

Data

Policy information about availability of data

All manuscripts must include a data availability statement. This statement should provide the following information, where applicable:

- Accession codes, unique identifiers, or web links for publicly available datasets
- A description of any restrictions on data availability
- For clinical datasets or third party data, please ensure that the statement adheres to our policy

Data Availability statement within main manuscript:

All uncropped immunoblotting images and all source data can be found within the supplementary files. Similarly, proteomics and lipidomics data are supplied as supplementary excel files. In addition, all proteomics datasets generated and analysed during the current study are available in the ProteomeXchange repository, as

follows: Set 1: Project Name: Analysis of mouse liver secretome and proteome during nonalcoholic steatohepatitis [Project accession: PXD024673]; Set 2: Proteomic analysis of ARSA overexpression [Project accession: PXD026280]. The clinical data on patients utilized for assessment of ARSA secretion are available as group averages. All further data generated and analysed during this study are included in this published article, and its supplementary information files.

Field-specific reporting

Please select the one below that is the best fit for your research. If you are not sure, read the appropriate sections before making your selection.

☒ Life sciences ☐ Behavioural & social sciences ☐ Ecological, evolutionary & environmental sciences

For a reference copy of the document with all sections, see nature.com/documents/nr-reporting-summary-flat.pdf

Life sciences study design

All studies must disclose on these points even when the disclosure is negative.

Sample size

1. Mouse studies:

The primary endpoint in these studies was glucose clearance and insulin action. For example, a significant improvement in glucose tolerance by area under the curve from a 90 min GTT is ~ 150 mmol/L (e.g. control ~ 1580 vs. intervention ~1430 mmol/L; standard deviation = 120). The required number of observations to power this analysis (α=0.05, ß=0.8) = ((2 x 1002) / 1502) x 7.85 = 7. Hence, 7 mice are required per group. Previous experience indicates an 80% success rate with performing these studies; hence, 7-9 mice were used per group. In addition, the AAV overexpression/knockdown success rates are 80%, therefore in some instances (depending on validation of successful AAV incorporation) 7-11 mice were used.

2. Cell culture studies:

The primary endpoint in the cell-based studies was insulin signalling and lipid/glucose metabolism. In these experiment, we did not perform a statistical sample size calculation. From our previous +15 years of experience in the assessment of the phosphorylation status of components of the insulin signalling cascade in cells following insulin stimulation, we expected a 5-30-fold change in phosphorylation status and were confident that n=5-6 per group was sufficient to evoke a statistical difference (if one was physiologically present).

3. Human studies:

For assessment of ARSA gene expression, all eligible obese patients undergoing bariatric surgery were prospectively enrolled, while for assessment of ARSA secretion, all eligible obese patients with likely NAFLD were recruited. For each patient cohort, the final sample size per group (No-NAFLD, NAFL, NASH) was dependent on subsequent histopathological assessment of the patient's livers in respect to presence of steatosis, inflammation, hepatocyte ballooning and fibrosis. This histopathological assessment resulted in sample sizes of n=10-66, depending on the hepatic variable reported.

Data exclusions

Data exclusions were pre-established for the following studies/experimental outcomes:
1. Glucose tolerance testing: Mice were excluded when no increase in blood glucose was observed 15 min after oral glucose administration.
2. Insulin tolerance testing: Mice were excluded if no decrease in blood glucose was observed 30 min after i.p. insulin injection.
3. AAV studies: Mice were excluded if overexpression or knockdown was unsuccessful, as indicated by gene or protein expression analysis. Of note, the AAV iv. success rate is 80% highlighting the likelihood that 1-2 mice were excluded from each cohort.
4. ARSA shRNA with/without PF8380 autotaxin inhibition: An insulin tolerance test was conducted in mice in the absence or presence of autotaxin inhibition. Plasma autotaxin activity was assessed after the conclusion of the ITT. Only mice where the inhibitor was successful in reducing plasma autotaxin activity were included in the final ITT analysis.

Replication

For recombinant ARSA experiments in mice, impact of ARSA on GTT was carried out in two experimental cohorts per diet, and we were able to reproduce the effects in both Chow and high-fat diet mice. For AAV overexpression and knockdown experiments, two independent cohorts of mice were utilized, and the effects on glucose tolerance and insulin action were reproduced. For LPA assessment, the results were reproduced utilizing both ELISA and mass spectrometry methods.

For experiments other than those primary outcome measures mentioned above, we took the following general measures to verify the reproducibility of the experimental findings. Cell culture studies for each reported set of experiments were carried out on 2-3 individual days to confirm reproducibility. For studies involving ARSA recombinant protein, we used at least two individual batches of protein to confirm its metabolic effects. These attempts at replication were successful.

In addition, some findings that were not replicated, as follows. For the initial studies on the NASH secretome, mice were fed for an extensive time period of 10 months, followed by hepatocyte isolation and mass spectrometry assessment of the intracellular and secreted proteome. Due to the extensive feeding regime, these experiments were not replicated a second time. Assessment of ARSA gene expression and secretion from human liver slices was carried out in 126 and 94 patients, respectively. Patients were recruited for these studies over a 3-year period, and we do not have the capacity to confirm these results in a second cohort of that size.

Randomization

In all murine studies (including subsequent tissue processing for all outcome measures), body weight was recorded and a glucose tolerance test was performed before mice were allocated to their respective groups. Mice were then matched for body weight and glucose tolerance, and the treatment regime was initiated. For the human studies, we prospectively enrolled all eligible obese patients undergoing bariatric surgery. Liver histopathological scoring was obtained following the bariatric procedure and patients were grouped according to their liver

	phenotype. For all cell culture studies, experimental 'wells' were chosen randomly followed by incubation of such cells/wells with conditioned media, ARSA protein and/or insulin.
Blinding	Research staff were blinded to group allocation during the data collection and analysis stage for most experiments, after which data was de-identified by the senior investigators. For all AAV experiments in mice, AAV administration was carried out by the senior investigators, and the students/staff performing the in vivo metabolic phenotyping experiments (including MRI, GTT, ITT, Promethion) were blinded to the mouse grouping. Researchers were also blinded during processing of tissues and plasma samples (both mouse and human), as well as tissue homogenates/lysates for mass spectrometry, RNA extractions and qPCR, ELISA assays and glucose uptake experiments.<br><br>Researchers were not blinded when performing the initial mouse NASH feeding studies, as the phenotypical differences between Control and NASH mice (e.g. obvious change in body weight) and the food pellet color of the different diets prevented this. Researchers were also not blinded to the groupings when performing immunoblotting experiments (for both mouse tissues and cell culture lysates) as the westerns were run in a specific sequence (e.g. 2x Control, 2x NASH, etc.) to allow for representative immunoblot images. Lastly, for radioactive tracer experiments in cells, the entire experiments were carried out by one staff member/student, e.g. from the addition of ARSA protein to the wells, to the subsequent assessment of glucose uptake (done within the same day).

Reporting for specific materials, systems and methods

We require information from authors about some types of materials, experimental systems and methods used in many studies. Here, indicate whether each material, system or method listed is relevant to your study. If you are not sure if a list item applies to your research, read the appropriate section before selecting a response.

Materials & experimental systems

n/a	Involved in the study
☐	☒ Antibodies
☐	☒ Eukaryotic cell lines
☒	☐ Palaeontology and archaeology
☐	☒ Animals and other organisms
☐	☒ Human research participants
☐	☒ Clinical data
☒	☐ Dual use research of concern

Methods

n/a	Involved in the study
☒	☐ ChIP-seq
☒	☐ Flow cytometry
☒	☐ MRI-based neuroimaging

Antibodies

Antibodies used	Akt Cell Signalling 9272S<br>Akt (S473) Cell Signalling 4058S<br>ADRB3 Abcam ab94506<br>ARSA Abcam ab174844<br>beta actin Abcam ab3280<br>Erk (T202/Y204) Cell Signalling 9101S<br>GSK3α/β Cell Signalling 5676S<br>GSK3α/β (S21/9) Cell Signalling 8566S<br>IR Cell Signalling 3020S<br>IR (T1158/1162/1163) Upstate 07-841<br>IRE1 Cell Signalling 3294S<br>IRS1 Cell Signalling 3407S<br>IRS1 (Y612) Sigma 12658<br>Na,K-ATPase Cell Signalling 3010S<br>cPLA2 Santa Cruz sc-454<br>cPLA2 (S505) Abcam ab53105<br>Thy1 Cell Signalling 9798<br><br>ALL ANTIBODIES HAVE BEEN USED AT A DILUTION OF 1:1,000.
Validation	All antibodies listed above were utilized solely for immunoblotting purposes. All uncropped images are supplied with this manuscript. The majority of antibodies from Cell Signalling, Abcam and Santa Cruz have been used by our team for many years, and have been previously published on:<br>1. PMID: 30617219 (Diabetes 2019 68(3):543-555): beta actin, Akt, Akt S473, Na,K-ATPase, IRS1, IRS1 (Y612), IRE1<br>2. PMID: 32878981 (Sci Transl Med 2020 12(559):eaaz8048): Akt, Akt S473, IRE1, GSK3α/β S21/9, GSK3α/β, Erk T202/Y204<br>3. PMID: 31533003 (FASEB J 2019 33(12):13267-13279): beta actin, ADRB3, Na,K-ATPase<br>4. PMID: 30728288 (Sci Transl Med 2019 11(478):eaau5758): cPLA2<br>5. PMID: 34800307 (FASEB J 2021 35(12):e22046): beta actin, Na,K-ATPase, Thy1.<br>The Thy1 antibody was also tested by Cell Signalling using extracts from human, mouse, and rat brain.<br><br>The anti-ARSA antibody from Abcam (ab174844) has been validated by the manufacturer at a 1/1000 dilution (as used by us) in human skin lysates, A431 cell lysates and HeLa cell lysates, showing a specific band at 54kDa. In addition, we validated the antibody

in-house using murine recombinant ARSA, either by loading recombinant ARSA directly in the western blot, or by injecting mice with recombinant ARSA, followed by immunoblotting assessment of plasma ARSA. This antibody has also been used in a recent 2020 publication (PMID: 32221382).

The IR antibody has been tested by Cell Signaling (i.e., the manufacturer) using western blot analysis of cell lysates from CHO and CHO/IR cells transfected with the insulin receptor, at a dilution of 1:1,000 (as done by us). In addition, this antibody has been utilized in >25 publications, including (but not limited to): PMID: 33258073, PMID: 33052961, PMID: 33036464, PMID: 31693448, PMID: 32478834. As shown in Figure S2 and in the uncropped immunoblotting image source file, this antibody provides one specific band at exactly the correct MW size of 95kDa.

The IR (T1158/1162/1163) antibody has been tested by Upstate (i.e., the manufacturer) using insulin-stimulated CHO cells transfected with the insulin receptor, in the absence or presence of insulin stimulation. In addition, the manufacturer used a blocking peptide immunogen corresponding to IR (Tyr1162/Tyr1163) and a blocking peptide immunogen corresponding to phospho-IR (Tyr1158/Tyr1162/Tyr1163) to validate the specificity of this antibody. This antibody has been recently used in a patent application (WO2017042242A1). In addition, we validated this antibody in-house in basal and insulin-treated 3T3 adipocytes, C2C12 myotubes and primary hepatocytes. As shown in Figure S2 and within the uncropped immunoblotting source file, this antibody shows a specific band at 95kDa, which is highly sensitive to insulin stimulation, further highlighting its specificity.

The cPLA2 (S505) antibody was tested by Abcam using extracts from HeLa cells treated with TNF-a in the absence or presence of immunising peptide, showing specificity at 85kDa. In addition, this antibody has been previously used in >15 publications, including (but not limited to): PMID: 34078224, PMID: 33335297, PMID: 31569627, PMID: 30317637.

Eukaryotic cell lines

Policy information about cell lines

Cell line source(s)	Primary murine hepatocytes were isolated in-house from lean chow mice by Dr Montgomery.<br>C2C12 myoblasts and 3T3-L1 fibroblasts were obtained from ATCC.<br>Expi293 cells were obtained from Thermo Fisher.
Authentication	For primary murine experiments, the purity of the hepatocyte fraction has been previously established in our laboratory (Meex et al. Cell Metab (2015) 22(6):1078-89).<br>For 3T3-L1 adipocytes, cells change from a fibroblast-like appearance to an adipocyte-like structure during the differentiation procedure, with differentiated cells accumulating lipids in the form of lipid droplets that grow in number and size over cultivation time. Only cells with extensive lipid droplet accumulation (as determined by brightfield microscopy) at day 7 post-differentiation initiation were utilized for subsequent experiments.<br>Similarly, C2C12 myoblasts show enhanced myotube formation upon serum-starvation. Only cells with significant myotube formation at Day 6 post-differentiation (as determined by brightfield microscopy) were utilized in subsequent experiments.<br>Expi293 cells were utilized for the purpose of ARSA protein production. These cells are highly transfectable and generate superior protein yields, maintaining this capacity for >40 passages. After being obtained from Thermo Fisher, this cell line was not further authenticated.
Mycoplasma contamination	The cell lines were not tested for mycoplasma contamination.
Commonly misidentified lines<br>(See ICLAC register)	Neither 3T3-L1, C2C12 or Expi293 cells are listed within the ICLAC NCBI BioSample register.

Animals and other organisms

Policy information about studies involving animals; ARRIVE guidelines recommended for reporting animal research

Laboratory animals	1. 8-10 week old (age at study commencement) male C57BL/6J, sourced from Monash University MARP breeding facility, or Animal Resources Centre, Canning Vale, Australia.<br>2. 8-10 week old (age at study commencement) male db/db mice, bred in-house, but originally obtained from Jackson Laboratory (strain: BKS.Cg-Dock7m +/+ Leprdb/J).
Wild animals	This study did not involve wild animals.
Field-collected samples	This study did not involve samples collected from the field.
Ethics oversight	All mouse experiments were approved by the Monash University Animal Ethics Committee (MARP/2016/073) and the University of Melbourne Anatomy & Neuroscience, Pathology, Pharmacology, and Physiology Animal Ethics Committee (ethics ID 1814403), and conformed to the National Health and Medical Research Council of Australia guidelines regarding the care and use of experimental animals.

Note that full information on the approval of the study protocol must also be provided in the manuscript.

Human research participants

Policy information about studies involving human research participants

Population characteristics	For gene expression studies: Inclusion criteria were (1) age≥18 years, (2) BMI≥35 kg/m2 and (3) alanine aminotransferase

	(ALT) or aspartate aminotransferase (AST) >0.5 times upper limit normal (ULN). Patients were excluded if they had evidence of other liver disease, including viral hepatitis, medication-related, autoimmune, familial/genetic causes or a history of excessive alcohol use, as defined by the American Association for the Study of Liver Diseases.<br><br>For liver secretion studies: Bariatric patients with likely NAFLD were recruited, including previous history of NAFLD/NASH and presence of metabolic comorbidities (type 2 diabetes, hypertension, obstructive sleep apnea). Exclusion criteria included: age <18 years; current or past excessive ethanol use (>210 g/week males, >140 g/week females); other causes of chronic liver disease and/or hepatic steatosis including Wilson's disease, alpha-1-antitrypsin deficiency; viral hepatitis; primary biliary cirrhosis; autoimmune hepatitis; genetic iron overload; hypo- or hyperthyroidism; coeliac disease; hepatitis B/C; human immunodeficiency virus; recent (within 3 months of screening visit) or concomitant use of agents known to cause hepatic steatosis including corticosteroids, amiodarone, methotrexate, tamoxifen, valproic acid, and high dose oestrogens. Pre-operative clinical details were collected using a questionnaire, including metabolic comorbidities, screening for alternate causes for liver disease (as detailed above in exclusion criteria), medications, social and family history. Patients fasted overnight and blood tests were taken prior to surgery for assessment of plasma ALT and AST.
Recruitment	For gene expression studies, all eligible obese patients undergoing bariatric surgery were prospectively enrolled in three metropolitan hospitals in Melbourne (Australia) between July 2015 and August 2017.<br>For liver secretion studies, eligible patients with obesity scheduled for bariatric surgery were screened to participate and subjects provided informed written consent, and a detailed medical history was taken.<br>As all eligible subjects were allowed to decide entirely for themselves whether or not to participate in this study, a self-selection bias in the reported data cannot be ruled out. In addition, as all patients in this study were undergoing bariatric surgery, this patient population was obese and therefore does not represent a snapshot of the general population.
Ethics oversight	For hepatic ARSA secretion studies, ethics was obtained from the University of Melbourne Human Ethics Committee (ethics ID 1851533) and approved by The Avenue Hospital Human Research Ethics Committee (ethics ID WD00006, HREC reference number 249) and the Alfred Hospital Human Research Ethics Committee (ethics ID GO00005).This study has been approved as a 'Biospecimen analysis research' and is not considered 'Interventional/Clinical Trial research', thereby not requiring a clinical trial registration.<br><br>For hepatic mRNA expression analysis, ethics approval was obtained from relevant Human Research Ethics Committee (Alfred (195/15), Avenue (190) and Cabrini (09-31-08-15)), and the study was registered with the Australian Clinical Trials Register (ACTRN12615000875505: Non-invasive diagnosis and monitoring of non-alcoholic fatty liver disease in bariatric surgical patients).

Note that full information on the approval of the study protocol must also be provided in the manuscript.

Clinical data

Policy information about clinical studies
All manuscripts should comply with the ICMJEguidelines for publication of clinical research and a completedCONSORT checklist must be included with all submissions.

Clinical trial registration	ACTRN12615000875505
Study protocol	Details on the clinical trial can be accessed here:<br>https://www.anzctr.org.au/Trial/Registration/TrialReview.aspx?id=369051&isReview=true<br><br>The full trial protocol is not available. However, protocol details can be found in the following publications:<br><br>- Geraldine J Ooi , Paul R Burton, Jacqueline Bayliss, Arthe Raajendiran, Arul Earnest, Cheryl Laurie, William W Kemp, Catriona A McLean, Stuart K Roberts, Matthew J Watt, Wendy A Brown (2019) Effect of Body Mass Index, Metabolic Health and Adipose Tissue Inflammation on the Severity of Non-alcoholic Fatty Liver Disease in Bariatric Surgical Patients: a Prospective Study. 29(1):99-108. Obes Surg<br><br>- Geraldine J Ooi, Andrew Clouston, Yazmin Johari, William W Kemp, Stuart K Roberts, Wendy A Brown, Paul R Burton (2021) Evaluation of the histological variability of core and wedge biopsies in nonalcoholic fatty liver disease in bariatric surgical patients. Surg Endosc 35(3):1210-1218<br><br>- Geraldine J Ooi, Peter J Meikle, Kevin Huynh, Arul Earnest, Stuart K Roberts, William Kemp, Benjamin L Parker, Wendy Brown, Paul Burton, Matthew J Watt (2019) Hepatic lipidomic remodeling in severe obesity manifests with steatosis and does not evolve with non-alcoholic steatohepatitis. J Hepatol 75(3):524-535
Data collection	Data were collected at the Avenue or Cabrini Hospital (Melbourne, Australia), and stored at the Center of Obesity Research and Education (CORE) at the Alfred Centre (Melbourne, Australia). Recruitment started 06-2015 to 12-2017.
Outcomes	The primary outcomes of this study were the efficacy of transient elastography, magnetic resonance spectroscopy (MRS) and serum biomarkers in measuring NAFLD in a bariatric cohort by comparison with liver biopsy. The secondary outcomes were a change in NAFLD with surgically induced weight loss as measured by liver biopsy, and the correlation of mRNA profile of adipose tissue with liver biopsy in patients with NAFLD. We prospectively enrolled all eligible patients with obesity undergoing bariatric surgery, who fit criteria for likely NAFLD, including AST or ALT > 0.5 upper limit normal, GGT > upper limit normal, abnormal transient elastography, and/or abnormal ultrasound suggesting NAFLD. All enrolled patients underwent a MRS scan (at baseline and 1 year after bariatric

surgery), a transient elastography/Fibroscan (at baseline, and 3 and 12 months after bariatric surgery) and bloods were taken for routine blood tests (at baseline and 1, 3 and 12 months after bariatric surgery). A liver core biopsy as well as a small wedge biopsy were taken during the bariatric procedure (at baseline), amounting to a total of less than 1cm3 of liver tissue. In addition, a piece of visceral and subcutaneous fat was taken during the operation from the omentum around the area of surgery. For patients with >33% steatosis, any inflammation or any fibrosis were offered a follow-up percutaneous liver biopsy at 12 months. Participants provided written informed consent, in the absence of any form of compensation.
